# Revision of the Southeast Asian millipede genus *Orthomorpha* Bollman, 1893, with the proposal of a new genus (Diplopoda, Polydesmida, Paradoxosomatidae)

**DOI:** 10.3897/zookeys.131.1921

**Published:** 2011-09-29

**Authors:** Natdanai Likhitrakarn, Sergei I. Golovatch, Somsak Panha

**Affiliations:** 1 Animal Systematics Research Unit, Department of Biology, Faculty of Science, Chulalongkorn University, Bangkok, 10330, Thailand; 2 Institute for Problems of Ecology and Evolution, Russian Academy of Sciences, Leninsky pr. 33, Moscow 119071, Russia

**Keywords:** millipede, *Orthomorpha*, taxonomy, new genus, new species, key, Thailand, Malaysia, Myanmar, Indochina, Indonesia, Seychelles

## Abstract

The large genus *Orthomorpha* is rediagnosed and is shown to currently comprise 51 identifiable species ranging from northern Myanmar and Thailand in the Northwest to Lombok Island, Indonesia in the Southeast. Of them, 20 species have been revised and/or abundantly illustrated, based on a restudy of mostly type material; further 12 species are described as new: *Orthomorpha atypica* **sp. n.**, *Orthomorpha communis* **sp. n.**, *Orthomorpha isarankurai* **sp. n.**, *Orthomorpha picturata* **sp. n.**, *Orthomorpha similanensis* **sp. n.**, *Orthomorpha suberecta* **sp. n.**, *Orthomorpha tuberculifera* **sp. n.**,*Orthomorpha subtuberculifera* **sp. n.** and *Orthomorpha latiterga* **sp. n.**, all from Thailand, as well as *Orthomorpha elevata* **sp. n.**,*Orthomorpha spiniformis* **sp. n.** and *Orthomorpha subelevata* **sp. n.**, from northern Malaysia. The type-species *Orthomorpha beaumontii* (Le Guillou, 1841) is redescribed in due detail from male material as well, actually being a senior subjective synonym of *Orthomorpha spinala* (Attems, 1932), **syn. n.** Two additional new synonymies are proposed: *Orthomorpha rotundicollis* (Attems, 1937) = *Orthomorpha tuberculata* (Attems, 1937), **syn. n.**, and *Orthomorpha butteli* Carl, 1922 = *Orthomorpha consocius* Chamberlin, 1945, **syn. n.**, the valid names to the left. All species have been keyed and all new and some especially widespread species have been mapped. Further six species, including two revised from type material, are still to be considered dubious, mostly because their paraterga appear to be too narrow to represent *Orthomorpha* species. A new genus, *Orthomorphoides* **gen. n.**, diagnosed versus *Orthomorpha* through only moderately well developed paraterga, coupled with a poorly bi- or trifid gonopod tip, with at least some of its apical prongs being short spines, is erected for two species: *Orthomorpha setosus* (Attems, 1937), the type-species, which is also revised from type material, and *Orthomorpha exaratus* (Attems, 1953), both **comb. n.** ex *Orthomorpha*.

## Introduction

**Map 1.**
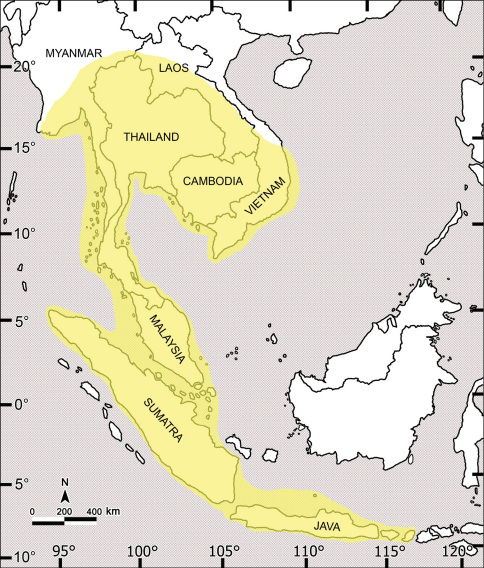
Distribution of the genus *Orthomorpha*.

The genus *Orthomorpha* Bollman, 1893 is one of the largest amongst the paradoxosomatid millipedes, dominating the Oriental fauna and ranging from Myanmar in the west, through the entire Indochina Peninsula, to Lombok, Indonesia in the east (Map 1). According to Golovatch (1998), this genus currently contains 44 described species in six species groups. Only a few relevant changes have since been made, i.e. the synonymization of *Orthomorpha flavocarinata* (Daday, 1889) with *Orthomorpha coarctata* (De Saussure, 1860) (Enghoff 2005), the addition of *Orthomorpha intercedens* Attems, 1937 to *Orthomorpha* (Enghoff 2005), as well as a review of the *sericata*-group (Likhitrakarn et al. 2010a). Concerning the status of *Orthomorpha intercedens*, Enghoff (2005), Decker (2010) and Likhitrakarn et al. (2010a) must have overlooked the fact that Jeekel (1970) had synonymized it with *Orthomorpha insularis* Pocock, 1895. Considering four new species added very recently to the *sericata*-group (Likhitrakarn et al. 2010a), five further species to be ejected (see below), as well as 12 new species described below, at present the total amounts to 51 species.

Ever since its proposal, the name *Orthomorpha* has been jeopardized, because its type-species, *Orthomorpha beaumontii* (Le Guillou, 1841), has only been known from a single female said to have come from “Borneo”. Despite Jeekel’s (1963) redescription and numerous illustrations of the holotype, the identity of *Orthomorpha beaumontii* has heretofore remained rather obscure, thus threatening the status of the entire genus (Jeekel 1963, 1968; Golovatch 1998). To make it worse, the holotype must have been mislabeled, as no indigenous species of *Orthomorpha* are known yet from Borneo (Golovatch 1996).

The present paper continues our studies on the still quite poorly known diplopod fauna of Southeast Asia (Golovatch et al. 2009a, 2009b; Golovatch 2009; Likhitrakarn et al. 2010a, 2010b etc.). This time we offer an updated revision of *Orthomorpha*, based on a restudy of numerous relevant types, as well as on abundant material collected recently in Thailand and Malaysia. This has allowed us not only to considerably refine the scope of the genus, including 12 new species, a few new transfers and synonymies, but also to establish the true identity of its type-species through finding the best match to its holotype.

Among the species groups currently delimited in *Orthomorpha*, none appears to be firmly based. Such basic characters as the presence/absence and shape of a sternal lobe or cones between ♂ coxae 4, as well as the shape of the gonopod tip not only fail to correlate with one another, but also vary too considerably, no longer allowing for a clear-cut distinction between the species groups to be made. The abundant material (re)studied for this project shows all possible transitions, merging and blurring the borders between such groups. Instead, we can only outline certain trends in the development of the above and some other basic structures.

Unlike most of the genera of Polydesmida, including Paradoxosomatidae, *Orthomorpha* has long been acknowledged as a group showing surprisingly uniform gonopods (Jeekel 1963). Whereas species of most of the paradoxosomatid genera are rich in gonopod characters, e.g. *Tylopus* Jeekel, 1968 (tribe Sulciferini) (Likhitrakarn et al. 2010b), with somatic features often being rather subordinate, *Orthomorpha* species usually present only a few or no meaningful traits for their separation. Instead, the peripheral structures such as tegument texture, paraterga, pleurosternal carinae etc. show a remarkably wide range of variation. Sometimes broad variation in somatic characters is observed not only between species, but infraspecifically as well, as demonstrated for *Orthomorpha insularis* by Jeekel (1970) or for *Orthomorpha enghoffi* Likhitrakarn, Golovatch & Panha, 2010 by Likhitrakarn et al. (2010a).

So we are inclined to abandon species group delimitation in *Orthomorpha* altogether. Instead, we arrange the species mainly on the structure of the gonopod tip, however few characters it offers. This approach better agrees with (Jeekel (1963, 1964), who put special emphasis on gonopod conformation, than with Hoffman (1977), who mostly relied on the structure of the sternal lobe or cones, if any, between ♂ coxae 4 when grouping species. Here we prefer to regard *Orthomorpha* as a member of the tribe Orthomorphini perhaps especially similar to the largely sympatric genus *Antheromorpha* Jeekel, 1968, which normally shows similarly large size and paraterga, but a very deeply bifid gonopod tip (see also below). In *Orthomorpha*, the latter is always poorly bi- or trifid, some of the apical prongs being either minute denticles, or rounded lappets, or small teeth, or completely reduced. Variations are few, but, together with certain peripheral characters, they generally offer enough grounds for a reliable discrimination of species.

*Orthomorpha* is the type genus of the tribe Orthomorphini, which has rather recently been reviewed and shown to currently contain 19 genera, nearly all of them keyed (Golovatch 1997a, 2000, 2009).

Because an analysis of the phylogeny of either *Orthomorpha* or Orthomorphini has never been attempted, below we arrange the species of *Orthomorpha*, according to the degree of gonopod tip complexity. The former *coarctata*-group, with only two constituent nominate species or subspecies, shows the tip bearing only a single terminal lappet. For this reason, *Orthomorpha coarctata* (De Saussure, 1860), the only pantropical representative of *Orthomorpha*, has often been referred to as a distinct genus, *Asiomorpha* Verhoeff, 1939 (e.g. Hoffman 1980; Korsós 2004; Enghoff 2005). Yet, following (Jeekel (1963, 1968, 1970), we prefer to regard *Orthomorpha coarctata* as a species of *Orthomorpha*. Several further congeners that demonstrate a bifid tip will be treated next, followed by those, in which the tip is trifid. However, because the identity of the type-species has heretofore remained obscure, the taxonomic part below will start with a diagnosis of *Orthomorpha*, followed by a solution of the problem concerning *Orthomorpha beaumontii*.

Most of the older species revised, especially those valid ones in which the original descriptions appear to be deficient, are redescribed and illustrated here in sufficient detail to ensure their easy recognition.

Golovatch (1998) keyed 42 species of *Orthomorpha* which he considered valid. Likhitrakarn et al. (2010a) added another four species. Below is a revised list of the 51 *Orthomorpha* species arranged in alphabetic order, all being keyed at the end of the paper.

*Orthomorpha alutaria* Likhitrakarn, Golovatch & Panha, 2010

*Orthomorpha arboricola* (Attems, 1937)

*Orthomorpha asticta* Likhitrakarn, Golovatch & Panha, 2010

*Orthomorpha atypica* sp. n.

*Orthomorpha baliorum* Golovatch, 1995

*Orthomorpha banglangensis* Golovatch, 1998

*Orthomorpha beaumontii* (Le Guillou, 1841)

*Orthomorpha beroni* Golovatch, 1997

*Orthomorpha bipunctata* (Sinclair, 1901)

*Orthomorpha butteli* Carl, 1922

*Orthomorpha cambodjana* (Attems, 1953)

*Orthomorpha coarctata* (De Saussure, 1860)

*Orthomorpha communis* sp. n.

*Orthomorpha conspicua* (Pocock, 1894)

*Orthomorpha crucifer* (Pocock, 1889)

*Orthomorpha elevata* sp. n.

*Orthomorpha enghoffi* Likhitrakarn, Golovatch & Panha, 2010

*Orthomorpha flaviventer* (Attems, 1898)

*Orthomorpha fluminoris* Hoffman, 1977

*Orthomorpha francisca* Attems, 1930

*Orthomorpha fuscocollaris* Pocock, 1895

*Orthomorpha glandulosa* (Attems, 1937)

*Orthomorpha horologiformis* Golovatch, 1998

*Orthomorpha hydrobiologica* Attems, 1930

*Orthomorpha insularis* Pocock, 1895

*Orthomorpha isarankurai* sp. n.

*Orthomorpha karschi* (Pocock, 1889)

*Orthomorpha latiterga* sp. n.

*Orthomorpha lauta* Golovatch, 1998

*Orthomorpha melischi* Golovatch, 1997

*Orthomorpha murphyi* Hoffman, 1977

*Orthomorpha parasericata* Likhitrakarn, Golovatch & Panha, 2010

*Orthomorpha paviei* Brölemann, 1896

*Orthomorpha picturata* sp. n.

*Orthomorpha pterygota* Golovatch, 1998

*Orthomorpha rotundicollis* (Attems, 1937)

*Orthomorpha scabra* Jeekel, 1964

*Orthomorpha sericata* Jeekel, 1964

*Orthomorpha similanensis* sp. n.

*Orthomorpha spiniformis* sp. n.

*Orthomorpha subelevata* sp. n.

*Orthomorpha suberecta* sp. n.

*Orthomorpha subkarschi* Golovatch, 1998

*Orthomorpha subsericata* Golovatch, 1998

*Orthomorpha subtuberculifera* sp. n.

*Orthomorpha tenuipes* (Attems, 1898)

*Orthomorpha thalebanica* Golovatch, 1998

*Orthomorpha tuberculifera* sp. n.

*Orthomorpha unicolor* Attems, 1930

*Orthomorpha weberi* (Pocock, 1894)

*Orthomorpha zehntneri* (Carl, 1902)

## Material and methods

New material derives from throughout Thailand and from northern Malaysia, taken from 1962 to 2011. Coloration was photographed in the laboratory (both live and alcohol material) for all of the encountered species. Material was then preserved in 75% ethanol and studied in the laboratory using an Olympus stereomicroscope. Scanning electron micrographs (SEM) were taken of uncoated specimens using a JEOL, JSM–5410 LV microscope. After SEM examination of the gonopods, they were returned to alcohol. Material of each of the species available for (re)study was photographed, the digital images assembled using the automontage software techniques, while gonopods also redrawn. Specimens were received from the following museum collections:

AMNH American Museum of Natural History, New York, USA,

CUMZ Museum of Zoology, Chulalongkorn University, Bangkok, Thailand,

NHML Natural History Museum, London, Great Britain,

NHMW Naturhistorisches Museum Wien, Austria,

MHNG Muséum d’histoire naturelle, Genève, Switzerland,

ZMUM Zoological Museum, State University of Moscow, Russia,

ZMUC Natural History Museum of Denmark, University of Copenhagen, Denmark.

So as not to repeat information, diagnoses are only provided here for new species, because the key below will show the main distinctions for all of the species in the genus.

In the catalogue sections, D stands for the original description, subsequent descriptive notes or appearance in a key, R for a subsequent record or records, N for giving a new name, and M for a mere mention. Not all of the relevant references are being quoted under certain Indochinese species, because there is no reason for duplicating the regional catalogues available and still valid for the millipedes of Vietnam and Thailand (Enghoff et al. 2004; Enghoff 2005).

A dynamic web page for each taxon name mentioned in the paper is generated on the fly by the Pensoft Taxon Profile tool (see Penev et al. 2010). All species descriptions are automatically exported at the time of publication to a wiki platform (www.species-id.net) through the Pensoft Wiki Convertor (see Penev et al. 2011, Stoev and Enghoff 2011).

## Taxonomic part

### Orthomorpha

Bollman, 1893

http://species-id.net/wiki/Orthomorpha

Orthomorpha Bollman 1893: 159 (N).
Orthomorpha – Jeekel 1963: 261 (D).

#### Diagnosis.

A genus of Orthomorphini with 20 segments. Body medium- to large-sized, adults ca 15–50 mm long, ca 1.1–3.1 and 1.5–6.7 mm wide on midbody pro- and metazona, respectively. Paraterga invariably well-developed, metazonite to prozonite width ratio being ca 1.6–1.7. Adenostyles on ♂ legs 1 missing. Sternal lobe or cone(s) between ♂ coxae 4 present or absent.

Gonopod with a long, subcylindrical, distodorsally usually setose coxite and a normal, cylindrical cannula. Telopodite mostly very slender and long, modestly curved. Prefemoral portion densely setose, about as long as (rarely) to ca 2–3 times (usually) shorter than femorite (measured together with “postfemoral” part lying distal to lateral sulcus). Femorite without evidence of torsion (= seminal groove running only mesally), often slightly enlarged distally, mostly with a clear-cut, oblique, distolateral sulcus demarcating a “postfemoral” part. Solenophore only moderately strongly curved mesad or caudomesad, consisting of modestly developed laminae lateralis and medialis, yet with lamina lateralis somewhat larger than lamina medialis, both sheathing a similarly long, simple, flagelliform solenomere with a barely exposed tip; tip of solenophore never deeply split, normally poorly bi- or trifid, some of apical prongs being either minute denticles or lappets, or small teeth, or completely reduced.

Type-species: *Polydesmus beaumontii* Le Guillou, 1841, by subsequent designation by Pocock (1909).

#### Remarks.

The Orthomorphini is certainly the most diverse tribe of Oriental Paradoxosomatinae both at the generic and species levels. It is generally characterized by the gonopod showing a simple, usually subcylindrical (= normally not excavate mesally) and elongate femorite devoid both of torsion (= the seminal groove running entirely or nearly entirely on the mesal side) and processes/outgrowths. A distolateral sulcus demarcating a “postfemoral” part is usually, but not always, present. A solenomere is always long and flagelliform, starting on top of the femorite (+ “postfemoral” part, if any) at the base of a more or less elaborate solenophore, the latter being demarcated by an evident cingulum or mesal sulcus. The solenophore always consists of well- to moderately well developed, often subequal, lamellar lamina lateralis and lamina medialis, both only modestly curved mesad or caudomesad and both supporting and sheathing at least most of the solenomere. It is the solenophore that provides most of the generic characters in Orthomorphini, such as the presence or absence of additional structures (processes or lobes) at its base, near midway and/or at its tip (Jeekel 1968; Golovatch 1997a, 2000, 2009).

*Orthomorpha* is basically characterized by very broad paraterga, coupled with the gonopod showing mostly an elongate, slender femorite (+ “postfemoral” part, if any) and a long, modestly curved solenophore bearing additional structures neither at its midway nor near its base; the tip of the solenophore is never deeply split, normally poorly bi- or trifid, some of the apical prongs being either minute denticles or lappets, or small teeth, or completely reduced.

Based on the broad paraterga and the conformation of the solenophore, *Orthomorpha* comes closest to the continental Southeast Asian *Antheromorpha* Jeekel, 1968 (see above), the Philippine *Luzonomorpha* Hoffman, 1973 and the basically Bornean *Gigantomorpha* Jeekel, 1963 (Jeekel 1963, 1980a; Hoffman 1973; Golovatch 1996, 1997a). However, *Antheromorpha* species show a very deeply split tip of the solenophore, *Gigantomorpha* species demonstrate a somewhat flattened and bisinuate gonofemorite, usually also a more elaborate, often deeply split solenophore tip, whereas in *Luzonomorpha* the tip is deeply bispinose. A similarly poorly bi- or trifid gonopod is only observed in *Orthomorphoides* gen. n., but its species differ from *Orthomorpha* in much smaller bodies, in the rather poorly developed paraterga and at least in some of the apical prongs of the solenophore being short spines (see below).

### Orthomorpha beaumontii

(Le Guillou, 1841)

http://species-id.net/wiki/Orthomorpha_beaumontii

Figs 1 [Fig F2] 3

Polydesmus Beaumontii Le Guillou 1841: 279 (D).
Polydesmus Beaumontii – Gervais 1847: 101 (D).
Polydesmus (Parademus) Beaumontii – De Saussure 1859: 326 (D); Humbert & De Saussure 1860: 670 (D).
Orthomorpha beaumonti – Bollman 1893: 196 (M, R).
Orthomorpha hydrobiologica spinala Attems 1932: 39 (D), syn. n.
Orthomorpha spinala – Jeekel 1968: 45 (M); Hoffman 1973: 362 (M); 1977: 700 (M); Golovatch 1998: 42 (D).
Orthomorpha beaumontii – Jeekel 1963: 269 (D); 1968: 45 (M); Golovatch 1997b: 79 (D); 1998: 42 (D).
Prionopeltis sp. (*Beaumontii*) (sic!) – Attems 1898: 357 (D, R) [Non],
Prionopeltis Beaumontii Attems (sic!) – Attems 1914: 207 (M) [nec],
Pratinus beaumontii (Att.) – Attems 1937: 122 (M) [nec].

#### Material examined.

Syntypes of *Orthomorpha hydrobiologica spinala*: 4 ♂, 1 ♀ (NHMW–3510), Indonesia, “Karimon Djawa Inseln” (= Pulau Karimunjawa Island, north of Java), V.1926, leg. Dammerman; 1 ♀ of *Orthomorpha hydrobiologica spinala* (NHMW–8001), Indonesia, Java, Tjibodas, no date, leg. W. S. S. van Benthem-Jutting, det. C. Attems.

#### Redescription.

Length 33–38 mm (♂), 36–38 mm (♀), width of midbody pro- and metazona 2.7–2.8 and 4.0–4.2 mm (♂), 3.1–3.3 and 4.3–4.4 mm (♀), respectively. Coloration of alcohol material upon long-term preservation rather uniformly brown with contrasting pale yellowish paraterga, venter and legs light yellow-brown (Fig. 2).

Head usual, clypeolabral region sparsely setose, surface of vertex smooth; epicranial suture distinct. Antennae moderately long (Fig. 2A & B), reaching behind midway of body segment 3 (♂) or beyond segment 2 (♀). Head in width < collum < segments 3 and 4 < segment 2 < segments 5–16(17), gently and gradually tapering thereafter. Collum with three transverse rows of setae, 4+4 anterior, 2+2 intermediate, and 3+3 posterior setae; caudal corner of paraterga dentiform, pointed, directed caually (Fig. 2A, B & J). Tegument smooth and shining, prozona very finely shagreened, metaterga slightly rugulose; surface below paraterga smooth. Postcollum metaterga with two transverse rows of setae, these being always abraded and traceable as insertion points: 2+2 in anterior (pre-sulcus) row, 3+3 in posterior (postsulcus) one. Axial line barely visible both on pro- and metazona. Paraterga very strongly developed (Fig. 2A-G, J-L), especially so in ♂, subhorizontal, always lying below dorsum, thin in lateral view, like blunt blades, a little thicker only on pore-bearing segments, always clearly projecting well behind tergal margin. Calluses delimited both dorsally and ventrally, only on segment 2 without ventral sulcus, thin, especially so on poreless segments. Paraterga 2 broad, anterior edge rounded, lateral edge with two small, but evident incisions in anterior 1/3; posterior edge evidently concave (Fig. 2A & J). Paraterga 3 and 4 subequal, like subsequent paraterga, anterior edge slightly rounded, bordered and fused to callus, lateral edge with a small incision in anterior third. Paraterga 15–19 with tip of caudal corner evidently curved mesad. Ozopores evident, lateral, lying in an ovoid groove at about 1/3 of metazonital length. Transverse sulcus present on metaterga 5–18, shallow, not reaching bases of paraterga, finely beaded at bottom (Fig. 2A, C, F, J-L). Stricture between pro- and metazona narrow, shallow, beaded at bottom down to base of paraterga (Fig. 2D & E). Pleurosternal carinae complete crests only on segment 2 or segments 2 and 3, with a small, sharp, caudal tooth on segments 3–7(8) (♂) or 4–6 (♀), thereafter with a very small caudal denticle until segment 15 (♂, ♀). Epiproct (Fig. 2E-G & L) conical, flattened dorsoventrally, apical papillae well-developed, acute and directed ventrad; tip subtruncate; pre-apical papillae small, but visible. Hypoproct (Fig. 2G) subtriangular, setiferous knobs at caudal edge well-separated.

Sterna sparsely setose, without modifications, but with a pair of small, rounded, completely separated, setose cones between ♂ coxae 4 (Fig. 2H & I). No conspicuous ridge in front of gonopod aperture. Legs long and slender, slightly incrassate in ♂, midbody ones ca 1.2–1.3 (♂) or 0.8–0.9 times (♀) as long as body height, prefemora without modifications, tarsal brushes present until legs of segment 9.

Gonopods (Fig. 3) simple. Coxa long and slender, with several setae distodorsally. Prefemoral (= densely setose) portion more than 3 times shorter than femorite (measured until beginning of solenomere, including “postfemoral” part lying beyond lateral sulcus). Femorite slender, slightly curved and not enlarged distad, “postfemoral” part demarcated by an oblique lateral sulcus; tip of solenophore evidently trifid, middle denticle much smaller than both a terminal tooth and a subterminal lobule; solenomere about as long as solenophore, flagelliform.

**Figure 1.**
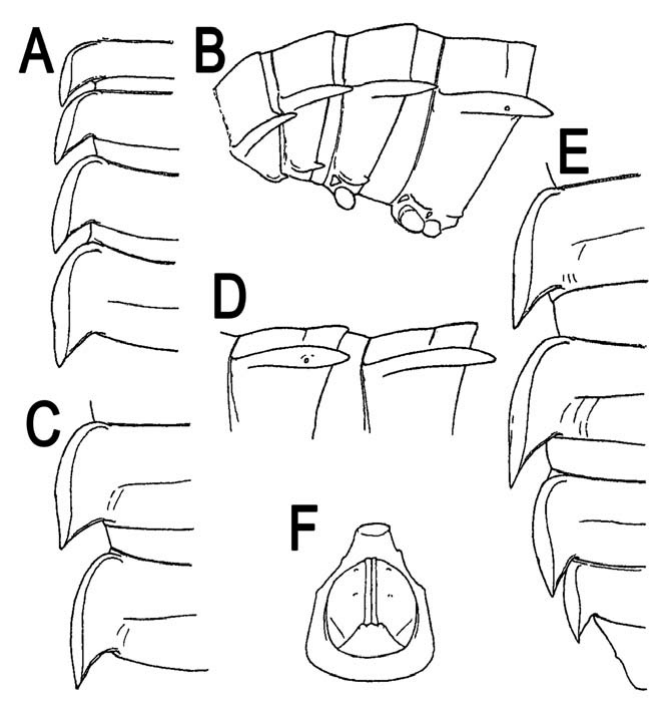
*Orthomorpha beaumontii* (Le Guillou, 1841), ♀ holotype. **A, B** segments 2–5, dorsal and lateral views, respectively **C, D** segments 10 and 11, dorsal and lateral views, respectively **E** segments 16–20, dorsal view **F** posterior part of body, ventral view (after Jeekel 1963).

**Figure 2. F2:**
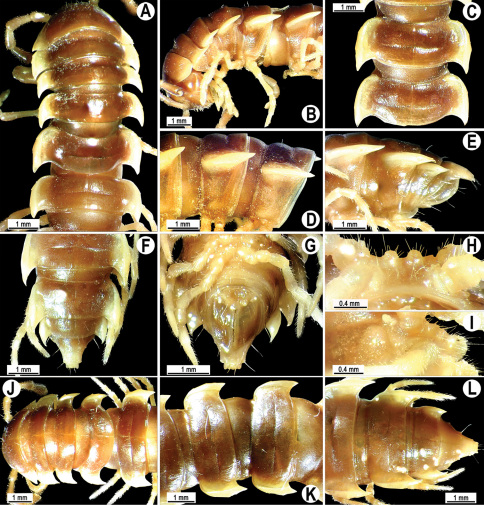
*Orthomorpha beaumontii* (Le Guillou, 1841), ♂ (**A–I**) and ♀ (**J–L**) syntypes of *Orthomorpha hydrobiologica spinala* Attems, 1932. **A, B, J** anterior part of body, dorsal, lateral and dorsal views, respectively **C, D, K** segments 10 and 11, dorsal, lateral and dorsal views, respectively **E–G, L** posterior part of body, lateral, dorsal, ventral and dorsal views, respectively **H, I** sternal cones between coxae 4, subcaudal and sublateral views, respectively.

**Figure 3.**
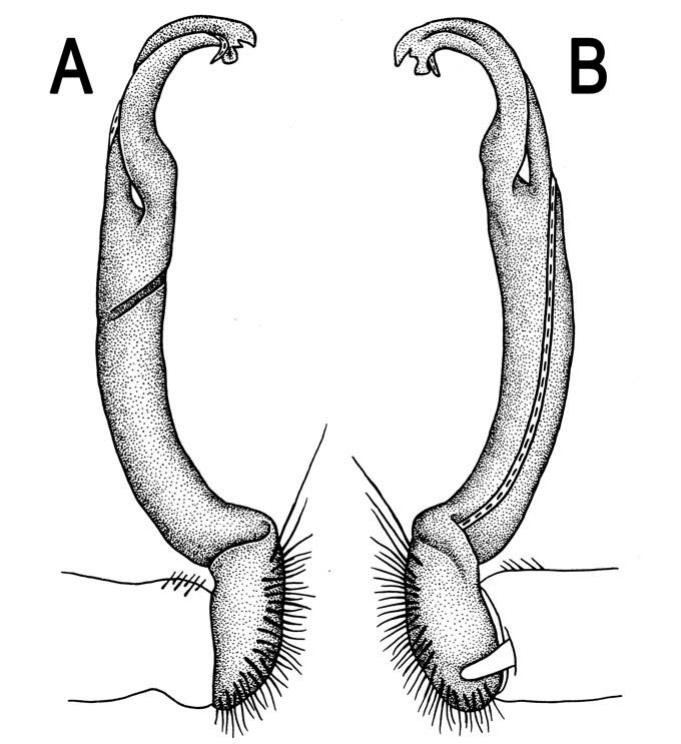
*Orthomorpha beaumontii* (Le Guillou, 1841), ♂ syntype of *Orthomorpha hydrobiologica spinala* Attems, 1932. **A,** **B** right gonopod, lateral and mesal views, respectively.

#### Remarks.

A complete historical review of the typification of *Orthomorpha* has long been provided by Jeekel (1963). Despite some confusion, *Orthomorpha* was properly typified by Pocock (1909), with *Orthomorpha beaumontii* (Le Guillou, 1841) serving as the type-species.

Originally described as a subspecies of *Orthomorpha hydrobiologica* (see Attems 1932), not as a variety as mistakenly quoted by Jeekel (1968), *spinala* has since been treated as a full species (Jeekel 1968). The above samples, especially the only available ♀ syntype, agree in almost every detail with the very accurate redescription of the *Orthomorpha beaumontii* holotype provided by Jeekel (1963), making a restudy of the holotype superfluous. The few differences, such as size (4.4 vs 5.0 mm), coloration (brown vs blackish), the shape of the caudal tooth on pleurosternal carinae (sharp teeth vs triangular lappets), the presence of an anterolateral denticle on paraterga (very small vs virtually missing) etc., are deemed too minor, rather reflecting individual or population-level variation, to consider *Orthomorpha spinala* as being distinct from *Orthomorpha beaumontii* at the species level. Hence the new synonymy advanced. In addition, the type series of *Orthomorpha spinala* derives from an islet lying nearly halfway between Java and the *beaumontii* type locality, Borneo.

Jeekel (1963), when trying to find the closest match among the known *Orthomorpha* species to the holotype of *Orthomorpha beaumontii*, emphasized its especially strong similarities to *Orthomorpha weberi*. Slight differences were only noted in the shape of the paraterga. It was this that allowed Jeekel to unequivocally conserve the concept of *Orthomorpha*. Zoogeographically, the strong morphological similarities between *Orthomorpha beaumontii* and *Orthomorpha weberi* make sense, because the latter species is endemic to Java, Indonesia.

With the above synonymization, the nomenclature of *Orthomorpha* becomes stabilized, confirming this genus’ present scope. The identity of its type-species, *Orthomorpha beaumontii*, has been refined, based on male characters as well.

Bollman (1893), when proposing *Orthomorpha* as a replacement name for the preoccupied *Paradesmus* De Saussure, 1859, synonymized *Orthomorpha beaumontii* with *Orthomorpha spectabilis* (Karsch, 1881), the latter species from Java. Apparently because he provided no evidence whatsoever to substantiate his synonymization, it has since been neglected, *spectabilis* still remaining a dubious name (e.g. Attems 1937; Jeekel 1968).

(Attems (1898, 1914, 1937) referred to *beaumontii* a sample from Java, Indonesia which he had received from the Berlin Museum, thus providing a second record of this species. However, because the gonopod tip of that sample shows a remarkably small subterminal lappet, while the paraterga and pleurosternal carinae slightly differ in shape from those of *beaumontii*, there can be no doubt of Attems’ misidentification. Hence our references to it as such in the catalogue section above.

##### Species with only a single terminal lobule on the gonopod tip

### Orthomorpha coarctata

(De Saussure, 1860)

http://species-id.net/wiki/Orthomorpha_coarctata

**Figure 4.**
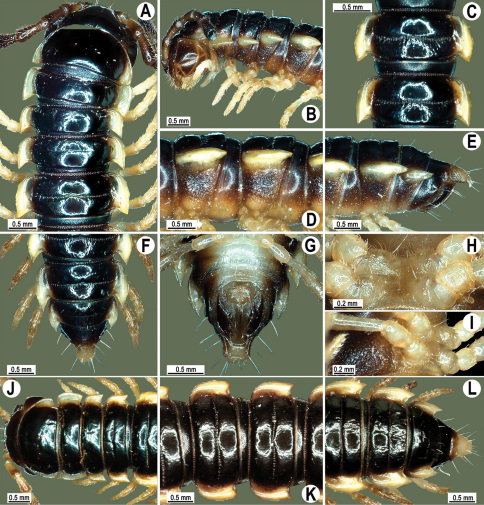
*Orthomorpha coarctata* (De Saussure, 1860), ♂ (**A–I**) and ♀ (**–L**) from Thai Restaurant. **A, B, J** anterior part of body, dorsal, lateral and dorsal views, respectively **C, D, K** segments 10 and 11, dorsal, lateral and dorsal views, respectively **E-G, L** posterior part of body, lateral, dorsal, ventral and dorsal views, respectively **H, I** sternal cones between coxae 4, subcaudal and sublateral views, respectively.

Figs 4 [Fig F5] [Fig F6] [Fig F7] 8

Polydesmus coarctatus De Saussure 1860: 297 (D).
Paradesmus flavocarinatus Daday 1889: 136 (D).
Orthomorpha coarctata – Attems 1937: 62 (D); Jeekel 1968: 45 (M) et auctorum.
Orthomorpha coarctata gigas Attems 1927: 63 (D).
Asiomorpha coarctata – Verhoeff 1939: 117 (D) et auctorum.
Orthomorpha coarctata gigas – Jeekel 1968: 45 (M); Golovatch 1998: 43 (D) et auctorum.

#### Material examined.

1 ♂, 2 ♀(CUMZ), Thailand, Chiang Mai Prov., Mae Rim Distr., Queen Sirikit Botanical Garden, 19°30'17"N, 99°25'89"E, 03.03.2007, leg. N. Likhitrakarn. 1 ♀ (CUMZ), Nan Prov., Mueang Nan Distr., Nan Resort, ca 240 m, 18°54'21"N, 100°45'45"E, 11.10.2009, leg. N. Likhitrakarn. 1 ♂, 1 ♀ (CUMZ), same Prov., Pua Distr., Wora Nakhom Subdistr., ca 270 m, 19°55'43"N, 100°55'14"E, 29.01.2010, leg. R. Chanabun. 1 ♂ (CUMZ), Phrae Prov., Rong Kwang Distr., Hoylong Waterfall, 18°44'39"N, 100°44'95"E, 09.10.2009, leg. R. Chanabun. 1 ♂ (CUMZ), Phitsanulok Prov., Wang Thong Distr., Sukunotayan Waterfall, 17°23'73"N, 100°53'5"E, 09.10.2009, leg. N. Likhitrakarn. 3 ♂ (CUMZ), Tak Prov., Tha Song Yong Distr., Mae Usu Cave, ca 140 m, 17°18'16"N, 98°09'21"E, 30.05.2009, leg. N. Likhitrakarn. 1 ♀ (CUMZ), same Prov., Umphang Distr., Thee Lor Su Riverside Resort, ca 470 m, 16°02'47"N, 98°51'9"E, 06.06.2009, leg. R. Chanabun. 7 ♂, 5 ♀ (CUMZ), Kamphaeng Phet Prov., Mueang Kamphaeng Phet Distr., Thai Restaurant, 17.01.2011, leg. N. Likhitrakarn & R. Chanabun. 1 ♀ (CUMZ), Ubon Ratchathani Prov., Khongchiam Distr., Tadton Waterfall, 14.05.2011, leg. R. Chanabun. 1 ♀ (CUMZ), Saraburi Prov., Chaloem Phra Kiat Distr., Siwilai Cave, 15°12'03"N, 101°27'13"E, 31.09.2006, leg. R. Chanabun. 2 ♂, 1 ♀ (CUMZ), Phra Nakhon Si Ayutthaya Prov., Mueang Phra Nakhon Si Ayutthaya Distr., 15.11.2009, leg. C. Sutcharit. 1 ♂ (CUMZ), same Prov., Maharat Distr., Seafood Restaurant, 20.09.2009, leg. N. Likhitrakarn. 1 ♂ (CUMZ), Nakhon Nayok Prov., Ban Na Distr., near house, 17.05.1952, leg. K. Isarankura. 1 ♂ (CUMZ), Sa Kaeo Prov., Khlong Hat Distr., Thamphet Temple, ca 170 m, 13°21'15"N, 102°18'47"E, 17.09.2009, leg. R. Chanabun. 1 ♂, 1 ♀ (CUMZ), Bangkok Prov., Pattum Wan Distr., Chulalongkorn University campus, 14°11'0"N, 100°53'02"E, 03.07.2009, leg. N. Likhitrakarn. 1 ♂ (CUMZ), Phetchaburi Prov., Ban Laem Distr., 20.07.2009, leg. R. Chanabun. 1 ♂ (CUMZ), Chonburi Prov., Si Racha Distr., Khao Kheow Open Zoo, 13°21'54"N, 101°05'17"E, 03.04.2007, leg. R. Chanabun. 1 ♀ (CUMZ), same Prov., Bothong Distr., Bothong Waterfall, ca 90 m, 13°15'01"N, 101°22'33"E, 15.09.2009, leg. R. Chanabun. 1 ♀ (CUMZ), Chanthaburi Prov., Khlung Distr., Makok Waterfall, 12°59'16"N, 102°26'02"E, 03.09.2007, leg. C. Sutcharit. 1 ♀ (CUMZ), same Prov., Khaeng Hang Maeo Distr., Khao Sibhachan, 13°33'36"N, 102°11'49"E, 30.12.2008, leg. N. Likhitrakarn. 1 ♀ (CUMZ), Ranong Prov., Mueang Ranong Distr., 06.10.2008, leg. C. Sutcharit. 8 ♂, 13 ♀, 3 juv. (CUMZ), Phang Nga Prov., Khura Buri Distr., Similan National Park, Ko Similan, Island 8, 8°40'01"N, 97°38'54"E, 07.04.2010, leg. S. Panha & N. Likhitrakarn. 1 ♂ (CUMZ), Surat Thani Prov., Ban Ta Khun Distr., Ratchaprapa Dam, 9°37'20"N, 99°21'0"E, 08.10.2008, leg. N. Likhitrakarn. 2 ♀ (CUMZ), Phatthalung Prov., Mueang Phatthalung Distr., Tham Malai Thep Nimit, ca 20 m, 7°38'09"N, 100°05'04"E, 11.01.2009, leg. R. Chanabun. 2 ♀ (CUMZ), Satun Prov., Mueang Satun Distr., Tarutao National Park, Ao Talo Wow, 7°02'30"N, 100°08'20"E, 09.04.2008, leg. R. Chanabun. 1 ♀ (CUMZ), Malaysia, Terengganu, Kenyir Lake, ca 170 m, 4°50'44"N, 102°43'15"E, 25.05.2011, leg. R. Chanabun. 7 ♂, 18 ♀ (CUMZ), Johor, Yong Peng, ca 20 m, 2°0'40"N, 103°03'25"E, 21.05.2011, leg. R. Chanabun. Syntypes of *Orthomorpha coarctata gigas*: 1 ♂, 1 ♀ (NHMW-3504), Indonesia, Maluku Prov., Banda Sea, Maluku Barat Regency, Poelau Island, Teoen (= Teun), leg. Kopstein.

#### Descriptive notes.

**Figure 5. F5:**
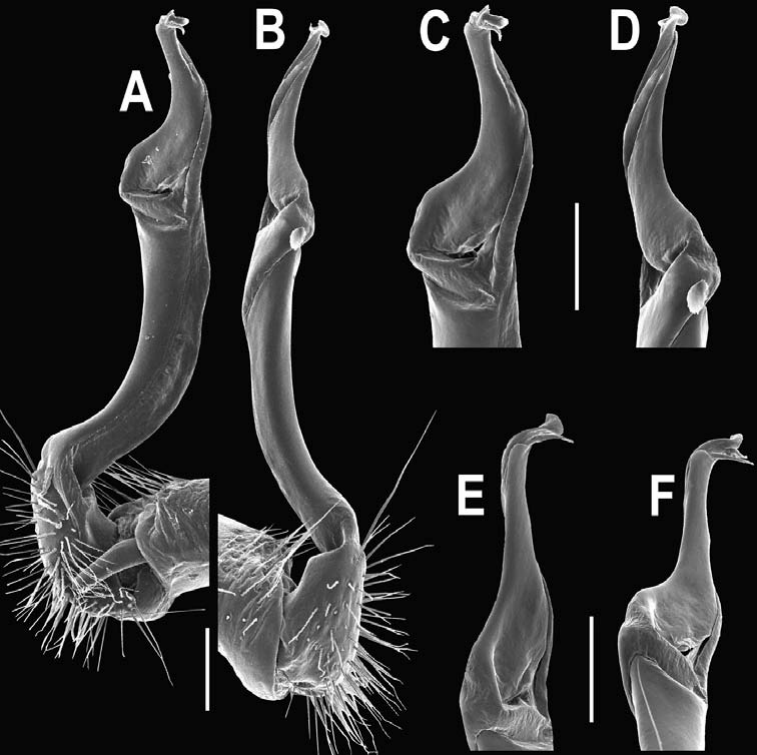
*Orthomorpha coarctata* (De Saussure, 1860), ♂ from Chulalongkorn University campus. **A, B** right gonopod, mesal and lateral views, respectively **C-F** distal part of right gonopod, mesal, lateral, subcaudal and suboral views, respectively. Scale bar: 0.2 mm.

**Figure 6. F6:**
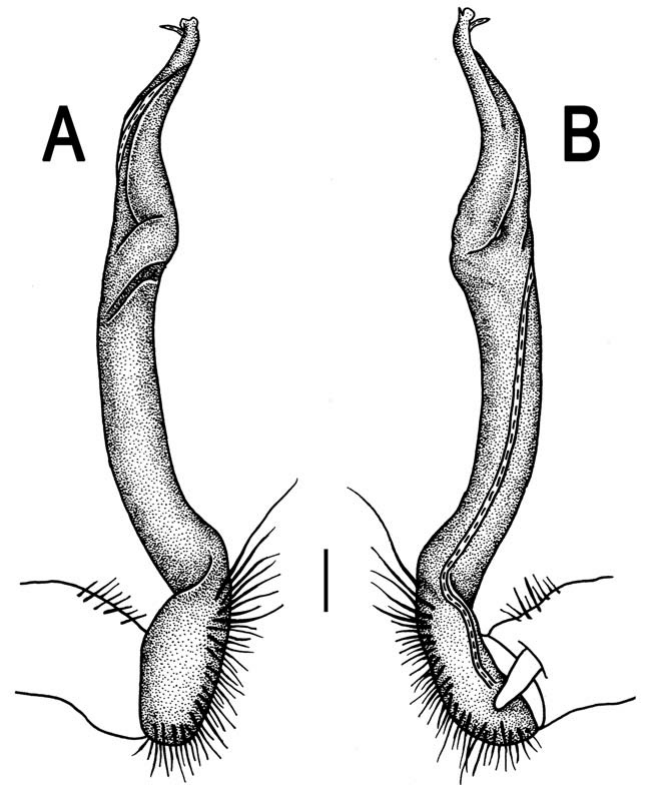
*Orthomorpha coarctata* (De Saussure, 1860), ♂ from Mueang Kamphaeng Phet Distr., Thai Restaurant. **A, B** right gonopod, lateral and mesal views, respectively.Scale bar: 0.2 mm.

**Figure 7. F7:**
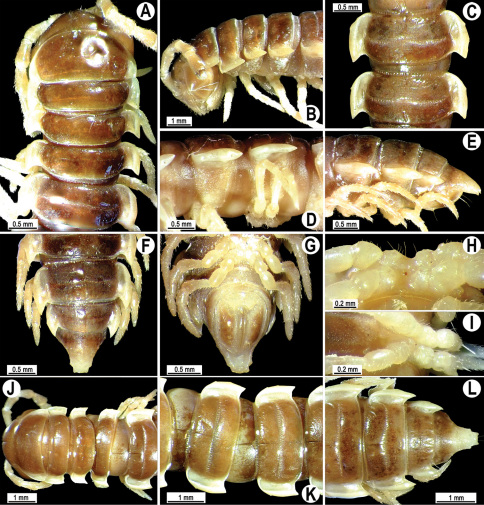
*Orthomorpha coarctata* (De Saussure, 1860), ♂ (**A–I**) and ♀ (**J–L**) syntype of *Orthomorpha coarctata gigas* Attems, 1927. **A, B, J** anterior part of body, dorsal, lateral and dorsal views, respectively **C, D, K** segments 10 and 11, dorsal, lateral and dorsal views, respectively **E–G, L** posterior part of body, lateral, dorsal, ventral and dorsal views, respectively **H, I** sternal cones between coxae 4, subcaudal and sublateral views, respectively.

**Figure 8.**
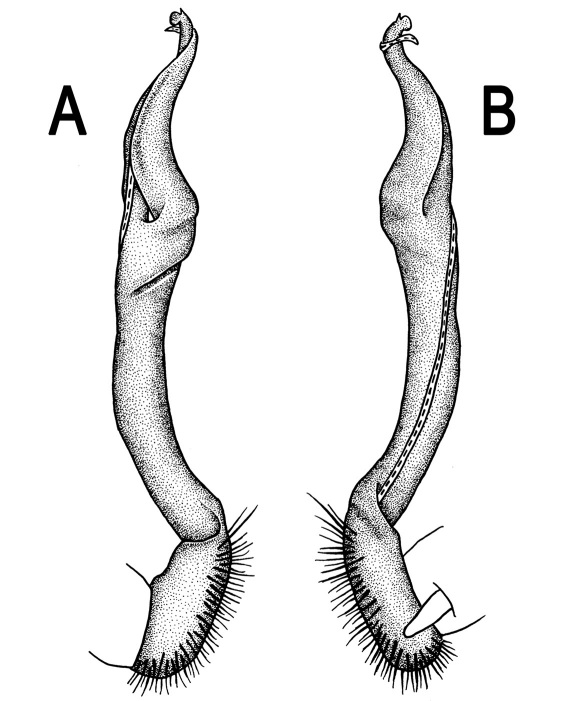
*Orthomorpha coarctata* (De Saussure, 1860), ♂ syntype of *Orthomorpha coarctata gigas* Attems, 1927. **A, B** right gonopod, lateral and mesal views, respectively.

Length 14.5–20.5 (♂) or 16.5–27.5 mm (♀), width of midbody pro- and metazona 1.1–1.7 and 1.5–2.7 mm (♂), 1.1–2.5 and 1.6–3.2 mm (♀), respectively.Coloration, texture, all main somatic and gonopod characters as in Figs 4–8. Neither sternal lobe nor cone(s) between ♂ coxae 4 (Fig. 4H & I), at most only traces of poor knobs (Fig. 7H & I).

Gonopods (Figs 5, 6 & 8) with tip of solenophore produced into a single lobe, all other spikes or denticles being either missing or nearly missing.

#### Remarks.

This pantropical species has been described and redescribed several times, often under different names (e.g. Jeekel 1968). From Thailand, it had been described as *Paradesmus flavocarinatus* until Enghoff (2005) showed it to actually represent still another synonym of *Orthomorpha coarctata*. No full catalogue is attempted here as being superfluous, with hundreds of references involved.

Our restudy of the type material of *Orthomorpha coarctata gigas*, kept in NHMW (Figs 7 & 8), confirms Jeekel’s (1968) synonymization of this subspecies with *Orthomorpha coarctata* s. str.

##### Species with a bifid gonopod tip, with only a terminal and a subterminal denticle or lobule

### Orthomorpha arboricola

(Attems, 1937)

http://species-id.net/wiki/Orthomorpha_arboricola

Figs 9 10

Pratinus arboricola Attems 1937: 120 (D).
Pratinus arboricola – Attems 1938: 222 (D).
Orthomorpha arboricola – Jeekel 1963: 265 (M); 1964: 361 (M, D); 1968: 56 (M); Enghoff et al. 2004: 38 (M); Golovatch 1998: 42 (D).

#### Lectotype.

♂ (NHMW-3500), Vietnam, Lamdong Prov., Dalat, 1500 m, 31.01.1935, leg. C. Dawydoff.

#### Paralectotypes.

2 ♂, 1 ♀, 10 juv. (NHMW-3500), same locality, together with lectotype. Numerous fragments (NHMW-3499), Vietnam, Lamdong Prov., Peak Lang Biang, Arbre-Broyé, 1400 m, 02.02.1931, leg. C. Dawydoff.

Lectotype designation proposed herewith is necessary to ensure the species is based on a complete male.

#### Redescription.

**Figure 9.**
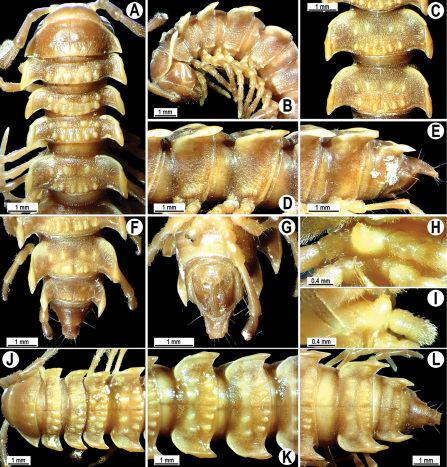
*Orthomorpha arboricola* (Attems, 1937), ♂ lectotype (**A–I**) and ♀ paralectotype (**J–L**). **A, B, J** anterior part of body, dorsal, lateral and dorsal views, respectively **C, D, K** segments 10 and 11, dorsal, lateral and dorsal views, respectively **E–G, L** posterior part of body, lateral, dorsal, ventral and dorsal views, respectively **H, I** sternal cones between coxae 4, subcaudal and sublateral views, respectively.

Length 28–31 mm (♂), 34 mm (♀), width of midbody pro- and metazona 2.3–2.8 and 3.8–4.1 mm (♂), 3.4 and 4.6 mm (♀), respectively (vs up to 38 mm length, as given in the original descriptions (Attems 1937, 1938)). Coloration of alcohol material upon long-term preservation rather uniformly light brown to brown (Fig. 9) (vs light red-brown to dark brown, as given in the original descriptions (Attems 1937, 1938)).

Head usual, clypeolabral region sparsely setose (vs densely setose, as given in the original descriptions (Attems 1937, 1938)), surface of vertex smooth; epicranial suture distinct, flanked by rugulose patches. Antennae long and slender (Fig. 9A), extending behind until body segment 4 (♂, ♀) dorsally. Head in width < collum < segments 3 and 4 < segment 2 < segments 5–16, gently and gradually tapering thereafter. Collum with three transverse rows of setae, 4+4 anterior, 2+2 intermediate, and 3+3 posterior setae; caudal corner of paraterga dentiform, pointed, directed caually; 3+3 small tubercles in front of caudal margin (Fig. 9A & J). Tegument dull, prozona very finely shagreened, metaterga finely rugulose and microgranulate, surface below paraterga slightly more so. Postcollum metaterga with two transverse rows of setae, these being always borne on low, oblong, rounded tubercles: 2+2 in anterior (pre-sulcus) row, 3(4)+3(4) in posterior (postsulcus) one. Axial line visible both on pro- and metazona. Paraterga very strongly developed (Fig. 9A-G, J-L), especially so in ♂, subhorizontal to slightly upturned, lying level to or slightly above dorsum, thin in lateral view, like blunt blades, a little thicker only on pore-bearing segments, on postcollum segments always clearly projecting well behind tergal margin. Calluses delimited only dorsally, thin, especially so on poreless segments. Paraterga 2 broad, anterior edge angular, lateral edge with one larger and two smaller, but evident incisions in anterior 1/3; posterior edge evidently concave (Fig. 9A & J). Paraterga 3 and 4 subequal, like subsequent paraterga, anterior edge broadly rounded, bordered and fused to callus, lateral edge with a small incision in anterior third. Paraterga 16–19 with tip of caudal corner slightly curved mesad. Ozopores evident, lateral, lying in an ovoid groove at about 1/3 of metazonital length. Transverse sulcus present on metaterga 5–18, shallow, reaching bases of paraterga, smooth at bottom, slightly sinuate anteromedially (Fig. 9A, C, F, J-L). Stricture between pro- and metazona rather narrow, shallow, beaded at bottom down to base of paraterga. Pleurosternal carinae complete crests only on segments 2–7 (♂) (Fig. 9B & D) or 2–4 (♀), each with an evident sharp denticle caudally, thereafter increasingly reduced until segment 11 (♂, ♀). Epiproct (Fig. 9E, F & L) conical, flattened dorsoventrally, apical papillae small; tip subtruncate; preapical papillae small, but visible. Hypoproct (Fig. 9G) semi-circular, setiferous knobs at caudal edge well-separated.

Sterna sparsely setose, without modifications, but with a very evident, high, setose, central cone between ♂ coxae 4 (Fig. 9H & I). A conspicuous ridge in front of gonopod aperture. Legs long and slender, only slightly incrassate in ♂, midbody ones ca 1.4–1.5 (♂) or 1.1–1.2 times (♀) as long as body height, prefemora without modifications, tarsal brushes present until legs of segment 10.

**Figure 10.**
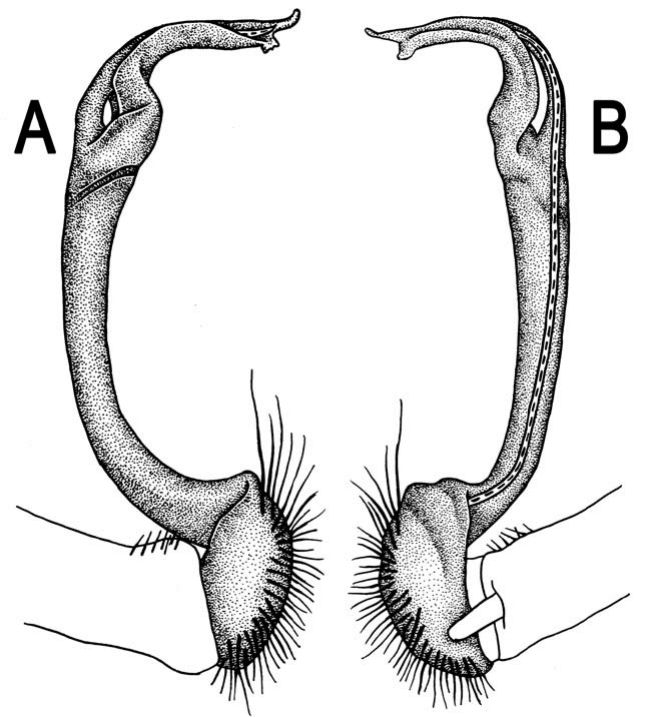
*Orthomorpha arboricola* (Attems, 1937), ♂ lectotype. **A, B** right gonopod lateral and mesal views, respectively.

Gonopods (Fig. 10) simple. Coxa long and slender, with several setae distodorsally. Prefemur densely setose, more than 2 times shorter than femorite (measured until beginning of solenomere). Femorite slender, evidently curved and slightly enlarged distad, “postfemoral” part demarcated by an oblique lateral sulcus; tip of solenophore evidently bifid, subterminal lobule with three minute denticles at distal margin.

#### Remarks.

This species is only known from southern Vietnam (Dalat and Peak Lang Biang).

### Orthomorpha picturata sp. n.

urn:lsid:zoobank.org:act:5D8B4005-5BAE-41F9-8718-C7FD517A1838

http://species-id.net/wiki/Orthomorpha_picturata

Figs 11 12

#### Holotype.

♂ (CUMZ), Thailand, Phang Nga Prov., Khura Buri Distr., Similan National Park, Ko Bangu, Island 9, ca 40 m, 8°40'33"N, 97°37'08"E, 06.04.2010, leg. N. Likhitrakarn.

#### Paratypes.

2 ♀ (CUMZ), same locality, together with holotype.

#### Name.

To emphasize the picturesque appearance of the animals.

#### Diagnosis.

The new species differs in the small size (up to 23 mm long and 3.5 mm wide), coupled with a particular coloration and a bifid gonopod tip (see also Key below).

#### Description.

**Figure 11.**
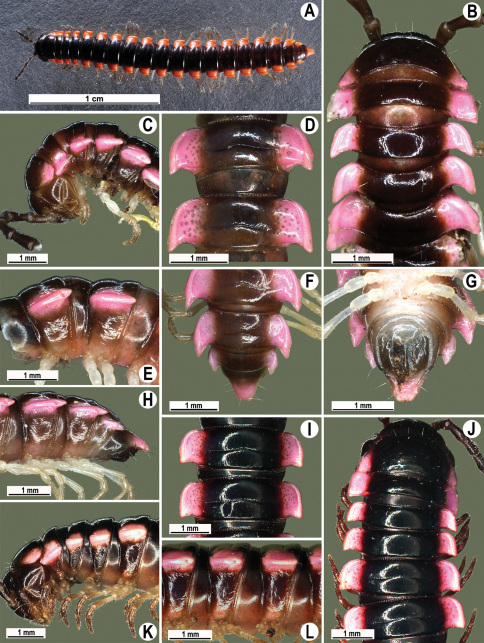
*Orthomorpha picturata* sp. n., ♂ holotype (**B–H**) and ♀ paratype (**A, I–L**). **A** habitus, live coloration **B, C, J, K** anterior part of body, dorsal, lateral, dorsal and lateral views, respectively **D, E, I, J** segments 10 and 11, dorsal, lateral, dorsal and lateral views, respectively **F-H** posterior part of body, dorsal, ventral and lateral views, respectively.

Length ca 19 mm (♂) and 22–23 mm (♀), width of midbody pro- and metazona 1.8 and 2.7 mm (♂), 2.3–2.5 and 3.4–3.5 mm (♀), respectively. Coloration of live animals blackish, paraterga and epiproct contrasting creamy orange, legs and venter brownish to pale brown; coloration of alcohol material after preservation faded to castaneous brown or pale brown, paraterga (marbled at base) and epiproct somewhat faded to pale pinkish or pale yellow, legs and venter paler brown (Fig. 11A-J).

Clypeolabral region densely setose, vertex sparsely setose, epicranial suture distinct. Antennae moderately long, clavate (antennomere 7 broad) (Fig. 11A & C), extending behind until body segment 3 (♂) or beyond segment 2 (♀) dorsally.

Head in width < collum < segments 3–4 < 2 = 5–16 (♂, ♀); thereafter body gently and gradually tapering. Collum with three transverse rows of setae, 4+4 anterior, 2+2 intermediate, and 2+2 posterior; caudal corner of paraterga very narrowly rounded, slightly bordered and declined ventrally, not extending behind tergal margin (Fig. 11B & J). Tegument smooth and shining, prozona very finely shagreened, metazona leathery, faintly rugulose, below paraterga more evidently so. Postcollum metaterga with a transverse anterior row of 2+2 setae. Tergal setae long, slender, about 1/3 metatergal length. Axial line faint, barely traceable on metaterga. Paraterga very strongly developed (Fig. 11A-J), especially well so in ♂, slightly upturned and lying below dorsum (at about 1/3 on body height), except for paraterga 2 being subhorizontal, broad in dorsal aspect and thin in lateral view; shoulders well-developed, slightly rounded and oblique laterally; caudal tip of paraterga 2 nearly pointed, increasingly well pointed on paraterga 14–19; paraterga bent posteriad, at least slightly extending behind tergal margin, more evidently so on segments 2–3 and 14–19. Calluses delimited by a sulcus only dorsally, rather narrow, a little broader on pore-bearing segments, with 1–2 small lateral incisions in anterior 1/4 and 3/4 on callus 2 and following pore-bearing segments, but only one (at front 1/4) on following poreless segments (Fig. 11C, E, H, K & I). Posterior edge of paraterga evidently concave, more strongly so on segments 16–19 (Fig. 11F-H). Ozopores evident, lateral, lying in an ovoid groove at about 1/4 paratergal length in front of caudal corner. Transverse sulcus present on metaterga 5–18, usually narrow and shallow (Fig. 11B, D & F), superficial (especially so due to coarse texture around), slightly not reaching bases of paraterga, a little better developed in ♀ (Fig. 11I & J). Stricture between pro- and metazona broad, evidently ribbed at bottom down to base of paraterga (Fig. 11B, D, E & H). Pleurosternal carinae complete crests bulged anteriorly and with a sharp caudal tooth on segments 2–7, thereafter only a sharp caudal tooth on segments 8–18, a very small denticle on segment 19 (Fig. 11C, E & H), or crests bulged anteriorly and with a sharp caudal tooth on segments 2–4, thereafter only a small sharp caudal tooth on segments 5–16 (♀). Epiproct (Fig. 11F-H) conical, flattened dorsoventrally, with two evident apical papillae, these especially clear in ♂, slightly concave at tip; pre-apical papillae evident. Hypoproct (Fig. 11G) semi-circular, setiferous knobs at caudal edge well-separated.

Sterna delicately and sparsely setose, without modifications; a paramedian pair of small, strongly separated, setose tubercles between ♂ coxae 4. Gonopod aperture broken during removal of gonopods. Legs moderately long and slender, slightly incrassate in ♂, midbody ones ca 1.2–1.4 (♂) or 0.9–1.1 times (♀) as long as body height, prefemora without modifications, ♂ tarsal brushes present until legs of segment 8.

**Figure 12.**
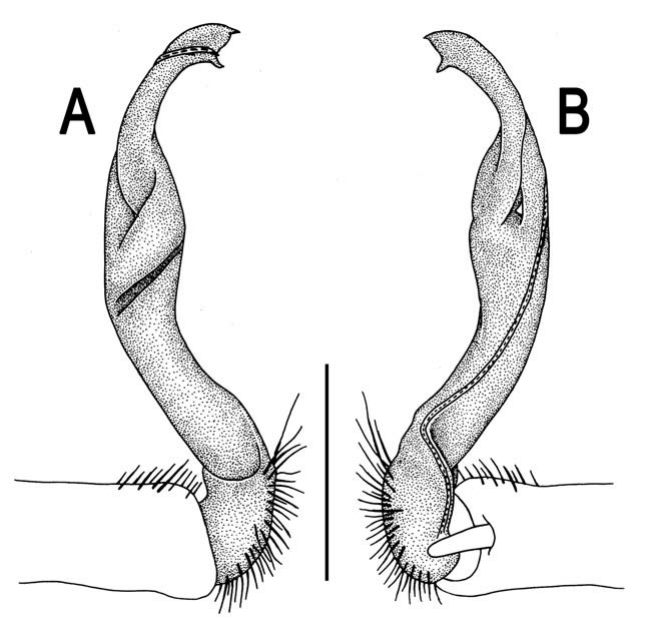
*Orthomorpha picturata* sp. n., ♂ holotype. **A, B** right gonopod, lateral and mesal views, respectively. Scale bar: 0.5 mm.

Gonopods (Fig. 12) simple. Coxa long and slender, with several strong setae distodorsally. Prefemoral part densely setose, less than half the length of femorite + ”postfemoral” part. The latter slender, slightly curved and not enlarged distad, with a “postfemoral” part demarcated by an oblique lateral sulcus. Solenophore with a bidentate tip, both prongs being subequal; solenomere long and flagelliform.

#### Remarks.

**Map 2.**
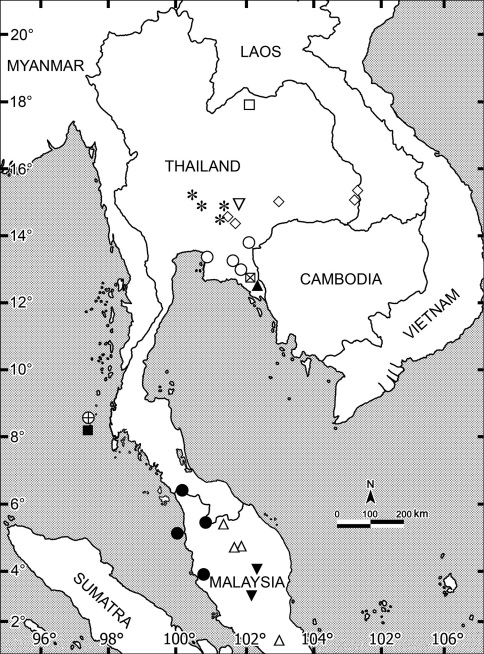
Distributions of the new species of *Orthomorpha*. Crossed circle: *Orthomorpha picturata* sp. n.; Asterisk: *Orthomorpha tuberculifera* sp. n.; Inverted open triangle: *Orthomorpha subtuberculifera* sp. n.; Open diamond: *Orthomorpha communis* sp. n.; Crossed square: *Orthomorpha atypica* sp. n.; Filled triangle: *Orthomorpha latiterga* sp. n.; Open square: *Orthomorpha suberecta* sp. n.; Open circle: *Orthomorpha isarankurai* sp. n.; Open triangle: *Orthomorpha elevata* sp. n.; Filled circle: *Orthomorpha subelevata* sp. n.; Filled square: *Orthomorpha similanensis* sp. n.; Inverted filled triangle: *Orthomorpha spiniformis* sp. n.

Among all nine islets of the Similan Archipelago, Thailand inspected for millipedes in April 2010, only three appeared to support *Orthomorpha* species, one each per islet. Only *Orthomorpha picturata*occurred on Ko Bangu, Island 9 (Map 2), another new species (*Orthomorpha similanensis* sp. n., see below) on Ko Miang, Island 4, while Ko Huyong, Island 1 harboured still another presumed congener which regrettably cannot be described, because we only obtained female material.

### Orthomorpha tuberculifera sp. n.

urn:lsid:zoobank.org:act:323ACC16-597D-4A54-AADD-903141FD4E54

http://species-id.net/wiki/Orthomorpha_tuberculifera

**Figure 13.**
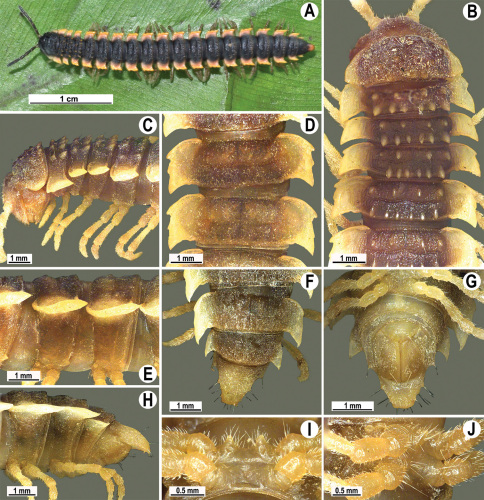
*Orthomorpha tuberculifera* sp. n., ♂ holotype. **A** habitus, live coloration. **B, C** anterior part of body, dorsal and lateral views, respectively **D, E** segments 10 and 11, dorsal and lateral views, respectively **F, G, H** posterior part of body, dorsal, ventral and lateral views, respectively **I, J** sternal cones between coxae 4, subcaudal and sublateral views, respectively.

Figs 13 [Fig F14] 15

#### Holotype.

♂ (CUMZ), Thailand, Nakhon Ratchasima Prov., Pakchong Distr., Khao Rup Chang, ca 420 m, 14°31'33"N, 101°21'36"E, 26.04.2009, leg. S. Panha, R. Chanabun & N. Likhitrakarn.

#### Paratypes.

2 ♂, 2 ♀ (ZMUC), 2 ♂, 2 ♀ (ZMUM), 14 ♂, 21 ♀ (CUMZ), same data as holotype. 1 ♂ (CUMZ), same Distr., Klang Dong Restaurant, ca 360 m, 14°09'10"N, 101°18'35"E, 01.04.2011, leg. R. Chanabun.

#### Name.

To emphasize the evident metatergal tuberculation.

#### Diagnosis.

The new species differs in evidently tuberculated metaterga, coupled with only a small anterolateral incision on paraterga and a bifid gonopod tip (see also Key below).

#### Description.

Length 24–32 mm (♂), 27–34 mm (♀), width of midbody pro- and metazona 2.6–3.3 and 4.5–5.2 mm (♂), 2.9–4.0 and 4.5–5.5 mm (♀), respectively Coloration of live animals blackish, paraterga and epiproct contrasting creamy orange, legs and venter brownish to pale brown (Fig. 13A); coloration of alcohol material after preservation faded to dark castaneous brown, paraterga (marbled at base), metatergal tubercles, middle region of prozona, venter, epiproct, and several basal podomeres more flavous, pale pinkish, brownish or pale yellow (Fig. 13B-J).

Clypeolabral region densely setose, vertex bare, epicranial suture distinct. Antennae short, poorly clavate (Fig. 13A), extending behind segment 2 (♂) or until midway of segment 2 (♀) dorsally.

Head in width << collum < segments 3–4 < 2 = 5–16 (♂), or head < collum < segments 3–4 < 2 < 5–16 (♀); thereafter body gently and gradually tapering. Collum with three transverse rows of medium-sized setae, 4+4 anterior, 2+2 intermediate, and 2+2 posterior, all borne on very evident tubercle, these being especially high in caudal row; paraterga slightly declivous, broadly rounded and narrowly bordered, caudal corner a minute knob, not extending behind tergal margin (Fig. 13B & C). Tegument rather poorly shining, prozona very finely shagreened, metaterga coriaceous, roughly rugose and granulate, below paraterga rugulose. Postcollum metaterga with two transverse rows of short, mostly abraded setae borne on evident tubercles: 2+2 in front (pre-sulcus) row on smaller tubercles and 3+3 in caudal (postsulcus) row on higher, sharper and dorsocaudally inclined tubercles (Fig. 13B-F). Metatergal tubercles especially high on collum and several following segments, growing increasingly lower towards segment 19. Axial line clear, especially so on metaterga. Paraterga very strongly developed (Fig. 13A-H), especially well so in ♂, mostly subhorizontal to faintly declivous, always lying below dorsum, set at about 1/3–1/4 midbody height; shoulders well-developed, mostly straight; caudal tips of paraterga nearly pointed to pointed, always extending behind tergal margin, increasingly well pointed and often bent mesad on paraterga (14-)19. Calluses delimited by a sulcus both dorsally and ventrally, especially deeply so dorsally, rather broad, with three more or less evident lateral incisions on callus 2 and two strong indentations on following segments (Fig. 13B, D & F). Posterior edge of paraterga always evidently concave, more strongly so on segments 16–19 (Fig. 13F). Ozopores evident, lateral, lying in a deep ovoid groove at about 1/3–1/4 paratergal length in front of caudal corner. Transverse sulcus present on metaterga 2–18, incomplete on metaterga 2–4, complete and reaching bases of paraterga on following segments, beaded at bottom, deep (Fig. 13B, D & F). Stricture between pro- and metazona deep, evidently ribbed at bottom down to base of paraterga. Pleurosternal carinae large, roughly granulate crests with a distinct tooth both frontally and caudally, complete on segments 2–9 (♂) or 2–8 (♀), thereafter split into both front and caudal teeth, the former turning into bulges until segment 13(14), the latter tooth gradually reduced until segment 18 (♂) or 17 (♀), much more strongly developed in ♂ than in ♀ (Fig. 13C, E & H). Epiproct (Fig. 13F & G) conical, rather short, flattened dorsoventrally, with two very evident (♂) or rather small, sharp, apical teeth (♀) directed ventrocaudally; pre-apical papillae evident, located close to tip. Hypoproct (Fig. 13G) roundly subtriangular, setiferous knobs at caudal edge clear and well-separated.

Sterna delicately and rather densely setose; a paramedian pair of evident, fully separated, setose cones between ♂ coxae 4 (Fig. 13I & J). A paramedian pair of small tubercles in front of gonopod aperture. Legs moderately long and slender, almost not incrassate in ♂, midbody ones ca 1.2–1.3 (♂) or 0.8–0.9 times (♀) as long as body height, prefemora without modifications, ♂ tarsal brushes present only on ♂ legs 1–3, thereafter gradually thinning out.

**Figure 14. F14:**
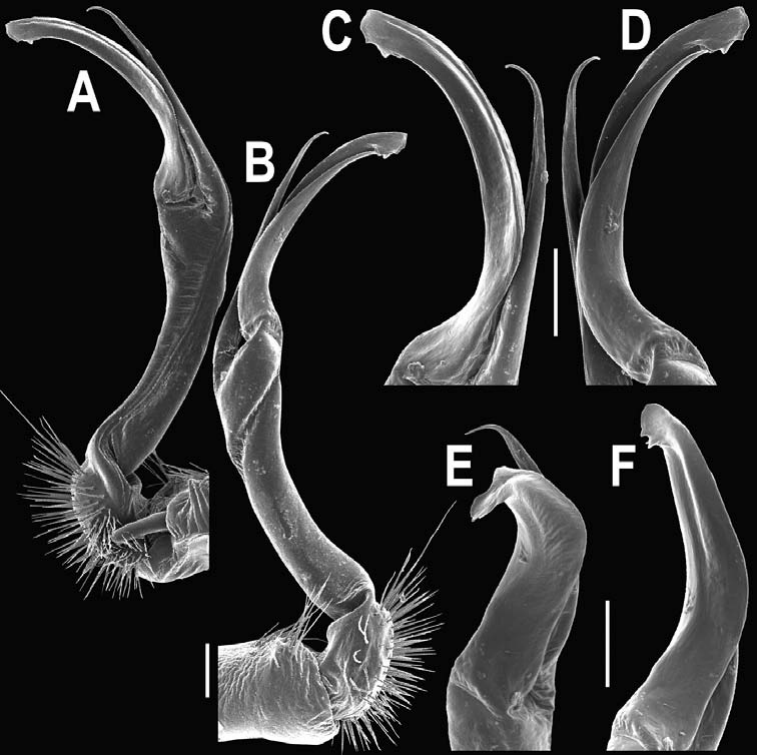
*Orthomorpha tuberculifera* sp. n., ♂ paratype from Khao Rup Chang. **A, B** right gonopod, mesal and lateral views, respectively **C-F** distal part of right gonopod, mesal, lateral, suboral and subcaudal views, respectively. Scale bar: 0.2 mm.

**Figure 15.**
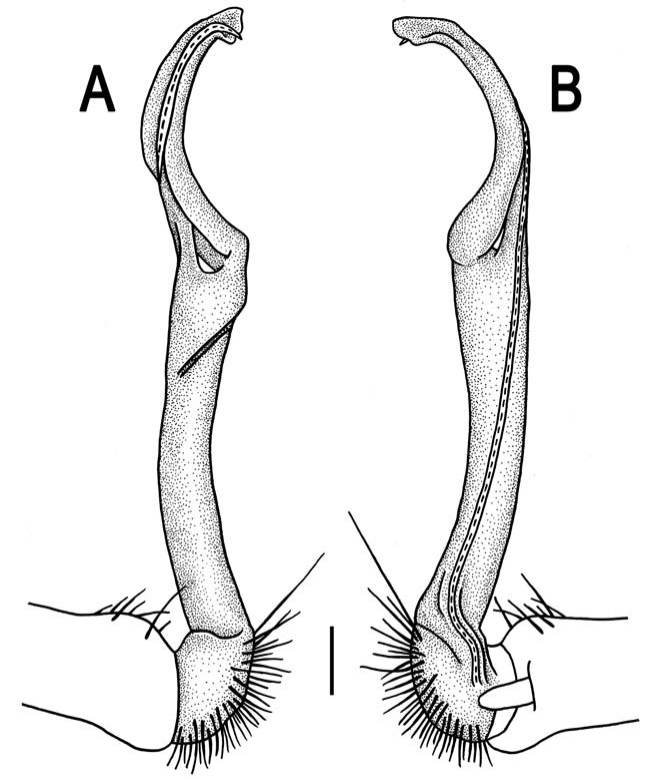
*Orthomorpha tuberculifera* sp. n., ♂ holotype. **A, B** right gonopod lateral and mesal views, respectively. Scale bar: 0.2 mm.

Gonopods (Figs 14 & 15) simple. Coxa long and slender, with several strong setae distodorsally. Prefemoral part densely setose, about 3 times shorter than femorite + “postfemoral” part. Femorite slender, suberect to slightly curved, nearly not enlarged distad, with a “postfemoral” part demarcated by an oblique lateral sulcus. Solenophore tip either very faintly bidentate, with both denticles being subequal, or a nearly smooth lobe; solenomere long and flagelliform.

#### Remarks.

There are two further populations which we regard as representing the same new species. One of the populations (15 ♂, 3 ♀ (CUZM), non-types) comes from Thailand, Lop Buri Prov., Khok Samrong Distr., Khao Wong Phrachan Temple, 14°58'0"N, 100°41'49"E, 07.06.2008, leg. C. Sutcharit. The other one (1 ♂, 2 ♀ (CUZM), non-types) comes from Thailand, Saraburi Prov., Kaeng Khoi Distr., Kaeng Khoi, 14°58'N, 100°59'E, 08.09.2007, leg. S. Panha. All three localities are situated about 100–200 km from one another, west, north and northwest of the Sankamphaeng Mountain Range, Khao Yai National Park within the same hilly area (Map 2).

### Orthomorpha tuberculifera sp. n., variety from Khao Wong Phrachan Temple

**Figure 16.**
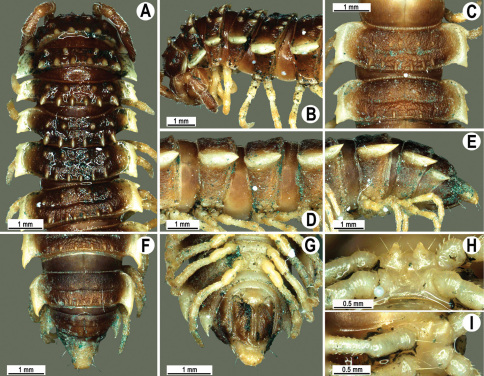
*Orthomorpha tuberculifera* sp. n., ♂, variety from Khao Wong Phrachan Temple. **A, B** anterior part of body, dorsal and lateral views, respectively **C, D** segments 10 and 11, dorsal and lateral views, respectively **E-G** posterior part of body, lateral, dorsal and ventral views, respectively **H, I** sternal cones between coxae 4, subcaudal and sublateral views, respectively.

Figs 16 [Fig F17] 18

#### Description.

Length 22–30 mm (♂), 24–31 mm (♀), width of midbody pro- and metazona 2.6–2.9 and 3.9–4.2 mm (♂), 2.8–3.3 and 4.0–4.3 mm (♀), respectively. Colour pattern same as in the type series of *Orthomorpha tuberculifera* sp. n., but coloration of alcohol material darker, blackish.

**Figure 17. F17:**
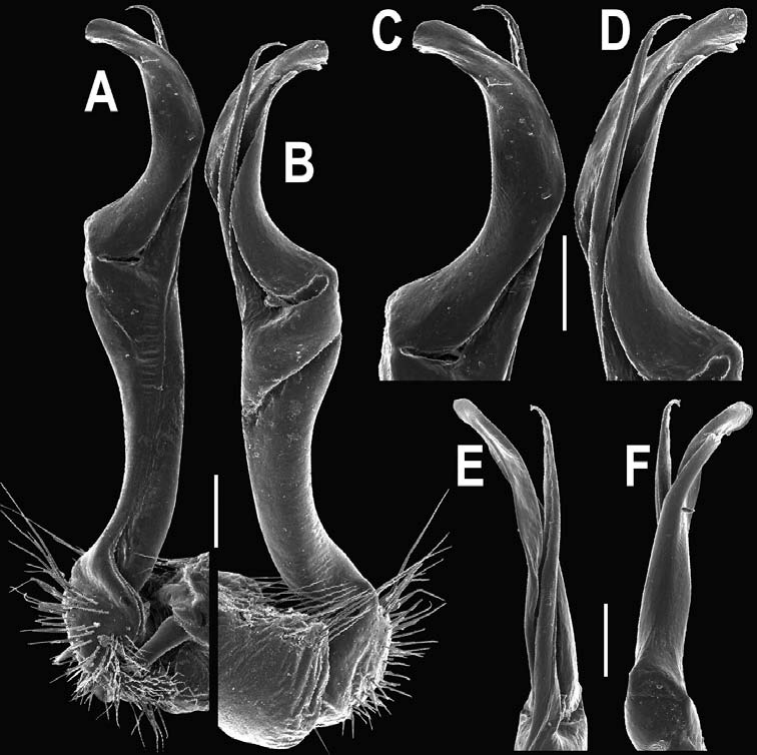
*Orthomorpha tuberculifera* sp. n., ♂, variety from Khao Wong Phrachan Temple. **A, B** right gonopod, mesal and lateral views, respectively **C-F** distal part of right gonopod, mesal, lateral, subcaudal and suboral views, respectively. Scale bar: 0.2 mm.

**Figure 18.**
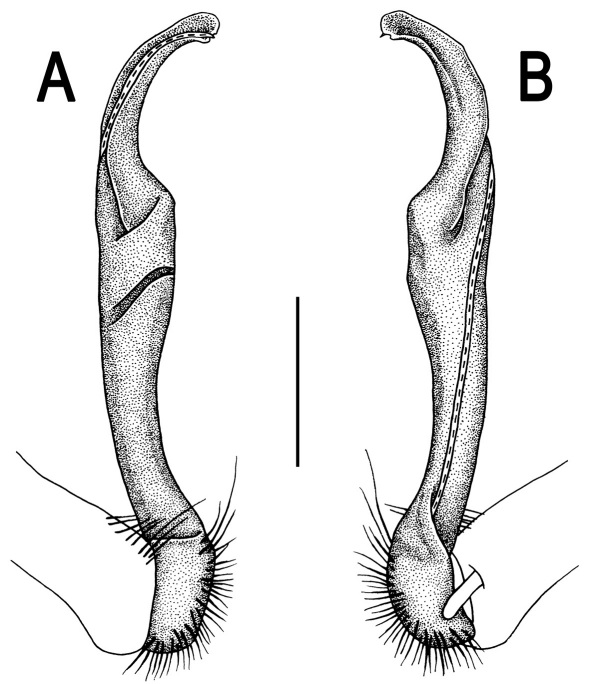
*Orthomorpha tuberculifera* sp. n., ♂, variety from Khao Wong Phrachan Temple. **A, B** right gonopod, lateral and mesal views, respectively. Scale bar: 0.5 mm.

All other characters as in the typical *Orthomorpha tuberculifera* sp. n. (Figs 16–18), except as follows.

Venter and legs sometimes as pallid as paraterga and epiproct. Paraterga slightly less prominent, narrower (Fig. 16A, C & F). Pattern of setigerous tubercles in caudal row on metatergum 17 sometimes as 4+3, on metatergum 18 as 4+4. Paraterga 2 sometimes with four small indentations on one side, with three ones on the other. Surface below paraterga microgranulate and faintly rugulose.

### Orthomorpha tuberculifera sp. n., variety from Kaeng Khoi

**Figure 19.**
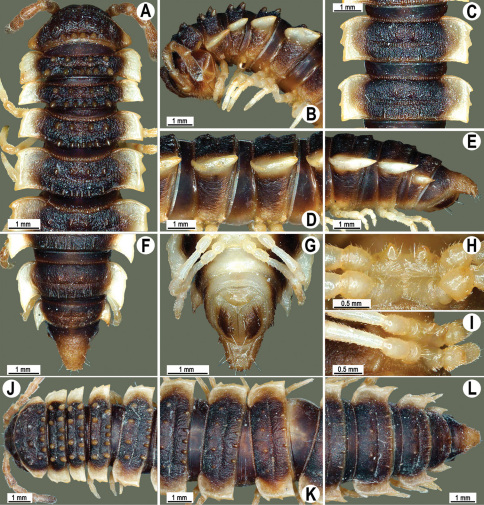
*Orthomorpha tuberculifera* sp. n., ♂, variety from Kaeng Khoi (**A–I**) and ♀ (**J–L**). **A, B, J** anterior part of body, dorsal, lateral and dorsal views, respectively **C, D, K** segments 10 and 11, dorsal, lateral and dorsal views, respectively **E–G, L** posterior part of body, lateral, dorsal, ventral, and dorsal views, respectively **H, I** sternal cones between coxae 4, subcaudal and sublateral views, respectively.

Figs 19 [Fig F20] 21

#### Description.

Length 36 mm (♂), 36–37 mm (♀), width of midbody pro- and metazona 3.0 and 4.9 mm (♂), 3.5–3.7 and 5.2–5.5 mm (♀), respectively. Colour pattern same as in the type series of *Orthomorpha tuberculifera* sp. n., but coloration of alcohol material darker, blackish.

**Figure 20. F20:**
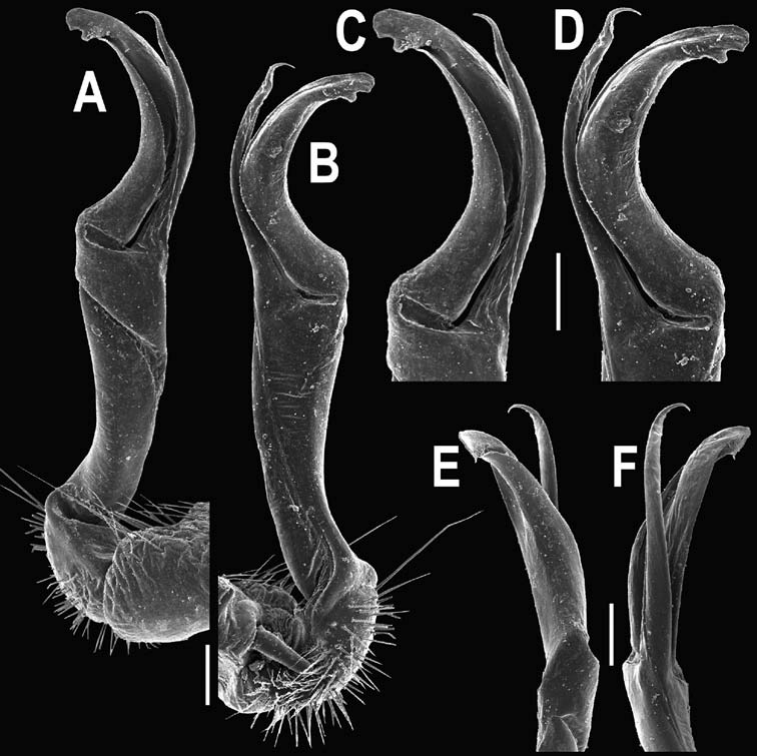
*Orthomorpha tuberculifera* sp. n., ♂ variety from Kaeng Khoi. **A, B** right gonopod, lateral and mesal views, respectively **C**-**F** distal part of right gonopod, lateral, mesal, suboral and subcaudal views, respectively. Scale bar: 0.2 mm.

**Figure 21.**
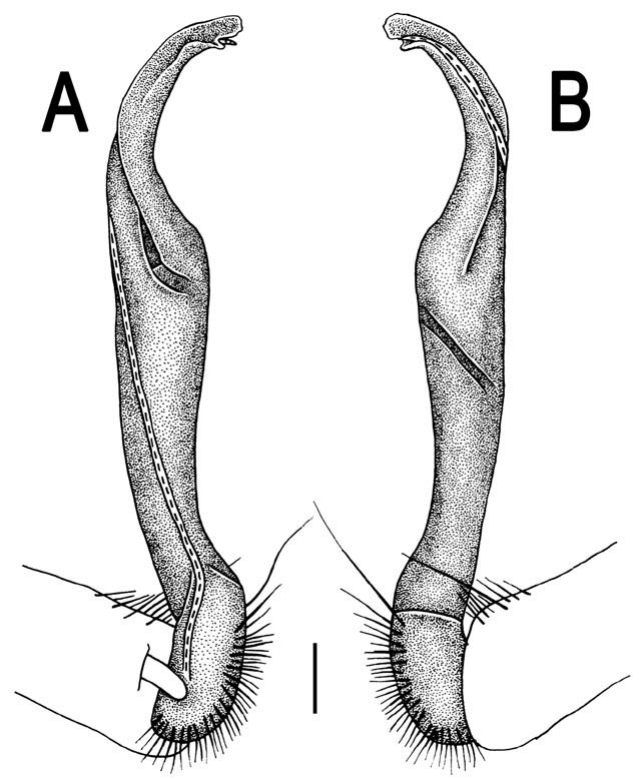
*Orthomorpha tuberculifera* sp. n., ♂ variety from Kaeng Khoi. **A, B** left gonopod, mesal and lateral views, respectively. Scale bar: 0.2 mm.

All other characters as in the typical *Orthomorpha tuberculifera* sp. n. (Figs 19–21), except as follows.

Paraterga less prominent, even midbody ones nearly not produced behind tergal margin (Fig. 19A-E) (Figs 1K–M). Pattern of setigerous tubercles in caudal row on metatergum 19 sometimes as 4+3.

### Orthomorpha subtuberculifera sp. n.

urn:lsid:zoobank.org:act:D5EE0C3D-4998-4C5F-9969-0C69485E7C5F

http://species-id.net/wiki/Orthomorpha_subtuberculifera

**Figure 22.**
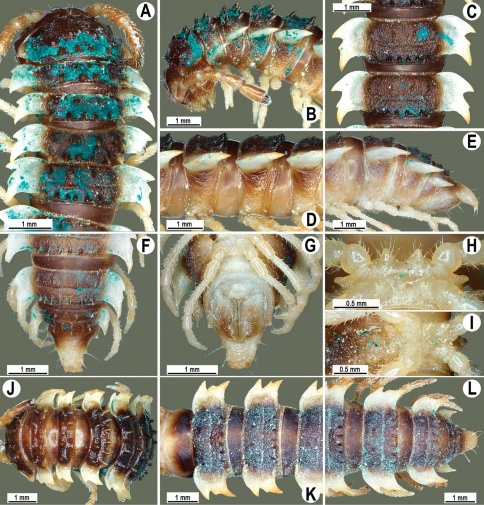
*Orthomorpha subtuberculifera* sp. n., ♂ holotype. **A, B, J** anterior part of body, dorsal, lateral and dorsal views, respectively **C, D**, K segments 10 and 11, dorsal, lateral and dorsal views, respectively **E–G**, L posterior part of body, lateral, dorsal, ventral and dorsal views, respectively **H, I** sternal cones between coxae 4, subcaudal and sublateral views, respectively.

Figs 22 [Fig F23] 24

#### Holotype.

♂ (CUMZ), Thailand, Nakhon Ratchasima Prov., Wang Nam Khiao Distr., Sakaerat Enviromental Research Station, 14°50'N, 102°34'E, 15.07.2006, leg. S. Panha.

#### Paratypes.

1 ♂ (CUMZ), same data as holotype. 1 ♂, 3 ♀ (CUMZ), same locality, 10.01.2007, leg. N. Likhitrakarn.

#### Name.

To emphasize the strong similarity to *Orthomorpha tuberculifera* sp. n.

#### Diagnosis.

Comes closest to *Orthomorpha tuberculifera* sp. n., but differs in a strong anterolateral incision on paraterga (see also Key below).

#### Description.

Length 21–23 mm (♂), 23–24 mm (♀), width of midbody pro- and metazona 2.2–2.4 and 3.5–4.2 mm (♂), 2.1–3.7 and 3.4–4.3 mm (♀), respectively. Coloration of alcohol material after preservation uniformly blackish-brown (♀) or apparently faded to uniformly brown (♂), with paraterga, venter, distal part of epiproct and several basal podomeres more flavous, pallid to light yellow (Fig. 13B-J); antennomere 7 infuscate, brown to dark brown; legs sometimes infuscate distally, light brown.

All other characters as in *Orthomorpha tuberculifera* sp. n. (Fig. 13), except as follows.

Antennae longer (Fig. 22B), extending behind segment 3 (♂) or 2 (♀) dorsally.

Head in width < collum < segments 3–4 < 2 < 5–16 (♂, ♀); thereafter body gently and gradually tapering. Paraterga on collum slightly declivous, subtriangular, with a small, but evident indentation near midway and a small, caudally directed, sharp denticle at caudal corner, the latter not extending behind tergal margin (Fig. 22A & J). Paraterga very strongly developed (Fig. 22A-G & J-L), especially well so in ♂, mostly subhorizontal to faintly upturned, always lying below dorsum, set at about 1/4 midbody height; shoulders well-developed, mostly straight; caudal tips of paraterga pointed, always extending behind tergal margin. Calluses delimited by a sulcus both dorsally and ventrally, especially deeply so dorsally, rather broad, with three evident lateral incisions on callus 2 (front indentation being smallest) and two strong indentations on following segments (front one being extremely strong, middle one smallest) (Fig. 22A, C, F & J-L). Posterior edge of paraterga always strongly concave, more strongly so on segments 16–19 (Fig. 22F & L). Pleurosternal carinae large, roughly granulate crests with a distinct tooth both frontally and caudally, complete on segments 2–7 (♂, ♀), thereafter split into both front and caudal teeth, the former increasingly strongly reduced until segment 15 (♂) or 16 (♀), the latter tooth gradually reduced until segment 17 (♂) or 18 (♀), much more strongly developed in ♂ than in ♀ (Fig. 22B, D & E). Epiproct (Fig. 22E, F & L) with pre-apical papillae place closer to tip.

Sterna delicately and sparsely setose. Only small paramedian knobs in front of gonopod aperture. Legs moderately long and slender, almost not incrassate in ♂, midbody ones ca 1.2–1.3 (♂) or 0.9–1.1 times (♀) as long as body height, prefemora without modifications.

**Figure 23. F23:**
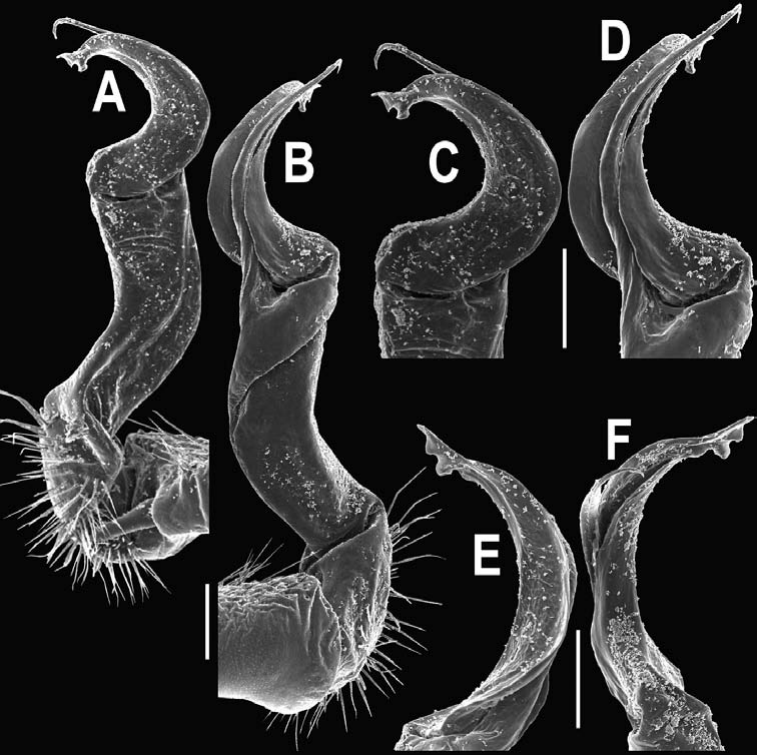
*Orthomorpha subtuberculifera* sp. n., ♂ paratype. **A, B** right gonopod, mesal and lateral views, respectively **C-F** distal part of right gonopod, mesal, lateral, subcaudal and suboral views, respectively. Scale bar: 0.2 mm.

**Figure 24.**
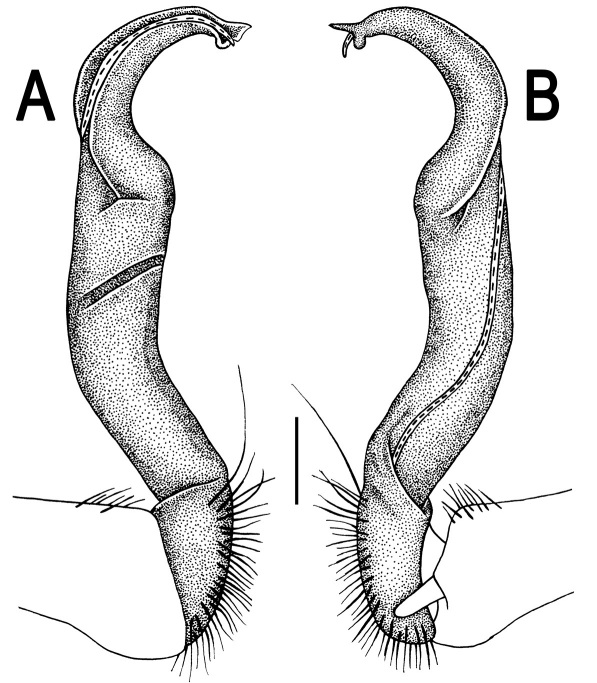
*Orthomorpha subtuberculifera* sp. n., ♂ holotype. **A, B** right gonopod, lateral and mesal views, respectively. Scale bar: 0.2 mm.

Gonopods (Figs 23 & 24) simple. Prefemoral part densely setose, less than 2 times shorter than femorite + “postfemoral” part. Femorite rather stout, slightly curved, nearly not enlarged distad, with a “postfemoral” part demarcated by an oblique lateral sulcus. Solenophore tip bifid, with terminal tooth bearing a minute denticle at base.

#### Remarks.

This new species has been found rather close to the localities of *Orthomorpha tuberculifera* sp. n., but northeast of the Sankamphaeng Mountain Range, Khao Yai National Park (Map 2).

### Orthomorpha communis sp. n.

urn:lsid:zoobank.org:act:99652C2E-2FA9-478F-9D12-8857E915B1B9

http://species-id.net/wiki/Orthomorpha_communis

**Figure 25.**
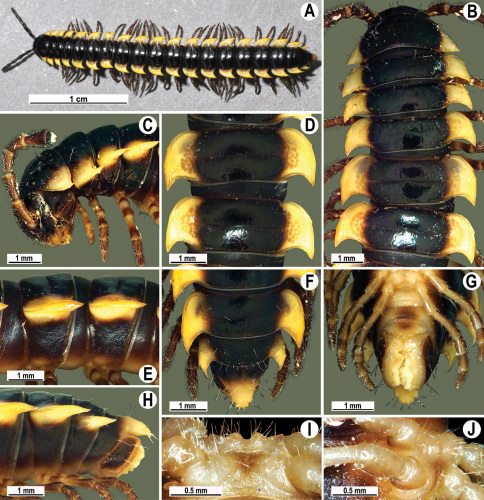
*Orthomorpha communis* sp. n., ♂ holotype (**B–J**) and ♂ paratype from Tabaek Watefall (**A**). **A** habitus, live coloration **B, C** anterior part of body, dorsal and lateral views, respectively **D, E** segments 10 and 11, dorsal and lateral views, respectively **F, G, H** posterior part of body, dorsal, ventral and lateral views, respectively **I, J** sternal cones between coxae 4, subcaudal and sublateral views, respectively.

Figs 25 [Fig F26] 27

#### Holotype.

♂ (CUMZ), Thailand, Surin Prov., Mueang Surin Distr., Khao Phanom Sawai National Park, ca 200 m, 14°15'45"N, 103°22'07"E, 26.04.2009, leg. N. Likhitrakarn.

#### Paratypes.

1 ♂, 1 ♀ (ZMUC), 1 ♂, 1 ♀ (ZMUM), 3 ♀ (CUMZ), same data, together with holotype. 2 ♂ (CUMZ), Prachinburi Prov., Prachantakham Distr., Takror Waterfall, ca 30 m, 14°10'53"N, 101°35'32"E, 18.09.2009, leg. N. Likhitrakarn. 1 ♂ (ZMUC), 1 ♂ (ZMUM), 1 ♀(CUMZ), same Prov., Na Di Distr., Nong Tabaek Waterfall, ca 40 m, 14°07'53"N, 101°40'41"E, 18.09.2009, leg. N. Likhitrakarn. 1 ♂ (CUMZ), Ubon Ratchathani Prov., Khong Chiam Distr., Patam National Park, ca 210 m, 15°23'55"N, 105°30'27"E, 25.04.2009, leg. P. Pimvichai. 2 ♀ (ZMUC), 2 ♀ (ZMUM), 2 ♂, 6 ♀ (CUMZ), same Distr., Tadtong Waterfall, ca 170 m, 15°15'14"N, 105°28'41"E, 14.05.2011, leg. N. Likhitrakarn.

#### Name.

To emphasize this species being quite common in the eastern part of Thailand close to the border with Cambodia.

#### Diagnosis.

Differs in unequal terminal lobes of the solenophore, both of which show a minute tooth near their bases, coupled with pointed, subtriangular paraterga on the collum etc. (see also Key below).

#### Description.

Length 31–38 (♂) or 32–38 mm (♀), width of midbody pro- and metazona 2.6–4.0 and 4.2–4.7 mm (♂), 3.2–3.8 and 4.8–5.3 mm (♀), respectively.

Coloration of live animals (Fig. 25A) blackish-brown, paraterga and epiproct contrasting creamy yellow, antennae dark brown, legs brownish; coloration of alcohol material after preservation (Fig. 25B-J) uniformly blackish-brown with contrasting light yellowish-brown paraterga and epiproct, tip of antennae pallid, venter and basal 3–4 podomeres brown to grey-brown.

Clypeolabral region densely setose, vertex sparsely setose, epicranial suture distinct. Antennae moderately long, clavate (antennomere 6 broadest), extending behind body segment 2 (♂) (Fig. 25A & C) or collum (♀) dorsally. Head in width < collum < segments 3 and 4 < 2 < 5–16 (♂, ♀); thereafter body gently and gradually tapering. Collum with three transverse rows of setae: 4+4 anterior, 2+2 intermediate, and 3+3 posterior; paraterga subtriangular, lying in a slightly rugulose posterior 1/3 of collum, slightly declined ventrally and continuing collum convexity (Fig. 25B); caudal corner of paraterga pointed. Tegument smooth and shining, prozona very finely shagreened, metazona leathery, faintly rugulose, below paraterga microgranulate. Metaterga 2–18 with an anterior transverse row of 2+2, mostly abraded setae; caudal row barely traceable only as 3+3 or 4+4 insertion points better visible laterally as minute knobs or oblong wrinkles. Metatergum 19 with 3+3 anterior and 4+4 posterior setae, the latter also borne on minute knobs. Tergal setae simple, rather long, about 1/3 metatergal length. Axial line barely traceable only on some metaterga, never complete. Paraterga very strongly developed (Fig. 25A-H), especially well so in ♂, all lying below dorsum (at about 1/3 body height), subhorizontal, in lateral view modestly enlarged on pore-bearing segments, thinner on poreless ones; shoulders always present, regularly rounded and narrowly bordered, fused to callus; caudal tip of all paraterga pointed, beak-like, lying within rear tergal margin or almost so on segments 2–7, thereafter extending increasingly beyond it, best developed and slightly curved mesad on segments 17–19 (Fig. 25F). Calluses delimited by a sulcus both dorsally, and, albeit more poorly so, ventrally, in dorsal view narrower on poreless segments than on pore-bearing ones, with three small, but evident lateral incisions on callus 2, with two similar incisions on following poreless segments, with one, often setigerous incision in front of pore on pore-bearing segments. Posterior edge of paraterga evidently concave, especially strongly so on segments 16–19. Ozopores evident, lateral, lying in an ovoid groove at about 1/4 in front of caudal corner. Transverse sulcus highly incomplete and visible only mid-dorsally on segment 2, complete on metaterga 5–18, narrow, rather deep, reaching bases of paraterga, finely beaded at bottom, better developed in ♀. Stricture between pro- and metazona narrow and rather shallow, evidently beaded at bottom down to base of paraterga (Fig. 25B, D, E & H). Pleurosternal carinae complete crests with a sharp caudal tooth on segments 2 and 3, onward as increasingly poorly developed, flat ridges with small caudal teeth until segment 12, thereafter only as an increasingly small, sharp, caudal tooth on segment 16. Epiproct (Fig. 25F-H) conical, flattened dorsoventrally, with two evident apical papillae directed ventrocaudally, subtruncate at tip; pre-apical papillae large, lying close to tip. Hypoproct (Fig. 25G) subtriangular, caudal margin rounded, setiferous knobs at caudal edge very large and well-separated.

Sterna sparsely setose, without modifications; cross-impressions shallow; with a paramedian pair of very small, flat, strongly separated, setose knobs between ♂ coxae 4 (Fig. 25I & J). A paramedian pair of small tubercles in front of gonopod aperture. Legs moderately long and slender, slightly incrassate in ♂, midbody ones ca 1.1–1.3 (♂) or 0.8–0.9 times (♀) as long as body height, prefemora without modifications, ♂ tarsal brushes present only on legs 1–7.

**Figure 26. F26:**
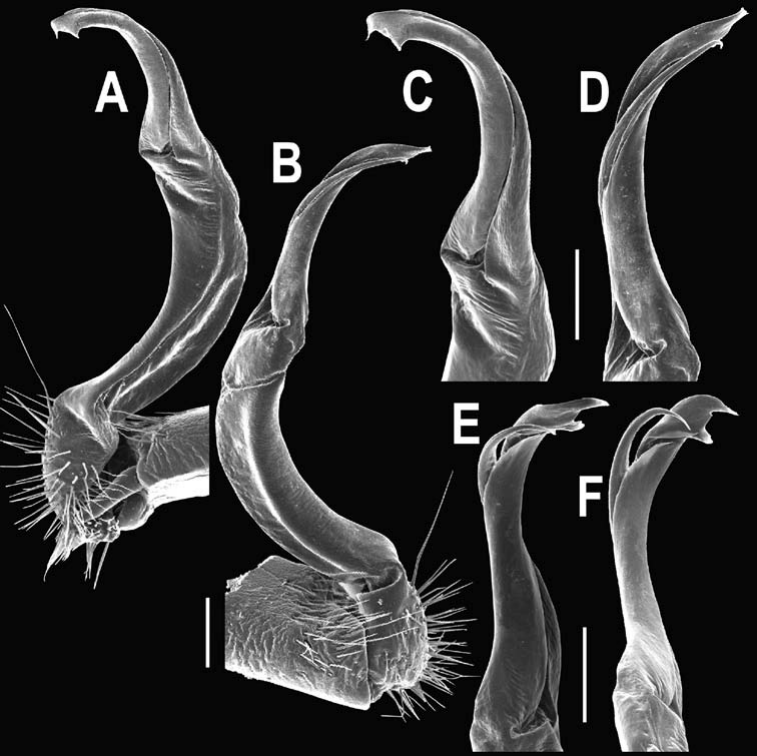
*Orthomorpha communis* sp. n., ♂ paratype from Khao Roo Chang. **A**, **B** right gonopod, mesal and lateral views, respectively **C**-**F** distal part of right gonopod, mesal, lateral, subcaudal and suboral views, respectively. Scale bar: 0.2 mm.

**Figure 27.**
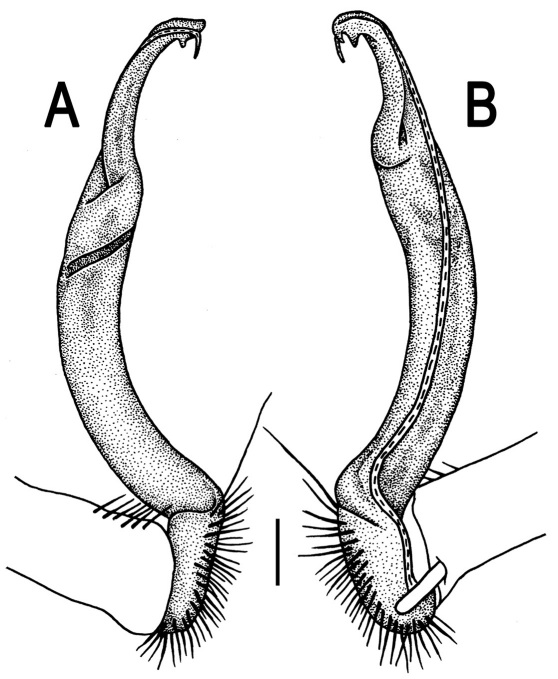
*Orthomorpha communis* sp. n., ♂ holotype. **A, B** right gonopod, lateral and mesal views, respectively. Scale bar: 0.2 mm.

Gonopods (Figs 26 & 27) simple. Coxa long and slender, with numerous strong setae distodorsally and distolaterally. Prefemur densely setose, nearly 3 times shorter than femorite + “postfemoral” part. Femorite slender, slightly curved and not enlarged distad, with a “postfemoral” part demarcated by an oblique lateral sulcus. Solenophore with a bidentate tip, terminal denticle a little larger than subterminal one, both being supplied with an extremely small indentation near base; solenomere long and flagelliform.

#### Remarks.

This new species appears to be quite widespread in the eastern part of Thailand close to the border with Cambodia (Map 2).

### Orthomorpha atypica sp. n.

urn:lsid:zoobank.org:act:8D3CA11A-5DFF-4F3A-AA8E-20691FDB6893

http://species-id.net/wiki/Orthomorpha_atypica

**Figure 28.**
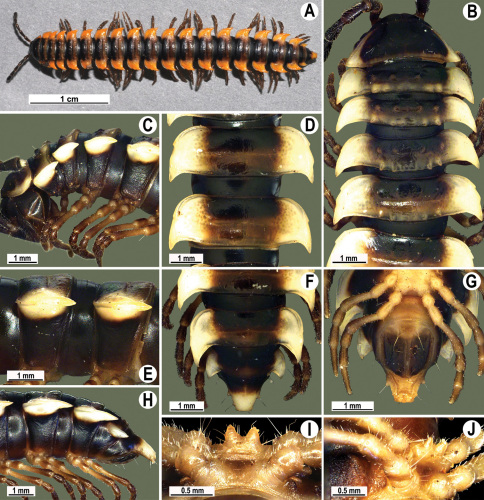
*Orthomorpha atypica* sp. n., ♂ holotype. **A** habitus, live coloration **B, C** anterior part of body, dorsal and lateral views, respectively **D, E** segments 10 and 11, dorsal and lateral views, respectively **F, G, H** posterior part of body, dorsal, ventral and lateral views, respectively **I, J** sternal cones between coxae 4, subcaudal and sublateral views, respectively.

Figs 28 [Fig F29] 30

#### Holotype.

♂ (CUMZ), Thailand, Chanthaburi Prov., Khlung Distr., Troknong Waterfall, 12°54'29"N, 102°24'12"E, 01.10.2009, leg. C. Sutcharit & N. Likhitrakarn.

#### Paratypes.

25 ♂, 30 ♀ (CUMZ), 3 ♂, 3 ♀ (ZMUC), 3 ♂, 3 ♀ (ZMUM), same data, together with holotype.

#### Name.

To emphasize this species being not quite typical to readily fit into any of the former species groups.

#### Diagnosis.

Superficially, this new species strongly resembles *Orthomorpha communis* sp. n., especially as regards the color pattern, the degree of development of some paraterga, and size. Yet the two species differ markedly in the development in *Orthomorpha atypica* sp. n. of distinct sternal cones between ♂ coxae 4, coupled with a peculiar denticle placed on top of the outer lobule of the solenophore, not between the two terminal lobules characteristic of other species (see also Key below).

#### Description.

Length 32–39.5 (♂) or 34.5–44 mm (♀), width of midbody pro- and metazona 3.0–3.6 and 5.0–5.6 mm (♂), 3.6–4.0 and 5.5–6.4 mm (♀), respectively.

Coloration of live animals (Fig. 28A) blackish-brown with contrasting creamy light orange paraterga and epiproct, posterior halves of metaterga light yellow-brown to light brown, antennae blackish, legs dark brown; coloration of alcohol material after preservation (Fig. 25B-J) uniformly dark brown with contrasting pallid paraterga, epiproct and tip of antennae, legs brown to light grey-brown, posterior halves of metaterga light brown to brown.

Clypeolabral region densely setose, vertex barely setose, epicranial suture distinct. Antennae moderately long (Fig. 28A), clavate (antennomere 6 broadest), extending behind body segment 3 dorsally (♂, ♀). Head in width < collum < segments 3–4 < 2 < 5–16 (♂, ♀); thereafter body gently, and gradually tapering. Collum with three transverse rows of setae: 4+4 anterior, 2+2 intermediate and 3+3 posterior; posterior quarter evidently rugulose, mid-dorsal part with a superficial and shortened axial line; paraterga slightly declined ventrally, discontinuing dorsum’s convexity, subrectangular, nearly reaching caudal edge; caudal corner of paraterga very narrowly rounded (Fig. 28B). Tegument of metaterga shining, leathery, rugulose-tuberculate, especially well so on several anterior metaterga; prozona very finely shagreened, metazona below paraterga faintly rugulose, finely microgranulate only near coxae. Metaterga 2–5 with two rows of 2+2 anterior and 3(4)+3(4) setiferous cones, usually slightly smaller cones in anterior (pre-sulcus) row and more evident ones laterally in posterior row (Fig. 28B & C); thereafter same pattern, but traceable only as insertion points, tuberculation gradually growing obliterate to become nearly wanting on a few caudalmost metaterga. Metatergum 19 with 3+3 anterior and 4+4 posterior setae, the latter also borne on minute knobs or oblong wrinkles. Tergal setae very short, simple, about 1/5 metatergal length. Axial line barely traceable only on metaterga, slightly better visible in anterior halves than in posterior ones, always incomplete and sometimes missing. Paraterga very strongly developed (Fig. 28B-H), especially well so in ♂, all lying below dorsum (at about 1/3 body height), mostly subhorizontal (sometimes slightly upturned only on segments 2 and 3 in ♂ or only on segment 2 in ♀), in lateral view modestly enlarged on pore-bearing segments, thinner on poreless ones; shoulders always present, rather regularly rounded and narrowly bordered, fused to callus; caudal corner of all paraterga pointed, beak-like, extending increasingly beyond rear tergal margin, slightly curved mesad on segments 18 and 19 (Fig. 28F). Calluses delimited by a sulcus both dorsally and, albeit considerably more poorly so, ventrally, segment 2 with three very faint incisions at lateral edge, two similarly faint incisions on following poreless segments, one much stronger incision in front of pore sinuosity on pore-bearing segments. Posterior edge of paraterga evidently concave, especially strongly so on segments 16–19. Ozopores evident, lateral, lying in an ovoid groove at about 1/4 in front of caudal corner. Transverse sulcus evident (Fig. 28B-F & H), thin, deep and only slightly incomplete on metaterga 2–4, complete, at most very faintly beaded at bottom, reaching bases of paraterga on metaterga 5–18, barely visible, highly superficial and again incomplete on metatergum 19 in ♂; somewhat less strongly developed in ♀. Stricture between pro- and metazona narrow and rather shallow, evidently beaded at bottom down to base of paraterga. Pleurosternal carinae complete high crests with a sharp caudal tooth on segments 2–4(5), thereafter increasingly well divided into a front bulge and a caudal tooth, both increasingly strongly reduced in size, bulge until segment 14, tooth until segment 17 (♂), or carinae considerably lower, their caudal tooth strongly rounded and only barely traceable until segment 17 (♀). Epiproct (Fig. 28F-H) conical, flattened dorsoventrally, very faintly narrowed caudad, with two evident apical papillae directed caudally, subtruncate at tip; pre-apical papillae very small, lying close to tip. Hypoproct (Fig. 28G) semi-circular, caudal margin rounded, setiferous knobs at caudal edge medium-sized and well-separated.

Sterna sparsely setose, without modifications; cross-impressions shallow, especially so due to a superficial axial impression; a large, setose, transverse lobe bearing a paramedian pair of large, basally contiguous cones between ♂ coxae 4 (Fig. 28I & J). A paramedian pair of small, but evident tubercles in front of gonopod aperture. Legs moderately long and slender, slightly incrassate in ♂, midbody ones ca 1.2–1.3 (♂) or 0.8–1.0 times (♀) as long as body height, prefemora without modifications, ♂ tarsal brushes present only on legs 1–5(6).

**Figure 29. F29:**
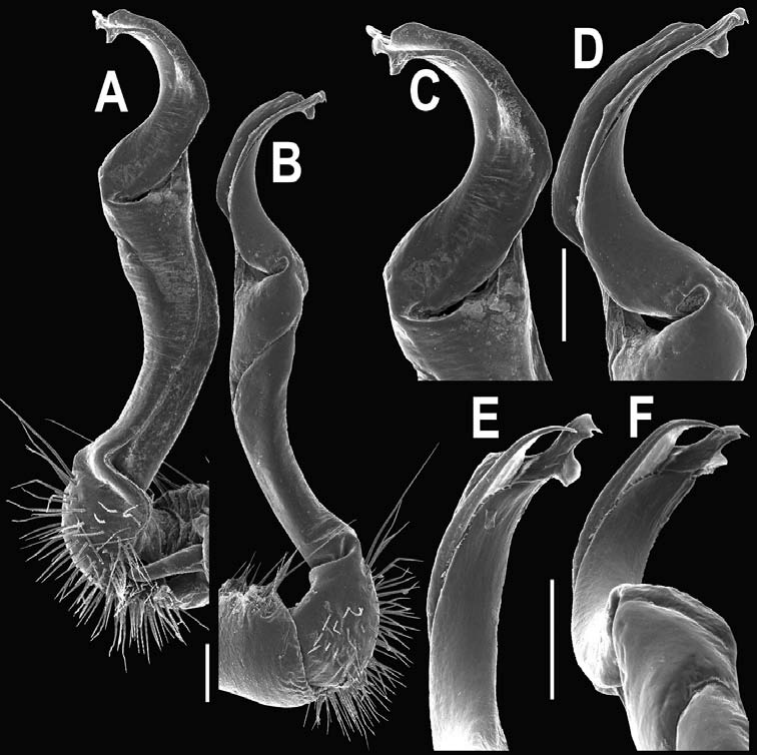
*Orthomorpha atypica* sp. n., ♂ holotype. **A, B** right gonopod, mesal and lateral views, respectively **C**-**F** distal part of right gonopod, mesal, lateral, suboral and subcaudal views, respectively. Scale bar: 0.2 mm.

**Figure 30.**
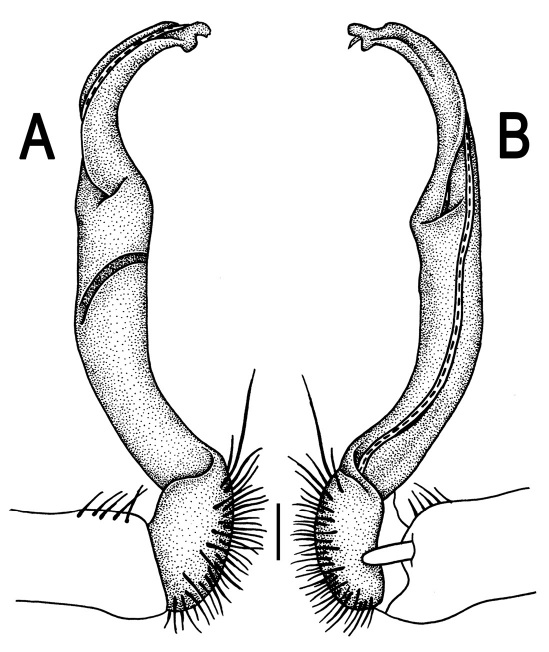
*Orthomorpha atypica* sp. n., ♂ holotype. **A, B** right gonopod, lateral and mesal views, respectively. Scale bar: 0.2 mm.

Gonopods (Figs 29, 30) simple. Coxa long and slender, with several strong setae distodorsally. Prefemur densely setose, less than half the length of femorite + “postfemoral” part. Femorite slender, slightly curved and nearly not enlarged distad, with a “postfemoral” part demarcated by an oblique lateral sulcus. Solenophore with a bidentate tip, both prongs being subequal, but terminal lobule with an unusual minute denticle near base; solenomere long and flagelliform.

### Orthomorpha latiterga sp. n.

urn:lsid:zoobank.org:act:BE36B116-1815-4FB2-9C46-FE1E0A3443D5

http://species-id.net/wiki/Orthomorpha_latiterga

Figs 31 33

#### Holotype.

♂ (CUMZ), Thailand, Chanthaburi Prov., Pong Nam Ron Distr., Hin Dard Waterfall, ca 260 m, 12°58'19"N, 102°14'21"E, 17.09.2009, leg. C. Sutcharit.

#### Name.

To emphasize the extremely broad paraterga.

#### Diagnosis.

Differs in the extremely broad paraterga, coupled with the pleurosternal carinae represented by complete high crests with a sharp caudal tooth on segments 2–7 (♂) etc. (see also Key below).

#### Description.

Length 31 mm, width of midbody pro- and metazona 3.1 and 5.0 mm, respectively.

**Figure 31.**
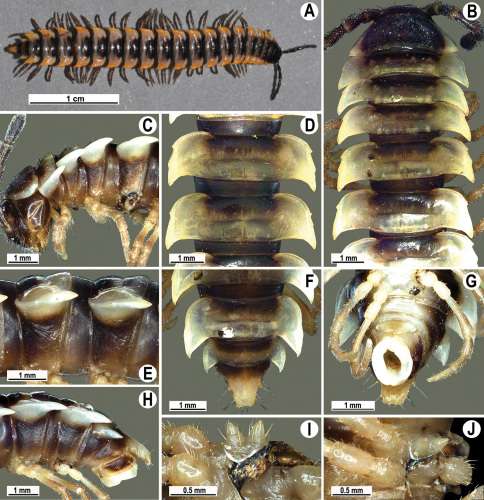
*Orthomorpha latiterga* sp. n., ♂ holotype. **A** habitus, live coloration **B, C** anterior part of body, dorsal and lateral views, respectively **D, E** segments 10 and 11, dorsal and lateral views, respectively **F, G, H** posterior part of body, dorsal, ventral and lateral views, respectively **I, J** sternal cones between coxae 4, subcaudal and sublateral views, respectively.

Live coloration (Fig. 31A) black-brown with mainly grey-brownish caudal halves of metaterga and bases of paraterga, and contrasting creamy light orange paraterga and epiproct; antennae blackish, legs light brown; coloration of alcohol material after preservation (Fig. 31B-J) rather uniformly dark brown with lighter caudal halves of metaterga and bases of paraterga, and contrasting pallid paraterga, epiproct and tip of antennae, legs brown to light grey-yellow.

Clypeolabral region sparsely setose, vertex bare, epicranial suture distinct. Antennae moderately long (Fig. 31A & C), extending behind body segment 3 dorsally. Head in width < collum < segments 3–4 < 5 < 2 < 6–16; thereafter body gently and gradually tapering. Collum with three transverse rows of setae, 3+3 anterior, 2+2 intermediate and 3+3 posterior; paraterga (Fig. 31B) only slightly declivous, broadly rounded, and narrowly bordered; caudal corner narrowly rounded, slightly declined ventrally, not extending behind tergal margin; posterior quarter of collum slightly rugulose. Tegument of metaterga shining, rugulose-tuberculate, especially on several front metaterga; prozona very finely shagreened, metazona below paraterga faintly rugulose. Metaterga 2–5 with two rows of 2+2 anterior and 3+3 setiferous cones, except segment 3 with 2+1 in anterior row; usually slightly smaller cones in anterior (pre-sulcus) row and more evident ones laterally in posterior row (Fig. 31B & C); thereafter same pattern, but traceable only as insertion points, tuberculation gradually growing obliterate to become nearly wanting from segment 11 on. Tergal setae short, simple, about 1/3 metatergal length. Axial line visible both on pro- and metazona. Paraterga extremely strongly developed (Fig. 31B-H), broad, all lying below dorsum (at about 1/3 body height), mostly subhorizontal, slightly upturned on segments 2–5 and 18–19, in lateral view modestly enlarged on pore-bearing segments, thinner on poreless ones (Fig. 31E); shoulders always present, mostly nearly straight and narrowly bordered, fused to callus; caudal corner of most of paraterga very narrowly rounded, extending increasingly beyond tergal margin, slightly curved mesad on segments 16–19 (Fig. 31F). Calluses delimited by a sulcus only dorsally, segment 2 with three evident incisions at lateral edge, following segments with two lateral incisions, front one being particularly evident. Posterior edge of paraterga evidently concave, especially strongly so on segments 16–19. Ozopores evident, lateral, lying in an ovoid groove at about 1/3 in front of caudal corner. Transverse sulcus evident (Fig. 31B-F & H), narrow, rather shallow and only slightly incomplete on metaterga 2 and 3, complete, smooth at bottom, reaching base of paraterga on metaterga 4–18. Stricture between pro- and metazona narrow and shallow, evidently beaded at bottom down to base of paraterga. Pleurosternal carinae complete high crests with a sharp caudal tooth on segments 2–7, thereafter increasingly well divided into a front bulge and a caudal tooth, both increasingly strongly reduced in size, bulge until segment 14, tooth until segment 17. Epiproct (Fig. 31F-H) conical, flattened dorsoventrally, very faintly narrowed caudad, subtruncate, with two evident apical papillae directed caudally, both pointed at tip; pre-apical papillae very small, lying close to tip. Hypoproct (Fig. 31G) subtrapeziform, caudal margin rounded, setiferous knobs at caudal edge medium-sized and well-separated.

Sterna sparsely setose, without modifications; cross-impressions shallow; lobe between ♂ coxae 4 much like in *Orthomorpha atypica* sp. n., but cones more acute (Fig. 31I & J). A paramedian pair of small, but evident tubercles in front of gonopod aperture. Legs moderately long and slender, midbody ones ca 1.2–1.3 as long as body height, prefemora without modifications, ♂ tarsal brushes present only on legs 1–5.

**Figure 32.**
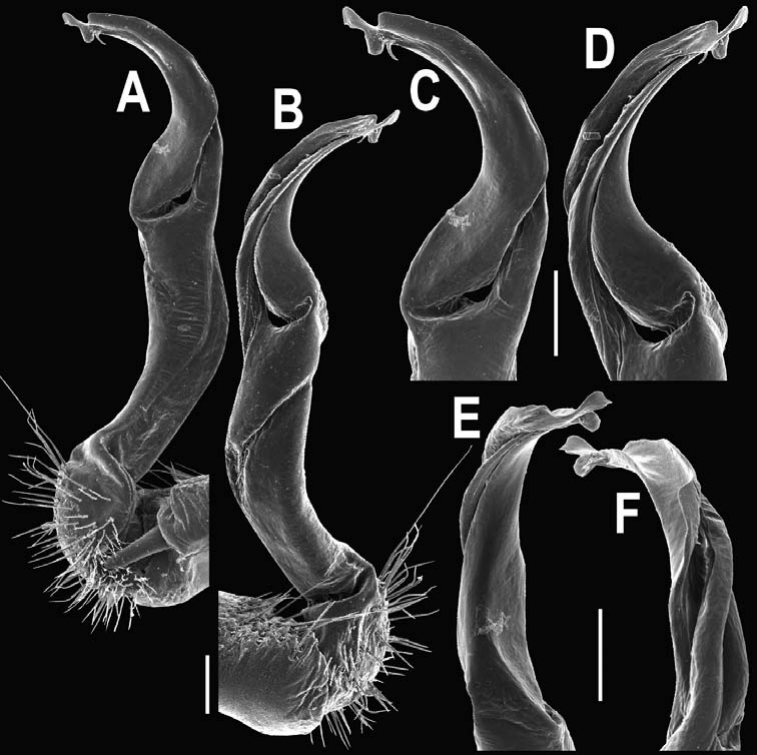
*Orthomorpha latiterga* sp. n., ♂ holotype. **A, B** right gonopod, mesal and lateral views, respectively **C**-**F** distal part of right gonopod, mesal, lateral, suboral and subcaudal views, respectively. Scale bar: 0.2 mm.

**Figure 33.**
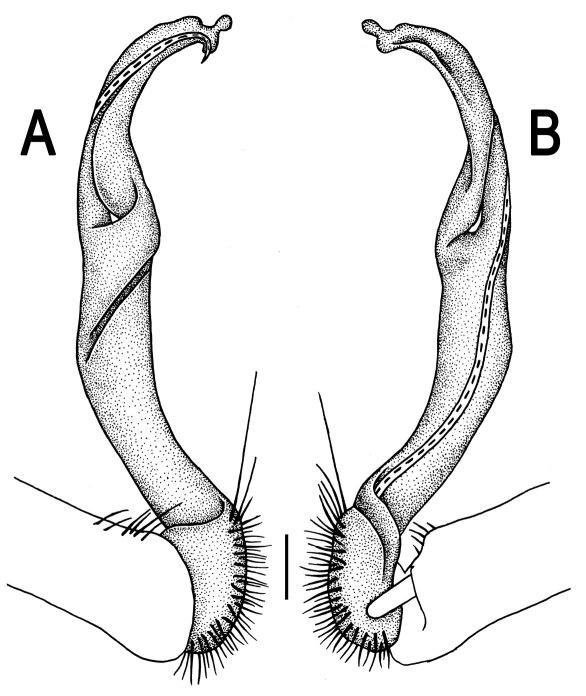
*Orthomorpha latiterga* sp. n., ♂ holotype. **A, B** right gonopod, lateral and mesal views, respectively. Scale bar: 0.2 mm.

Gonopods (Figs 32, 33) much like in *Orthomorpha atypica* sp. n., but solenophore tip with more distinct apical lobules.

#### Remarks.

This new species shows the paraterga relatively perhaps among the broadest amongst congeners.

### Orthomorpha suberecta sp. n.

urn:lsid:zoobank.org:act:2D635CF6-C48E-45E4-856D-04B860D4DA7C

http://species-id.net/wiki/Orthomorpha_suberecta

**Figure 34.**
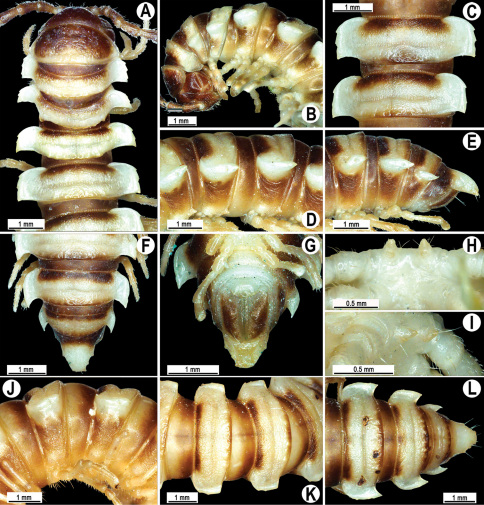
*Orthomorpha suberecta* sp. n., ♂ holotype (**A-I**), ♀ paratype (**J–L**). **A, B** anterior part of body, dorsal and lateral views, respectively **C, D, J, K** segments 10 and 11, dorsal, lateral, lateral and dorsal views, respectively **E-G, L** posterior part of body, lateral, dorsal, ventral and dorsal views, respectively **H, I** sternal cones between coxae 4, subcaudal and sublateral views, respectively.

Figs 34 [Fig F35] 36

#### Holotype.

♂ (CUMZ), Thailand, Nong Bua Lamphu Prov., Suwannakhuha Distr., near Suwannakhuha Cave, 18°0'09"N, 102°28'28"E, 17.10.2007, leg. S. Panha.

#### Paratypes.

1 ♀ (CUMZ), same data, together with holotype.

#### Name.

To emphasize the suberect gonopod femorite.

#### Diagnosis.

Differs in a suberect gonofemorite, coupled with mostly flavous metaterga and widely separated sternal cones between ♂ coxae 4 (see also Key below).

#### Description.

Length ca 26 (♂) or 28 mm (♀), width of midbody pro- and metazona 2.5 and 3.9 mm (♂), 3.0 and 4.3 mm (♀), respectively.

Coloration of alcohol material after long-term preservation castaneous brown with a pattern of contrasting whitish to light brown paraterga and epiproct, and mostly greyish-white to light brownish posterior halves of postcollum metaterga, with caudal edges and front 1/4 of metaterga, as well as surface below paraterga and entire rear halves of prozona brown to dark brown; head and antennomeres 6 and 7 brown to dark brown; venter and a few basal podomeres light brownish to brown, legs growing infuscate (brown) distally (Fig. 34A-L).

Clypeolabral region sparsely setose, vertex bare, epicranial suture distinct. Antennae moderately long, clavate (antennomere 6 broadest), extending behind body segment 2 (♂, ♀) dorsally. Head in width < collum < segments 2 < 3 < 4 < 5–17 (♂), or head < segment 3–4 < 2 < 5–17 (♀); thereafter body gently and gradually tapering. Collum with three transverse rows of setae: 4+4 anterior, 2+2 intermediate and 4+4 posterior; paraterga slightly declivous, broadly rounded and narrowly bordered, caudal corner a small knob not extending behind tergal margin (Fig. 34B). Tegument rather strongly shining, prozona very finely shagreened, metazona leathery, rugulose, below paraterga microgranulate and rugulose. Postcollum metaterga with an anterior transverse row of 2+2, mostly abraded setae; caudal row barely traceable as 3+3 insertion points, laterally as small knobs increasingly strongly reduced towards segment 19. Tergal setae simple, rather long, about 1/3 metatergal length, mostly abraded. Axial line visible both on pro- and metazona. Paraterga very strongly developed (Fig. 34A-G & J-L), especially well so in ♂, all lying below dorsum (at about 1/3 body height), subhorizontal, in lateral view modestly enlarged on pore-bearing segments, thinner on poreless ones; shoulders broadly rounded, and narrowly bordered, fused to callus; caudal tip of all paraterga pointed, beak-like, lying within rear tergal margin, thereafter extending increasingly beyond it, best developed and slightly curved mesad on segments 18 and 19 (Fig. 34F & G). Calluses delimited by a sulcus both dorsally and ventrally on segments 5–18. Paraterga 2 broad, anterior edge convex, lateral edge with four small acute denticles (Fig. 34A). Following poreless segments with two similar incisions, with one, often setigerous incision lying in front of pore on pore-bearing segments. Posterior edge of paraterga slightly concave, especially strongly so on segments 18 and 19. Ozopores evident, lateral, lying in an ovoid groove at about 1/4 in front of caudal corner. Transverse sulcus visible on metaterga 5–18, narrow, not reaching bases of paraterga, ribbed at bottom. Stricture between pro- and metazona narrow and rather shallow, faintly beaded at bottom down to base of paraterga (Fig. 34B-F & K). Pleurosternal carinae complete crests with a sharp caudal tooth on segments 2–7 (♂) or 2–6 (♀), thereafter increasingly well divided into a front bulge and a caudal tooth, both increasingly strongly reduced in size until segment 17 (♂, ♀). Epiproct (Fig. 34E-G & L) conical, flattened dorsoventrally, with two evident apical papillae directed ventrocaudally, acute at tip; pre-apical papillae small denticles lying close to tip. Hypoproct (Fig. 34G) subtriangular, caudal margin rounded, setiferous knobs at caudal edge evident and well-separated.

Sterna sparsely setose, without modifications; cross-impressions shallow; a paramedian pair of evident, small, fully separated, setose cones between ♂ coxae 4 (Fig. 34I & J). A paramedian pair of small, but evident tubercles in front of gonopod aperture. Legs moderately long and slender, slightly incrassate in ♂, midbody ones ca 0.9–1.0 (♂) or 0.8–0.9 times (♀) as long as body height, prefemora without modifications, ♂ tarsal brushes absent.

**Figure 35. F35:**
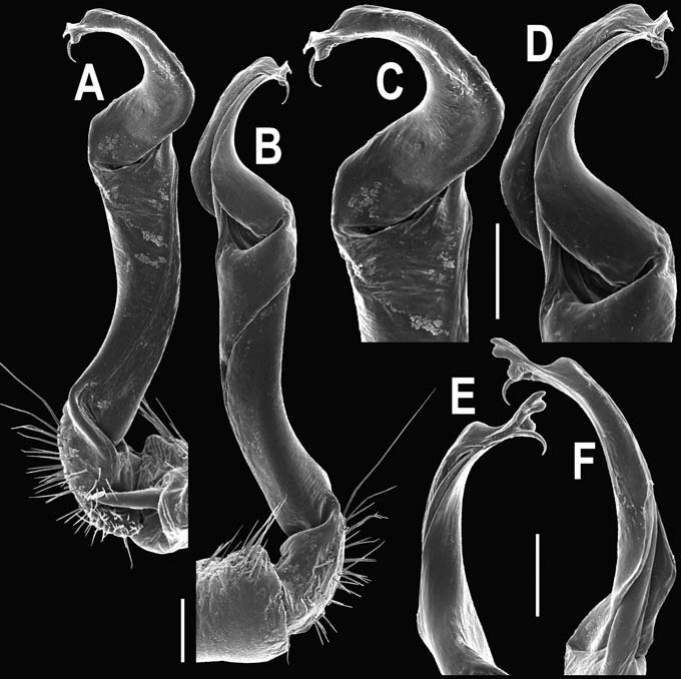
*Orthomorpha suberecta* sp. n., ♂ holotype. **A, B** right gonopod, mesal and lateral views, respectively **C**-**F** distal part of right gonopod, mesal, lateral, suboral and subcaudal views, respectively. Scale bar: 0.2 mm.

**Figure 36.**
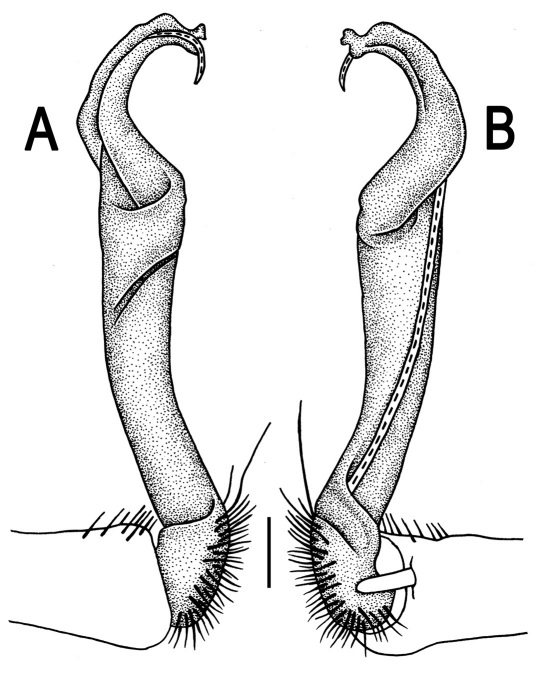
*Orthomorpha suberecta* sp. n., ♂ holotype. **A, B** right gonopod, lateral and mesal views, respectively. Scale bar: 0.2 mm.

Gonopods (Figs 35 & 36) simple. Coxa long and slender, with several strong setae distodorsally. Prefemur densely setose, about half the length of femorite + “postfemoral” part. Femorite suberect, slender, nearly not enlarged distad, with a “postfemoral” part demarcated by an oblique lateral sulcus. Solenophore with lamina lateralis evidently smaller than lamina medialis, tip distinctly bilobed, terminal lobule being vaguely bifid; solenomere long and flagelliform.

### Orthomorpha paviei

Brölemann, 1896

http://species-id.net/wiki/Orthomorpha_paviei

Orthomorpha Paviei Brölemann 1896: 332 (D).
Orthomorpha Paviei – Brölemann 1904: 8 (D); Attems 1898: 339 (M).
?Prionopeltis Paviei – Attems 1914: 204 (M).
Pratinus paviei – Attems 1937: 122 (M).
Orthomorpha paviei – Jeekel 1963: 265 (M); 1964: 359 (M); 1968: 56 (M); Golovatch 1998: 42 (D); Enghoff et al. 2004: 38 (M); Enghoff 2005: 97 (M).

#### Remarks.

This species has long been acknowledged as being quite disjunct due to the gonopod showing a parabasally twisted femorite, while the tip is rather deeply bifid, somewhat intermediate between that of typical *Antheromorpha* Jeekel, 1968 and *Orthomorpha* (see Brölemann 1904). However, both (Jeekel (1963, 1964, 1968) and Golovatch (1998) included it in *Orthomorpha*. We still are inclined to treat *Orthomorpha paviei* as a distinct species of *Orthomorpha*, showing the gonopod tip supplied with a remarkable spine strongly resembling that in *Orthomorphoides* species (see below).

##### Species with a trifid gonopod tip, with a terminal, a middle and a subterminal denticle or lobule

### Orthomorpha hydrobiologica

Attems, 1930

http://species-id.net/wiki/Orthomorpha_hydrobiologica

Figs 37 38

Orthomorpha hydrobiologica Attems 1930a: 120 (D).
Orthomorpha hydrobiologica – Attems 1937: 63 (D); 1938: 215 (R); Jeekel 1963: 265 (M); 1964: 361 (M, D); 1968: 45 (M); Hoffman 1973: 362 (M); 1977: 700 (M); Golovatch 1998: 42 (M); Enghoff et al. 2004: 38 (R).
Oxidus hydrobiologicus – Chamberlin 1945: 10 (R).

#### Holotype.

♂ (NHMW-3507), Indonesia, East Java Prov., Lumajang Regency, shore of Ranu (= lake) Bedali, 10.10.1928, leg. A. F. Thienemann & H. J. Feuerborn.

#### Other material examined.

3 ♂, 3 ♀ (NHMW-8004), Vietnam, Khanh Hoa Prov., Cua Bé (4–6 km from Nha Trang), no date, det. C. Attems. 2 ♂, 1 ♀ (NHMW-8005), same Prov., Nha Trang, no date, det. Attems. 4 ♂, 4 ♀ (NHMW-8007), Vietnam, Tinh Soc Trang (? or Bà Ria-Vũng Tàu), Poulo Condore Island, no date, leg. C. Dawydoff, det. C. Attems. 4 ♂, 1 ♀ (NHMW-8006), Cambodia, Kampot Prov., Bokor Mt in Damrei Mountains (= Elephant Mountains), near Bok Kor Village, no date, leg. C. Dawydoff, det. C. Attems. 4 ♂, 1 ♀ (NHMW-8008), Indonesia, West Java Prov., Tjisaroea, leg. W. S. S. van Benthem-Jutting, det. C. Attems. 7 ♂, 8 ♀, 2 juv. (NHMW-8009), Indonesia, East Java Prov., Bondowoso, Ijen Caldera, no date, det. C. Attems.

#### Redescription.

**Figure 37.**
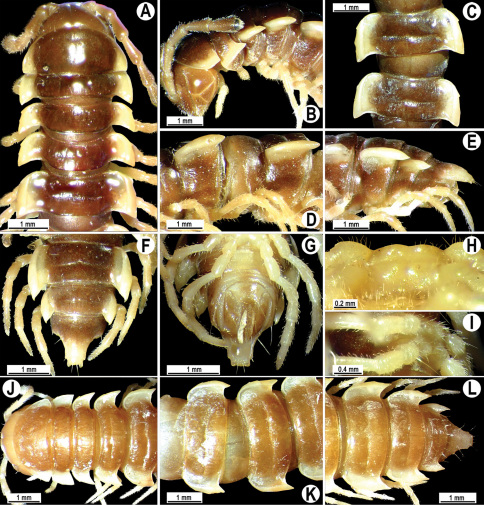
*Orthomorpha hydrobiologica* Attems, 1937, ♂ holotype (**A–I**), ♀ from Cua Bé (**J–L**). **A, B, J** anterior part of body, dorsal, lateral and dorsal views, respectively **C, D, K** segments 10 and 11, dorsal, lateral and dorsal views, respectively **E-G, L** posterior part of body, lateral, dorsal, ventral and dorsal views, respectively **H, I** sternal cones between coxae 4, subcaudal and sublateral views, respectively.

Length 17–30 mm (♂), 19–29 mm (♀), width of midbody pro- and metazona 1.4–2.2 and 2.0–3.1 mm (♂), 1.6–2.7 and 2.0–3.4 mm (♀), respectively (vs 3.3 mm in holotype, up to 4.0 mm in width, as given in the available descriptions (Attems 1930a, 1937)). Coloration of alcohol material upon long-term preservation rather uniformly light brown to brown (Fig. 37) (vs dark castaneous brown, as given in the original description (Attems 1930a)) with contrasting light yellow paraterga and epiproct.

Head usual, clypeolabral region densely setose, surface of vertex smooth (vs a pair of strong setae, as given in the descriptions (Attems 1930a, 1937)), epicranial suture distinct. Antennae rather long and slender (Fig. 37A, B & J), extending behind until body segment 3 (♂) or reaching segment 3 (♀) dorsally. Head in width < collum < segments 3 and 4 < segment 2 < segments 5–16, gently and gradually tapering thereafter. Collum with three transverse rows of setae, 4+4 anterior, 2+2 intermediate, and 3+3 posterior setae; caudal corner of paraterga subrectangular, narrowly rounded (Fig. 37A, B & J). Tegument smooth and shining, prozona very finely shagreened, metaterga finely rugulose, surface below paraterga finely microgranulate. Postcollum metaterga with two transverse rows of setae traceable as insertion points: 2+2 in anterior (pre-sulcus) row, 3+3 in posterior (postsulcus) one. Axial line barely visible, starting from collum. Paraterga very strongly developed (Fig. 37A-G, J-L), especially so in ♂, mostly slightly upturned, lying below dorsum, thin in lateral view, like blunt blades, a little thicker only on pore-bearing segments, on postcollum segments extending increasingly beyond rear tergal margin, from segment 14 pointed, on segments 16–19 tips strongly curved mesad. Calluses on paraterga 2 delimited by a sulcus only dorsally, on following paraterga both dorsally and ventrally, rather broad, especially so on pore-bearing segments. Paraterga 2 broad, anterior edge rounded, lateral edge with one larger and two smaller, but evident incisions in anterior half; posterior edge evidently concave (Fig. 37A, B & J). Paraterga 3 and 4 subequal, like subsequent paraterga, anterior edge broadly rounded, bordered and fused to callus, lateral edge with two small incisions in anterior half. Ozopores evident, lateral, lying in an ovoid groove at about 1/3 of metazonital length. Transverse sulcus complete on metaterga 5–17, incomplete on metaterga 4 and 18, shallow, not reaching bases of paraterga, ribbed at bottom, slightly sinuate anteromedially (Fig. 37A, C, F & J-L). Stricture between pro- and metazona rather narrow, shallow, beaded at bottom down to base of paraterga. Pleurosternal carinae complete crests only on segments 2–4 (♂, ♀) (Fig. 37B, D & E), each with an evident sharp denticle caudally, thereafter increasingly strongly reduced until segment 17 (♂) or 16 (♀). Epiproct (Fig. 37E, F, G & L) conical, flattened dorsoventrally, apical papillae small; tip subtruncate; pre-apical papillae small, but visible, lying close to tip. Hypoproct (Fig. 37G) subtrapeziform, setiferous knobs at a slightly convex caudal margin rather large and well-separated.

Sterna sparsely setose, without modifications, but with two small, low, rounded, fully separated, setose knobs between ♂ coxae 4 (Fig. 37H & I). A paramedian pair of conspicuous, high tubercles in front of gonopod aperture. Legs long and slender, only slightly incrassate in ♂, midbody ones ca 1.2–1.4 (♂) or 0.9–1.1 times (♀) as long as body height, prefemora without modifications, tarsal brushes present until ♂ legs 7.

**Figure 38.**
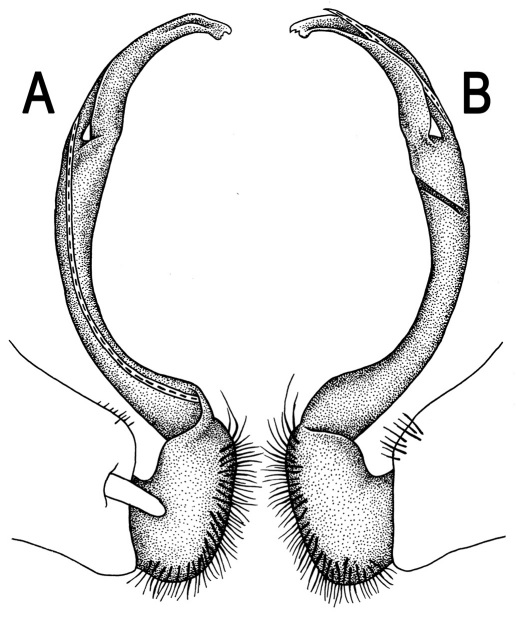
*Orthomorpha hydrobiologica* Attems, 1930, ♂ holotype. **A, B** left gonopod, mesal and lateral views, respectively.

Gonopods (Fig. 38) simple. Coxa long and slender, with several setae distodorsally. Prefemur enlarged, densely setose, less than 2 times shorter than femorite + “postfemoral” part. Femorite very slender, evidently curved, “postfemoral” part demarcated by an oblique lateral sulcus; tip of solenophore small, trifid, with two subequal denticles (one terminal, the other middle) and a broader subterminal lobule.

#### Remarks.

This species was originally described in two varieties, each based on a single male: *Orthomorpha hydrobiologica* var. *hydrobiologica*, from the shores of Lake Bedali, eastern Java, and *Orthomorpha hydrobiologica* var. *unicolor*, from Sarangan, central Java,Indonesia (Attems 1930a). The holotype of *Orthomorpha hydrobiologica* var. *hydrobiologica* has been redescribed above, also including some more non-type samples identified as such by Attems himself. As regards *Orthomorpha hydrobiologica* var. *unicolor*, it proves to be a distinct species, likewise redescribed from the holotype just below.

### Orthomorpha unicolor

Attems, 1930

http://species-id.net/wiki/Orthomorpha_unicolor

Figs 39 40

Orthomorpha hydrobiologica var. *unicolor*Attems 1930a: 122 (D).
Orthomorpha hydrobiologica var. *unicolor* – Attems 1937: 64 (D).
Orthomorpha unicolor – Jeekel 1963: 265 (M); 1964: 361 (M); 1968: 45 (M); Hoffman 1973: 362 (M); 1977: 700 (M); Golovatch 1998: 42 (D, M).

#### Holotype.

♂ (NHMW-3508), Indonesia, central Java, Sarangan, 09.12.1928, leg. A. F. Thienemann & H. J. Feuerborn.

#### Redescription.

**Figure 39.**
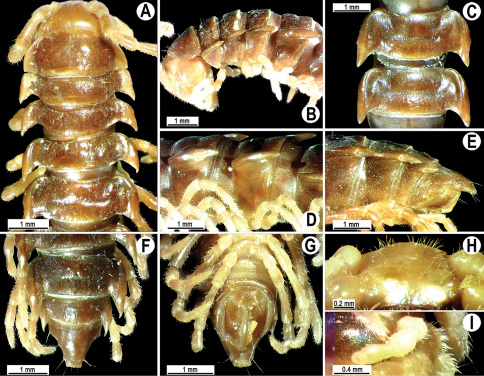
*Orthomorpha unicolor* Attems, 1930, ♂ holotype. **A, B** anterior part of body, dorsal and lateral views, respectively **C, D** segments 10 and 11, dorsal and lateral views, respectively **E-F** posterior part of body, lateral, dorsal and ventral views, respectively **H, I** sternal cones between coxae 4, subcaudal and sublateral views, respectively.

Length ca 29 mm, width of midbody pro- and metazona 2.2 and 3.3 mm, respectively. Coloration of alcohol material upon long-term preservation rather uniformly brown (Fig. 39) (vs very dark brown, as given in the original description (Attems 1930a)).

All other characters as in *Orthomorpha hydrobiologica*, except as follows.

Collum with three transverse rows of setae, 4+4 anterior, 2+2 intermediate, and 2+2 posterior setae; caudal corner of paraterga subrectangular, narrowly rounded (Fig. 39A & B). Postcollum metaterga with two transverse rows of setae traceable as insertion points: 2+2 in anterior (pre-sulcus) row, 4+4 in posterior (postsulcus) one, the latter borne on small tubercles. Paraterga strongly developed (Fig. 39A-G), mostly subhorizontal and lying below dorsum, on postcollum segments extending increasingly beyond rear tergal margin, always pointed, on segments 16–19 tips strongly curved mesad. Calluses on all paraterga delimited by a sulcus only dorsally, rather broad, especially so on pore-bearing segments. Paraterga 2 broad, anterior edge rounded, lateral edge with only one small incision near midway (Fig. 39A). Transverse sulcus complete on metaterga 5–18, shallow, not reaching bases of paraterga, ribbed at bottom, slightly sinuate anteromedially (Fig. 39A, C & F). Pleurosternal carinae complete crests only on segments 2–4 (Fig. 39B), each with an evident sharp denticle caudally, thereafter increasingly strongly reduced until segment 10.

Sterna sparsely setose, with neither modifications nor knobs between ♂ coxae 4 (Fig. 39H & I). Gonopod aperture damaged during removal of gonopods. Legs long and slender, midbody ones ca 1.3–1.4 times as long as body height, prefemora without modifications, tarsal brushes present until ♂ legs 10.

**Figure 40.**
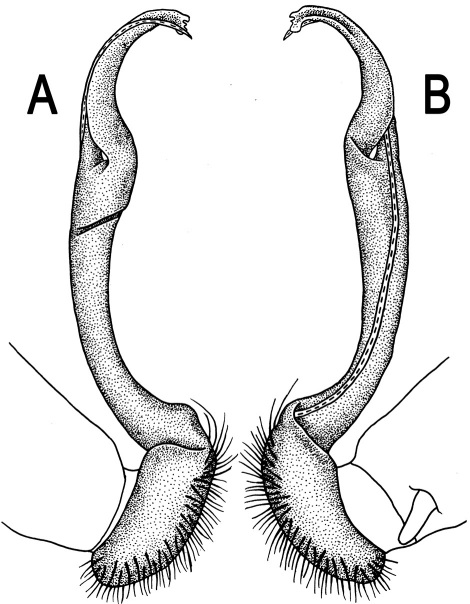
*Orthomorpha unicolor* Attems, 1930, ♂ holotype. **A, B** right gonopod, lateral and mesal views, respectively.

Gonopods (Fig. 40) with prefemur enlarged, about 2 times shorter than femorite + “postfemoral” part.

#### Remarks.

This variety was elevated to a full species by (Jeekel (1963, 1964, 1968), also treated as such by Hoffman (1973) and Golovatch (1998).

### Orthomorpha scabra

Jeekel, 1964

http://species-id.net/wiki/Orthomorpha_scabra

Figs 41 42

Pratinus granosus Attems 1953: 166 (D).
Orthomorpha granosa – Jeekel 1963: 265 (M).
Orthomorpha scabra Jeekel 1964: 361 (N, D).
Orthomorpha scabra – Jeekel 1968: 56 (M); Hoffman 1977: 700 (M); Golovatch 1998: 42 (M, D).

#### Lectotype.

♂ of *Pratinus granosus* (NHMW-3516), Vietnam, Lamdong Prov., Peak Lang Biang, 1938–1939, leg. C. Dawydoff.

Lectotype designation proposed herewith is necessary to ensure the species is based on a complete male coming from a certain locality, because (1) Attems (1953) had provided no information on the number of syntypes, and (2) he stated their provenance to have been both from Xieng Kunang, Luang Prabang Province, Laos and Peak Lang Biang, Lamdong Province, Vietnam.

#### Redescription.

**Figure 41.**
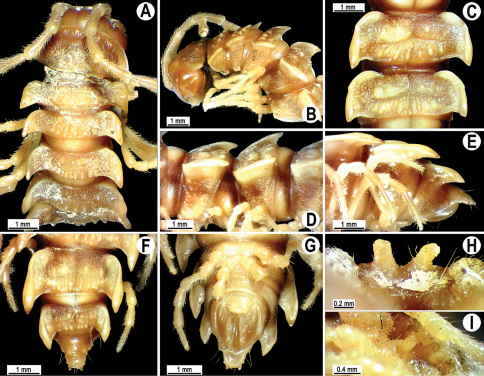
*Orthomorpha scabra* Jeekel, 1964, ♂ lectotype. **A, B** anterior part of body, dorsal and lateral views, respectively **C, D** segments 10 and 11, dorsal and lateral views, respectively **E-F** posterior part of body, lateral, dorsal and ventral views, respectively **H, I** sternal cones between coxae 4, subcaudal and sublateral views, respectively.

Length 41 mm, width of midbody pro- and metazona 3.1 and 4.4 mm, respectively (vs 38 mm in length and 4.6 mm in width, as given in the original description (Attems 1953)). Coloration of alcohol material upon long-term preservation rather uniformly light brown (Fig. 41) (vs black-brown with rather contrasting light brown to yellow-brown prozona, paraterga and venter, as well as red-brown legs, as given in the original description (Attems 1953)).

Head usual, clypeolabral region sparsely setose, surface of vertex smooth, epicranial suture very distinct. Antennae rather long and slender (Fig. 41A, B & J), extending behind body segment 3 dorsally. Head in width < collum < segments 3 and 4 < segment 2 < segments 5–17, gently and gradually tapering thereafter. Collum with three transverse rows of setae, 4+4 anterior, 2+2 intermediate, and 2+2 posterior setae, all borne on small knobs; caudal corner of paraterga subrectangular, nearly pointed (Fig. 41A & B). Tegument dull; metaterga coriaceous, rugulose, each postcollum one with two rows of evident tubercles, caudal row being more evident: 2+2 and 3+3 on metatergum 2, 3+2 and 4+3 on metatergum 3, 2–3+2–4 until metatergum 17, 1+2 and 2+3 on metaterga 18 and 19; prozona very finely shagreened, surface below paraterga microgranulate. Tergal setae abraded. Axial line visible, starting from metatergum 2. Paraterga very strongly developed (Fig. 41A-G), mostly slightly upturned and lying above dorsum, lying below dorsum only on segments 2 and 3, thin in lateral view, like blunt blades, a little thicker only on pore-bearing segments, on postcollum segments extending increasingly beyond rear tergal margin, all pointed. Calluses on paraterga 2 delimited by a sulcus only dorsally, on following paraterga both dorsally and ventrally, rather broad, especially so on pore-bearing segments. Paraterga 2 broad, anterior edge rounded, lateral edge with two small incisions in anterior half; posterior edge evidently concave (Fig. 41A & B). Paraterga 3 and 4 subequal, like subsequent paraterga, anterior edge broadly rounded, bordered and fused to callus, lateral edge with one small incision in anterior half. Ozopores evident, lateral, not sunken inside a groove, lying at about 1/3 of metazonital length. Transverse sulcus complete on metaterga 4–18, incomplete on metatergum 3, shallow, reaching bases of paraterga, smooth at bottom, slightly sinuate anteromedially (Fig. 41A, B, C, D & F). Stricture between pro- and metazona rather narrow, shallow, slightly ribbed at bottom down to base of paraterga. Pleurosternal carinae complete crests only on segments 2–4 (Fig. 41B, D & E), each with an evident sharp denticle caudally, thereafter increasingly strongly reduced until segment 11. Epiproct (Fig. 41E-G) conical, flattened dorsoventrally, apical papillae small; tip subtruncate; pre-apical papillae small, but visible, lying close to tip. Hypoproct (Fig. 41G) subtrapeziform, setiferous knobs at a subtruncate caudal margin small and well-separated.

Sterna densely setose, without modifications, but with two large, rounded, fully separated, setose cones between ♂ coxae 4 (Fig. 41H & I). A paramedian pair of conspicuous, high tubercles in front of gonopod aperture. Legs long and slender, midbody ones ca 1.3–1.4 as long as body height, prefemora without modifications, tarsal brushes present until ♂ legs 5.

**Figure 42.**
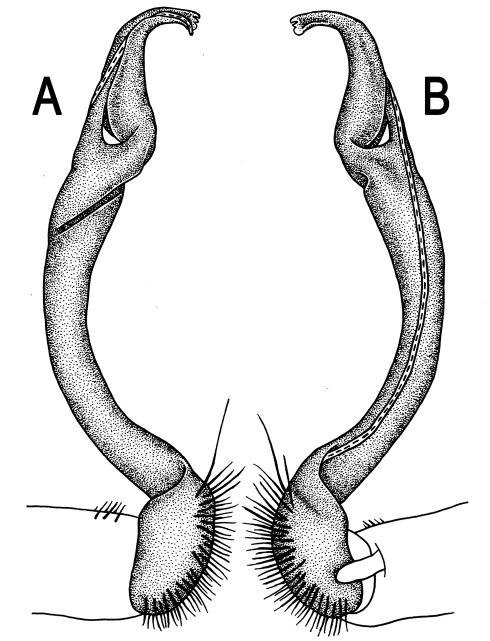
*Orthomorpha scabra* Jeekel, 1964, ♂ lectotype. **A, B** right gonopod, lateral and mesal views, respectively.

Gonopods (Fig. 42) simple. Coxa long and slender, with several setae distodorsally. Prefemur densely setose, about 2 times shorter than femorite + “postfemoral” part. Femorite very slender, evidently curved, slightly enlarged distad, “postfemoral” part demarcated by an oblique lateral sulcus; tip of solenophore small, trifid, with two subequal denticles (one terminal, the other middle) and a broader subterminal lobule.

#### Remark.

This species was renamed to avoid homonymy (Jeekel 1964).

### Orthomorpha rotundicollis

(Attems, 1937)

http://species-id.net/wiki/Orthomorpha_rotundicollis

**Figure 43.**
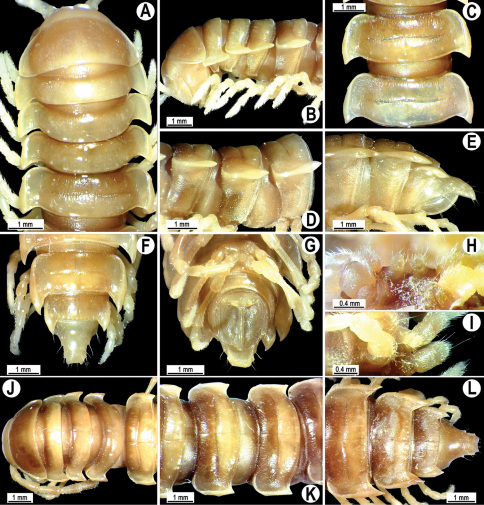
*Orthomorpha rotundicollis* Attems, 1937, ♂ lectotype (**A–I**), ♀ (**J–L**) of *Pratinus rotundicollis* Attems, 1937. **A, B, J** anterior part of body, dorsal, lateral and dorsal views, respectively **C, D, K** segments 10 and 11, dorsal, lateral and dorsal views, respectively **E–G, L** posterior part of body, lateral, dorsal, ventral and dorsal views, respectively **H, I** sternal cones between coxae 4, subcaudal and sublateral views, respectively.

Figs 43 [Fig F45] 46

Pratinus rotundicollis Attems 1937: 118 (D).
Pratinus rotundicollis – Attems 1938: 217 (D); 1953: 170 (R).
Pratinus tuberculatus Attems 1937: 119 (D), **syn. n.**
Pratinus tuberculatus – Attems 1938: 219 (D).
Orthomorpha rotundicollis – Jeekel 1963: 265 (M); 1964: 361 (M, D); 1968: 56 (M); Hoffman 1973: 363 (M); 1977: 700 (M); Golovatch 1998: 42 (M, D); Enghoff et al. 2004: 38 (M, R).
Orthomorpha tuberculata – Jeekel 1963: 265 (M); 1964: 361 (M, D); 1968: 56 (M); Hoffman 1977: 700 (M); Golovatch 1998: 42 (M, D); Enghoff et al. 2004: 39 (M, R).

#### Lectotype.

♂ of *Pratinus rotundicollis* (NHMW-3515), Vietnam, Lamdong Prov., Lang Biang, Arbre-Broyé, 1500 m, 01.02.1931, leg. C. Dawydoff.

#### Paralectotype.

1 ♂ of *Pratinus rotundicollis* (NHMW-3515), Vietnam, Lamdong Prov., Dalat, 1500 m, 02.1933, leg. C. Dawydoff.

#### Non-type material.

1 ♀ of *Pratinus rotundicollis* (NHMW-8000), Laos, Xiangkhoang Prov., Xiangkhoang Plateau, Xiang Kuang, no date, leg. C. Dawydoff, det. Attems.

#### Syntypes.

2 ♂, 1 ♀, 1 juv. of *Pratinus tuberculatus* (NHMW-3519), Vietnam, Lamdong Prov., Peak Lang Biang, Arbre-Broyé, 1500 m, 01.02.1931, leg. C. Dawydoff.

Lectotype designation proposed herewith is necessary to ensure the species is based on a complete male coming from a certain locality, because (1) (Attems (1937, 1938) had provided no information on the number of syntypes, and (2) he stated their provenance to have been both from Arbre-Broyé and Dalat, Vietnam.

#### Redescription.

Length 29–31 mm (♂), ca 31–35 mm (♀), width of midbody pro- and metazona 2.5–3.0 and 3.7–4.1 mm (♂), 3.4 and 4.5 mm (♀), respectively (vs 2.5 and 4.0 mm in width, as given in the available descriptions (Attems 1937, 1938)). Lectotype ca 31 mm long, 2.9 and 4.1 mm wide on midbody pro- and metazona, respectively. Coloration of alcohol material upon long-term preservation rather uniformly light grey-brown (Figs 43 & 45) (vs with a cingulate pattern of castaneous brown head and collum, as well as regions of pro- and metazona adjacent to stricture alternating with light brownish remaining parts, including antennae and legs, as given in the descriptions (Attems 1937, 1938)).

Head usual, clypeolabral region densely setose, surface of vertex smooth, epicranial suture distinct. Antennae rather long and slender (Figs 43J, 45B & J), extending behind body segment 4 (♂) or 3 (♀) dorsally. Head in width < collum < segments 3 and 4 < segment 2 < segments 5–17 (♂, ♀), gently and gradually tapering thereafter. Collum smooth, with three transverse rows of setae, 4+4 anterior, 2+2 intermediate, and 3+3 posterior setae; caudal corner of paraterga subrectangular, narrowly rounded (Figs 43A, B, J, 45A, B & J). Tegument smooth and shining; metaterga very faintly rugulose, each postcollum one with one caudal row of 3(4)+3(4) very small tubercles growing a little larger laterally, as well as with 2+2 either minute or fully obliterated knobs with insertion points of abraded setae in front row; prozona very finely shagreened, surface below paraterga finely microgranulate. Axial line visible, starting from collum. Paraterga very strongly developed (Figs 43A-G, J-L, 45A-G & J-L), all subhorizontal and lying below dorsum, thin in lateral view, like blunt blades, a little thicker only on pore-bearing segments, on postcollum segments extending increasingly beyond rear tergal margin, nearly pointed to pointed, caudal tip on paraterga 16–19 evidently curved mesad. Calluses on delimited by a sulcus only dorsally, rather narrow. Paraterga 2 broad, anterior edge angulate, lateral edge with three small incisions in anterior half; posterior edge evidently concave (Figs 43A, B, J, 45A, B & J). Paraterga 3 and 4 subequal, like subsequent paraterga, anterior edge broadly rounded, bordered and fused to callus, lateral edge with two small incisions in anterior half on poreless segments, with only one incision near front 1/3 on pore-bearing ones. Ozopores evident, lateral, lying inside an ovoid groove at about 1/4 metazonital length. Transverse sulcus complete on metaterga 5–18, incomplete on metatergum 4, shallow, not reaching bases of paraterga, beaded at bottom, slightly sinuate anteromedially (Figs 43A- D, F, J-L, 45A-F & J-L). Stricture between pro- and metazona rather narrow, deep, ribbed at bottom down to base of paraterga. Pleurosternal carinae complete crests only on segments 2–4 (Figs 43B, D, E, J-K, 45A-F & J-L), each with an evident sharp denticle caudally, thereafter increasingly strongly reduced until segment 10 (♂, ♀). Epiproct (Figs 43E-G, 45E-G) conical, flattened dorsoventrally, apical papillae small; tip subtruncate; pre-apical papillae small, but visible, lying close to tip. Hypoproct (Figs 43G, 45G) roundly subtriangular, setiferous knobs at caudal margin small and well-separated.

Sterna sparsely setose, without modifications, but with two rather large, rounded, fully separated, but nearly contiguous, setose cones between ♂ coxae 4 (Figs 43H, I, 45H & I). A paramedian pair of small tubercles in front of gonopod aperture. Legs long and slender, midbody ones ca 1.2–1.3 (♂) or 0.8–0.9 (♀) as long as midbody height, prefemora without modifications, tarsal brushes present until ♂ legs 4.

**Figure 44.**
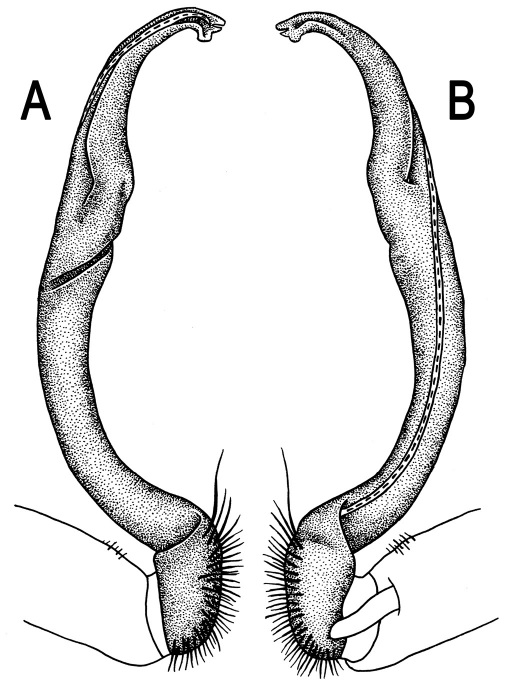
*Orthomorpha rotundicollis* Attems, 1937, ♂ lectotype of *Pratinus rotundicollis* Attems, 1937. **A, B** right gonopod, lateral and mesal views, respectively.

Gonopods (Figs 44 & 46) simple. Coxa long and slender, with several setae distodorsally. Prefemur densely setose, nearly 3 times shorter than femorite + “postfemoral” part. Femorite very slender, evidently curved, very slightly enlarged distad, “postfemoral” part demarcated by an oblique lateral sulcus; tip of solenophore small, trifid, with two subequal denticles (one terminal, the other middle) and a broader subterminal lobule.

**Figure 45. F45:**
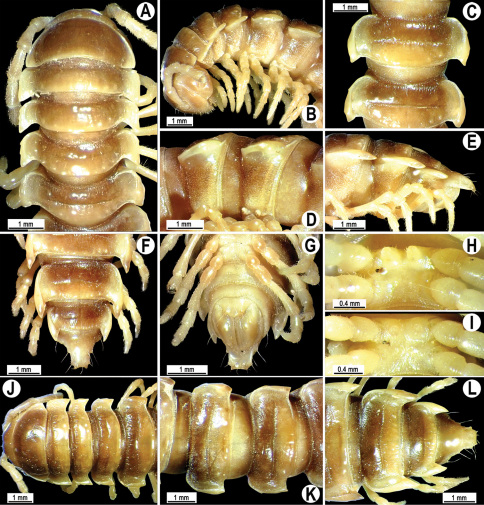
*Orthomorpha rotundicollis* Attems, 1937, ♂ syntype (**A–I**), ♀ syntype (**J–L**) of *Pratinus tuberculatus* Attems 1937. **A, B, J** anterior part of body, dorsal, lateral and dorsal views, respectively **C, D** segments 11 and 12, dorsal and lateral veiws, respectively **K** segments 10 and 11, dorsal view **E–F, L** posterior part of body, lateral, dorsal, ventral and dorsal views, respectively **H, I** sternal cones between coxae 4, subcaudal and sublateral views, respectively.

**Figure 46.**
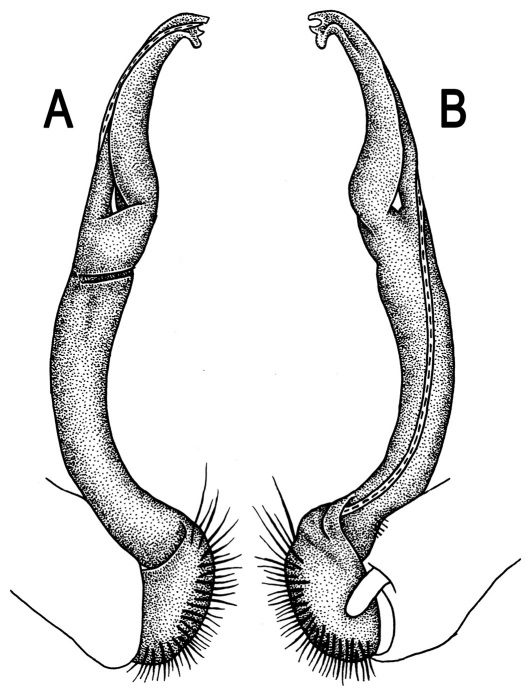
*Orthomorpha rotundicollis* Attems, 1937, ♂ syntype of *Pratinus tuberculatus* Attems 1937. **A, B** right gonopod, lateral and mesal views, respectively.

#### Remark.

This species is known from Lamdong Province, southern Vietnam and Luang Prabang Province, Laos (Attems 1938, 1953). The type locality of both *Orthomorpha rotundicollis* and *Orthomorpha tuberculata* being exactly the same (Arbre-Broyé at Peak Lang Biang), and the differences between them so minor, rather to be treated as infraspecific variations, we are inclined to formally synonymize these species. Indeed, distinctions are observed only in *Orthomorpha rotundicollis* showing a slightly more rounded caudal corner of the paraterga on the collum (vs slightly more angular in *Orthomorpha tuberculata*), in the front row of metatergal setae (= insertion points) borne on virtually fully obliterated knobs (vs on minute knobs in *Orthomorpha tuberculata*), and in the apical papillae on the epiproct directed more caudally than ventrally (vs more ventrally than caudally in *Orthomorpha tuberculata*) (Attems 1937, 1938, 1953). We can also add another two differences: paraterga 18 and 19 seem to be a little shorter in *Orthomorpha rotundicollis* than in *Orthomorpha tuberculata*, and the caudal row of tubercles on a few caudalmost metaterga demonstrating a pattern of 3+4 or 4+3 in *Orthomorpha tuberculata* (vs invariably 3+3 in *Orthomorpha rotundicollis*).

The above is a combined redescription, based on material of both nominate species.

### Orthomorpha cambodjana

(Attems, 1953)

http://species-id.net/wiki/Orthomorpha_cambodjana

Figs 47 48

Pratinus cambodjanus Attems 1953: 168 (D).
Orthomorpha cambodjana – Jeekel 1963: 265 (M); 1964: 361 (M, D); 1968: 56 (M); Hoffman 1977: 700 (M); Golovatch 1998: 42 (M, D).

#### Lectotype.

♂ (NHMW-3501), Cambodia, Kampot Prov., Ream, Sre-Umbell, 1938–39, leg. C. Dawydoff.

#### Paralectotype.

1 ♀ (NHMW-3501), same locality, together with lectotype.

Lectotype designation proposed herewith is necessary to ensure the species is based on a complete male.

#### Redescription.

**Figure 47.**
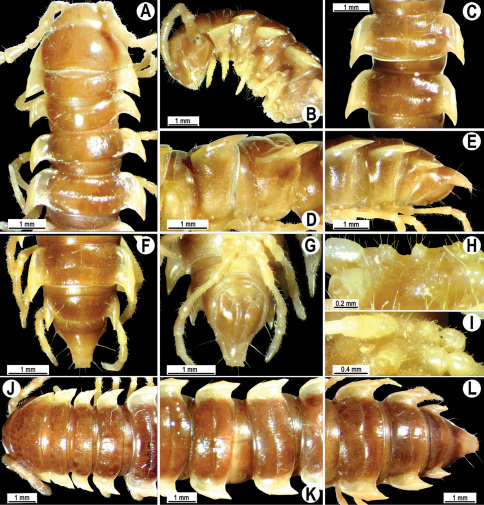
*Orthomorpha cambodijana* (Attems, 1953), ♂ lectotype (**A–I**), ♀ paralectotype (**J–L**). **A, B, J** anterior part of body, dorsal, lateral and dorsal views, respectively **C, K** segments 10 and 11, dorsal view **D** segments 11 and 12 lateral view **E–F, L** posterior part of body, lateral, dorsal, ventral and dorsal views, respectively **H, I** sternal cones between coxae 4, subcaudal and sublateral views, respectively.

Length ca 31 mm (♂), 32 mm (♀), width of midbody pro- and metazona 2.2 and 3.4 mm (♂), 3.1 and 4.3 mm (♀), respectively (vs 3.2–4.0 mm in width, as given in the original description (Attems 1953)). Coloration of alcohol material upon long-term preservation rather uniformly brown (Fig. 47) (vs dark castaneous with venter, legs and paraterga yellow-brown, and antennae light brown, as given in the original description (Attems 1953)).

Head usual, clypeolabral region densely setose, surface of vertex smooth, with 1+1 setae flanking a distinct epicranial suture. Antennae rather long and slender (Fig. 47B & J), extending behind segment 3 (♂, ♀) dorsally. Head in width < collum < segments 3 and 4 < segment 2 < segments 5–16 (♂, ♀), gently and gradually tapering thereafter. Collum smooth, with three transverse rows of setae, 4+4 anterior, 2+2 intermediate, and 3+3 posterior setae; caudal corner of paraterga acutangular (ca 70°), nearly pointed (Fig. 47A & J). Tegument smooth and shining; metaterga very faintly rugulose, each postcollum one with two rows of mostly abraded, medium-sized setae: 3+3 in front row and 3+3 or, on segments 12–19, 4+4 in caudal row; prozona very finely shagreened, surface below paraterga finely microgranulate. Axial line faint, but visible, starting from collum. Paraterga very strongly developed (Fig. 47A-G & J-L), set high (at ca 1/4 metazonital height), mostly slightly upturned, all lying below dorsum, thin in lateral view, like blunt blades, a little thicker only on pore-bearing segments, on postcollum segments extending increasingly beyond rear tergal margin, nearly pointed to pointed, caudal tip on paraterga 16–19 evidently curved mesad. Calluses on paraterga 2 delimited by a sulcus only dorsally, following paraterga by a sulcus both dorsally and ventrally, rather broad. Paraterga 2 broad, anterior edge angulate, lateral edge with three small incisions in anterior half; posterior edge evidently concave (Fig. 47A, B & J). Paraterga 3 and 4 subequal, like subsequent paraterga, anterior edge broadly rounded, bordered and fused to callus, lateral edge with two small incisions. Ozopores evident, lateral, lying inside an ovoid groove at about 1/4 metazonital length. Transverse sulcus complete on metaterga 5–18, incomplete on metatergum 4, shallow, not reaching bases of paraterga, beaded at bottom, slightly sinuate anteromedially (Fig. 47A, C, F & J-L). Stricture between pro- and metazona narrow, deep, ribbed at bottom down to base of paraterga. Pleurosternal carinae complete crests only on segments 2–4 (Fig. 47B, D & E), each with an evident sharp denticle caudally, thereafter increasingly strongly reduced until segment 14 (♂) or 11 (♀). Epiproct (Fig. 47E-G & L) conical, flattened dorsoventrally, apical papillae small, dentiform, directed caudoventrally; tip subtruncate; pre-apical papillae small, lying close to tip. Hypoproct (Fig. 47G) roundly subtrapeziform, setiferous knobs at caudal margin large and well-separated.

Sterna sparsely setose, without modifications, but with two large, rounded, fully separated, setose cones between ♂ coxae 4 (Fig. 47H & I). A paramedian pair of small tubercles in front of gonopod aperture. Legs long and slender, midbody ones ca 1.2–1.3 (♂) or 0.8–0.9 (♀) as long as body height, prefemora without modifications, tarsal brushes present until ♂ legs 5.

**Figure 48.**
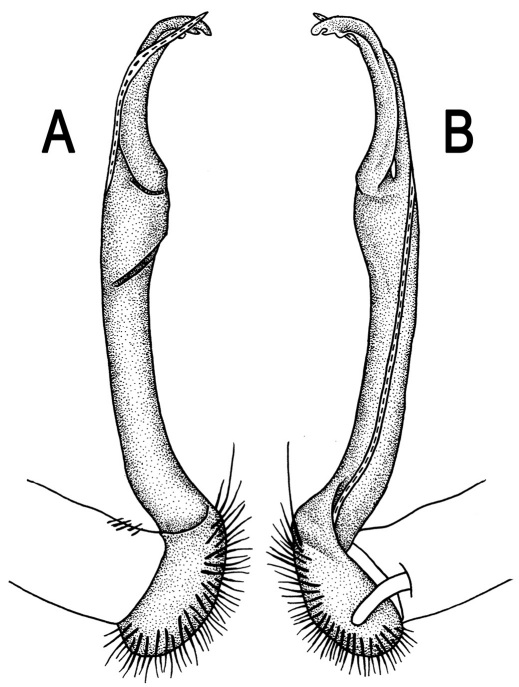
*Orthomorpha cambodijana* (Attems, 1953), ♂ lectotype. **A, B** right gonopod, lateral and mesal views, respectively.

Gonopods (Fig. 48) simple. Coxa long and slender, with several setae distodorsally. Prefemur densely setose, nearly 3 times shorter than femorite + “postfemoral” part. Femorite very slender, slightly curved, very slightly enlarged distad, “postfemoral” part demarcated by an oblique lateral sulcus; tip of solenophore small, trifid, with two denticles (terminal tooth larger, middle one smaller) and a small subterminal lobule.

### Orthomorpha tenuipes

(Attems, 1898)

http://species-id.net/wiki/Orthomorpha_tenuipes

Figs 49 50

Prionopeltis tenuipes Attems 1898: 356 (D).
Prionopeltis tenuipes – Attems 1914: 204 (M).
Pratinus tenuipes – Attems 1937: 117 (D).
Orthomorpha tenuipes – Jeekel 1963: 265 (M); 1964: 361 (M, D); 1968: 45 (M); Golovatch 1998: 42 (D, M).

#### Lectotype.

♂ (NHMW-3518), Indonesia, eastern Java, Tengger Mountains, no date, leg. H. Fruhsdorfer.

#### Non-type material.

1 ♂, 6 ♀ (NHMW-7998); 1 ♂, 1 ♀ (NHMW-7999); 8 ♂, 15 ♀ (NHMW-8003), Indonesia, Java, Tjibodas, 1897, leg. H. A. Möller, det. Attems. 4 ♂, 1 ♀ (NHMW-8002), Indonesia, Java, no date, leg. T. H. Adensamer, det. C. Attems.

Lectotype designation proposed herewith is necessary to ensure the species is based on a complete male, because the type series was stated to have been shared between the collections of the Berlin and Vienna museums (Attems 1898).

#### Redescription.

**Figure 49.**
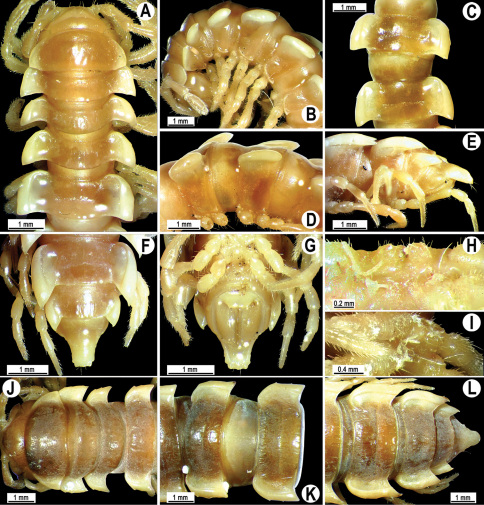
*Orthomorpha tenuipes* (Attems, 1898), ♂ lectotype (**A–G**), ♂ from Tjibodas (**H, I**), ♀ from Tjibodas (**J–L**). **A, B, J** anterior part of body, dorsal, lateral and dorsal views, respectively **C, D, K** segments 10 and 11, dorsal, lateral and dorsal views, respectively **E–F, L** posterior part of body, lateral, dorsal, ventral and dorsal views, respectively **H, I** sternal cones between coxae 4, subcaudal and sublateral views, respectively.

Length 24–28 mm (♂), 29–33 mm (♀), width of midbody pro- and metazona 2.6–2.8 and 3.4–4.1 mm (♂), 3.3–3.7 and 4.5–5.1 mm (♀), respectively (vs 40 mm in length and 4.0 mm in width, as given in the available descriptions (Attems 1898, 1937)). Lectotype ca 26 mm long, 1.7 and 3.4 mm wide on midbody pro- and metazona, respectively. Coloration of alcohol material upon long-term preservation mostly grey-brown (Fig. 49) with contrasting yellowish paraterga and epiproct, and light brown venter and legs (vs dark castaneous brown with paraterga and epiproct yellow, and venter and legs light red-brown, as given in the descriptions (Attems 1898, 1937)).

Head usual, clypeolabral region sparsely setose, surface of vertex smooth and bare, epicranial suture distinct. Antennae rather long and slender (Fig. 49B & J), extending behind almost to end of segment 3 (♂) or reaching segment 3 (♀) dorsally. Head in width < collum < segment 3 < 2 < 4 < 5–16 (♂), or head < collum < segments 3 and 4 < 2 < 5–16 (♀), gently and gradually tapering thereafter. Collum smooth, with three transverse rows of setae, 4+4 anterior, 2+2 intermediate, and 3+3 posterior setae; caudal corner of paraterga subrectangular, narrowly rounded (Fig. 49A, B & J). Tegument smooth and shining; metaterga very faintly rugulose, each postcollum one with two rows of fully abraded setae traceable only as insertion points: 2+2 in front row and 3+3 or, on several caudal segments, 4+4, in caudal row, these borne on indistinct, truncate, very low tubercles; prozona very finely shagreened, surface below paraterga finely microgranulate. Axial line faint, but visible, starting from collum. Paraterga very strongly developed (Fig. 49A-G & J-L), set high (at ca 1/4 metazonital height), subhorizontal to slightly upturned, lying below dorsum on segments 2–6 and 19, above dorsum on segments 7–18, rather thick in lateral view, a little thicker on pore-bearing segments, on postcollum segments extending increasingly beyond rear tergal margin starting only from segment 5 or 6 (♂) or midbody segments (♀), narrowly rounded to pointed, caudal tip on paraterga 16–19 evidently curved mesad. Calluses on paraterga delimited by a sulcus only dorsally, broad. Paraterga 2 broad, anterior edge broadly rounded, lateral edge with three minute incisions in anterior half; posterior edge evidently concave (Fig. 49A, B & J). Paraterga 3 and 4 subequal, like subsequent paraterga, anterior edge broadly rounded, bordered and fused to callus, lateral edge with two minute incisions. Ozopores not too evident, ventrolateral, not lying inside a groove, placed at about 1/3 metazonital length. Transverse sulcus complete on metaterga 5–18, incomplete on metatergum 4, shallow, not reaching bases of paraterga, beaded at bottom, slightly sinuate anteromedially (Fig. 49A, C, F & J-L). Stricture between pro- and metazona rather wide, shallow, ribbed at bottom down to base of paraterga. Pleurosternal carinae complete crests only on segments 2–7(8) (♂) or 2–4 (♀) (Fig. 49B, D & E), each with an evident sharp denticle caudally, thereafter increasingly strongly reduced until segment 16 (♂) or 15 (♀). Epiproct (Fig. 49E-G & L) conical, flattened dorsoventrally, apical papillae small, dentiform, directed caudoventrally; tip subtruncate; pre-apical papillae small, lying close to tip. Hypoproct (Fig. 49G) subtrapeziform, setiferous knobs at caudal margin small and well-separated.

Sterna sparsely setose, without modifications, but with two small, rounded, fully separated, but subcontiguous, setose cones between ♂ coxae 4 (Fig. 49H & I). A paramedian pair of evident tubercles in front of gonopod aperture. Legs long and slender, midbody ones ca 1.2–1.4 (♂) or 0.8–1.0 (♀) as long as midbody height, prefemora without modifications, tarsal brushes present until ♂ legs 7.

**Figure 50.**
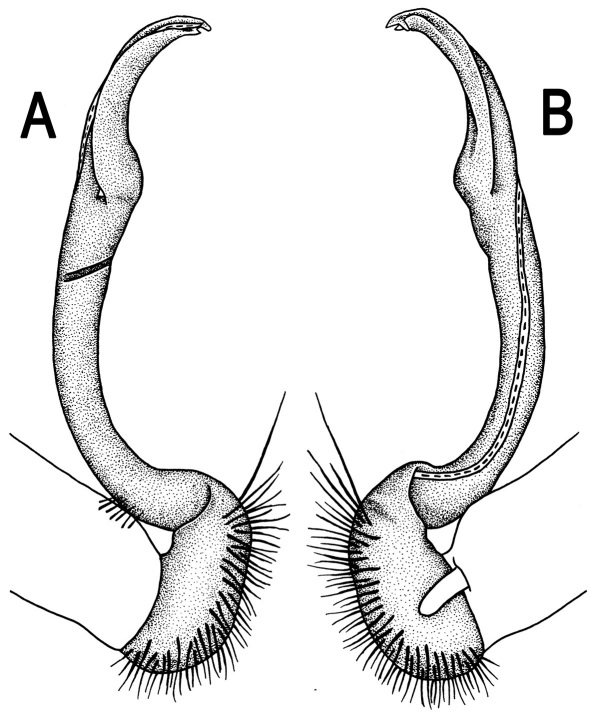
*Orthomorpha tenuipes* (Attems, 1898), ♂ lectotype. **A, B** right gonopod, lateral and mesal views, respectively.

Gonopods (Fig. 50) simple. Coxa long and slender, with several setae distodorsally. Prefemur rather large, densely setose, nearly 2 times shorter than femorite + ”postfemoral” part. Femorite very slender, evidently curved, nearly not enlarged distad, “postfemoral” part demarcated by an oblique lateral sulcus; tip of solenophore small, trifid, with two subequal denticles (terminal and subterminal) and a minute prong in-between.

### Orthomorpha glandulosa

(Attems, 1937)

http://species-id.net/wiki/Orthomorpha_glandulosa

Figs 51 52

Pratinus glandulosus Attems 1937: 119 (D).
Pratinus glandulosus – Attems 1938: 220 (D).
Orthomorpha glandulosa – Jeekel 1963: 265 (M); 1964: 361 (M, D); 1968: 56 (M); Hoffman 1977: 700 (M); Golovatch 1998: 42 (D, M); Enghoff et al. 2004: 38 (M, R).

#### Lectotype.

♂ (NHMW-3506), Vietnam, Hon Ba Island, Nhatrang, 06.1930, leg. C. Dawydoff.

#### Paralectotypes.

1 ♀ (NHMW-3506), same locality, together with lectotype. 1 ♀ (NHMW-3505), Vietnam, Darlac, frontier du Cambodge, 07.1930, leg. C. Dawydoff.

Lectotype designation proposed herewith is necessary to ensure the species is based on a complete male.

#### Redescription.

**Figure 51.**
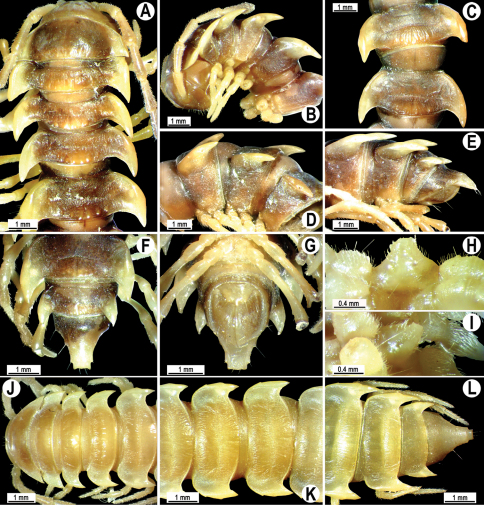
*Orthomorpha glandulosa* (Attems, 1937), ♂ lectotype (**A–I**), ♀ paralectotype (**J–L**). **A, B, J** anterior part of body, dorsal, lateral and dorsal views, respectively **C, D, K** segments 10 and 11, dorsal, lateral and dorsal views, respectively **E–G, L** posterior part of body, lateral, dorsal, ventral and dorsal views, respectively **H, I** sternal cones between coxae 4, subcaudal and sublateral views, respectively.

Length ca 38 mm (lectotype), 31–34 mm (♀), width of midbody pro- and metazona 3.4 and 5.0 mm (lectotype), 2.9–3.4 and 4.2–4.4 mm (♀), respectively (vs 3.0 and 5.0 in width, as given in the available descriptions (Attems 1937, 1938)). Coloration of alcohol material upon long-term preservation dark grey-brown (Fig. 51) with contrasting pallid paraterga and epiproct, and light brown venter and legs (vs dark castaneous brown with paraterga and epiproct yellow, and venter and legs light red-brown, as given in the descriptions (Attems 1937, 1938)).

Head usual, clypeolabral region densely setose, surface of vertex smooth, with a few setae flanking a distinct epicranial suture. Antennae long and slender (Fig. 51A, B & J), extending behind segment 4 (♂) or surpassing segment 3 (♀) dorsally. Head in width < collum < segment 2 < 3 = 4 < 5–16 (♂), or head < collum < segments 3 and 4 < 2 < 5–16 (♀), gently and gradually tapering thereafter. Collum smooth, with three transverse rows of setae traceable only as insertion points, 4+4 anterior, 2+2 intermediate, and 3+3 posterior setae; caudal corner of paraterga acutangular (ca 75°), nearly pointed (Fig. 51A, B & J). Tegument poorly shining; metaterga coriaceous, rugulose, each postcollum one with two rows of fully abraded setae borne on minute tubercles growing increasingly strongly reduced towards epiproct: 2+2 in front row and 3+3 in caudal one; prozona very finely shagreened, surface below paraterga finely microgranulate. Axial line rather evident, starting from collum. Paraterga very strongly developed (Fig. 51A-G & J-L), set high (at ca 1/4 metazonital height), in ♂ evidently upturned, lying above dorsum on postcollum segments, in ♀ mostly below dorsum, rather thin in lateral view, a little thicker on pore-bearing segments, on postcollum segments extending increasingly beyond rear tergal margin, better so in ♂, nearly pointed to pointed, caudal tip on paraterga 16–19 evidently curved mesad. Calluses on paraterga 2 delimited by a sulcus only dorsally, on following paraterga both dorsally and ventrally, rather broad. Paraterga 2 broad, anterior edge angulate, lateral edge with two minute incisions in anterior 1/3; posterior edge evidently concave (Fig. 51A, B & J). Paraterga 3 and 4 subequal, like subsequent paraterga, anterior edge broadly rounded, bordered and fused to callus, lateral edge with one minute incision in front 1/3. Ozopores evident, lateral, lying inside an ovoid groove, placed at about 1/3 metazonital length. Transverse sulcus complete on metaterga 4–18, incomplete on metaterga 2 and 3 (♂), or incomplete on metatergum 4 and complete on metaterga 5–18 (♀), shallow, not reaching bases of paraterga, ribbed at bottom, slightly sinuate anteromedially (Fig. 51A, C, F & J-L). Stricture between pro- and metazona narrow, shallow, beaded at bottom down to base of paraterga. Pleurosternal carinae complete crests only on segments 2–4 (♂, ♀) (Fig. 51B, D & E), each with an evident sharp denticle caudally, thereafter increasingly strongly reduced until segment 10 (♂, ♀). Epiproct (Fig. 51E-G & L) conical, flattened dorsoventrally, apical papillae small, dentiform, directed caudoventrally; tip subtruncate; pre-apical papillae small, lying close to tip. Hypoproct (Fig. 51G) roundly subtrapeziform, setiferous knobs at caudal margin small and well-separated.

Sterna sparsely setose, without modifications, but with a large, central, setose cone between ♂ coxae 4 (Fig. 51H & I). A paramedian pair of tubercles in front of gonopod aperture absent. Legs long and slender, midbody ones ca 1.3–1.4 (♂) or 1.2–1.3 (♀) as long as body height, prefemora without modifications, tarsal brushes present until ♂ legs 5.

**Figure 52.**
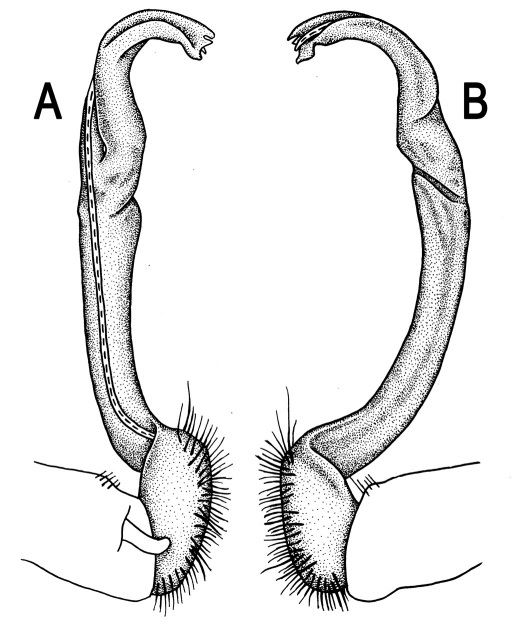
*Orthomorpha glandulosa* (Attems, 1937), ♂ lectotype. **A, B** left gonopod, mesal and lateral views, respectively.

Gonopods (Fig. 52) simple. Coxa long and slender, with several setae distodorsally. Prefemur rather large, densely setose, more than 2 times shorter than femorite + “postfemoral” part. Femorite very slender, evidently curved, not enlarged distad, “postfemoral” part demarcated by an oblique lateral sulcus; tip of solenophore small, trifid, with two subequal denticles (terminal and middle) and a larger subterminal lobule.

### Orthomorpha zehntneri

Carl, 1902

http://species-id.net/wiki/Orthomorpha_zehntneri

Figs 53 54

Orthomorpha Zehntneri Carl 1902: 584 (D).
Orthomorpha Zehntneri – Attems 1907: 83 (M); 1914: 192 (M, D).
Orthomorpha zehntneri – Attems 1930a: 127 (D); 1937: 73 (D); Jeekel 1963: 265 (M); 1964: 361 (M, D); 1968: 45 (M); 1980b: 325 (D); Golovatch 1998: 42 (D, M).

#### Syntypes.

2 ♂, 1 ♀ (MHNG), Indonesia, Java, no date, leg. L. Zehntner.

#### Redescription.

**Figure 53.**
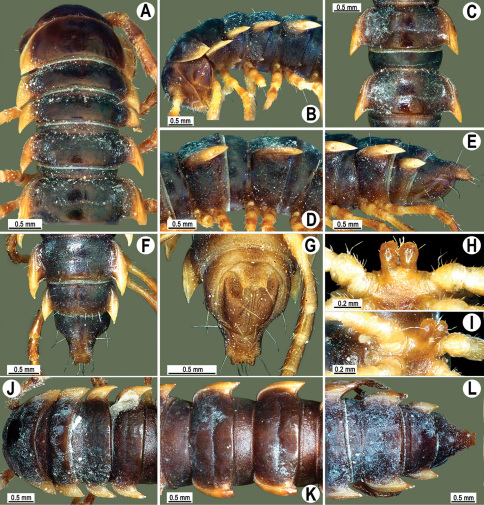
*Orthomorpha zehntneri* Carl, 1902, ♂ syntype (**A–I**), ♀ syntype (**J–L**). **A, B, J** anterior part of body, dorsal, lateral and dorsal views, respectively **C, D, K** segments 10 and 11, dorsal, lateral and dorsal views, respectively **E–G, L** posterior part of body, lateral, dorsal, ventral and dorsal views, respectively **H, I** sternal cones between coxae 4, subcaudal and sublateral views, respectively.

Length 19–22 (♂) or 24 mm (♀), width of midbody pro- and metazona 1.4–1.5 and 2.1–2.4 mm (♂), 2.0 and 2.6 mm (♀), respectively (vs 2.8 mm in width, as given in the original description (Carl 1902)). Coloration of alcohol material after long-term preservation dark castaneous brown with contrastingly light red-brown to pallid paraterga, venter, and basal podomeres; antenomere 7 blackish, tip of antennae pallid (Fig. 53).

Clypeolabral region densely setose, vertex sparsely so, epicranial suture distinct. Antennae moderately long, clavate (antennomere 6 broadest), extending behind body segment 3 (♂) or 2 (♀) dorsally (Fig. 53A & B). Head in width < collum < segment 2 < 3 and 4 < 5–16; thereafter body gently and gradually tapering. Collum semi-lunar, with three transverse rows of setae, 4+4 anterior, 2+2 intermediate and 3+3 posterior; caudal corner of paraterga pointed, slightly declined ventrally and continuing collum’s convexity (Fig. 53B); paraterga subrectangular, slightly surpassing rear tergal margin (Fig. 53A & J). Tegument smooth and shining, prozona very finely shagreened, metaterga leathery, faintly rugulose, below paraterga microgranular, faintly rugulose. Metaterga 2–19 with an anterior transverse row of 2+2, mostly abraded setae; caudal row barely traceable as 3+3 insertion points. Tergal setae simple, rather long, about 1/3 metatergal length. Axial line faint, starting from collum. Paraterga very well developed (Fig. 53A-G & J-L), especially well so in ♂, all lying below dorsum (at about 1/4 midbody height), subhorizontal, in lateral view modestly enlarged on pore-bearing segments, thinner on poreless ones; shoulders always present, mostly regularly rounded and narrowly bordered, fused to callus; caudal corner of all paraterga pointed, beak-like, extending increasingly beyond rear tergal margin, best developed and slightly curved mesad on segments 15–19. Calluses delimited by a sulcus both dorsally and ventrally, with three small, but evident lateral denticles on callus 2, with one or two similar, often setigerous incisions in front of following poreless segments, and with one incision on pore-bearing ones. Posterior edge of paraterga evidently concave, especially strongly so on segments 16–19. Ozopores evident, lateral, lying in an ovoid groove at about 1/4 in front of caudal corner. Transverse sulcus complete on metaterga 5–17, incomplete on segments 4 and 18, narrow, shallow, reaching bases of paraterga, finely beaded at bottom, better developed in ♂ (Fig. 53A, C, F & J-L). Stricture between pro- and metazona narrow and rather shallow, evidently beaded at bottom down to base of paraterga (Fig. 53A-G & J-L). Pleurosternal carinae complete crests with a sharp caudal tooth on segments 2 and 3, thereafter like increasingly poorly developed, flat ridges with small caudal teeth until segment 8, further on caudally only like an increasingly small, sharp, caudal tooth until segment 16 (♂, ♀) (Fig. 53B, D & E). Epiproct (Fig. 53E-G & L) conical, inflated dorsoventrally, with two evident apical papillae directed ventrocaudally, subtruncate at tip; pre-apical papillae evident, lying close to tip. Hypoproct (Fig. 53G) subtrapeziform, slightly rounded at caudal margin, setiferous knobs at caudal edge large and well-separated.

Sterna sparsely setose, without modifications; cross-impressions shallow; with a paramedian pair of very evident, anteroventrally directed lobe deeply, but incompletely split into two between ♂ coxae 4 (Fig. 53H & I). A paramedian pair of small tubercles in front of gonopod aperture. Legs long and slender, midbody ones ca 1.3–1.5 (♂) or 0.9–1.1 times (♀) as long as body height, prefemora without modifications, ♂ tarsal brushes present until legs of segment 16.

**Figure 54.**
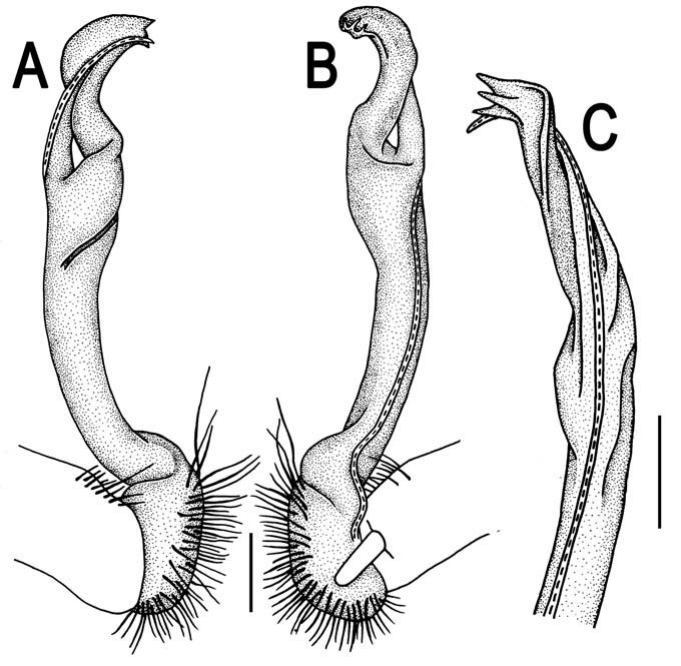
*Orthomorpha zehntneri* Carl, 1902, ♂ syntype. **A–C** right gonopod, lateral, mesal and subcaudal views, respectively.Scale bar: 0.2 mm.

Gonopods (Fig. 54) simple. Coxa long and slender, with numerous strong setae distodorsally. Prefemur densely setose, almost 3 times shorter than femorite + “postfemoral” part. Femorite slender, slightly curved and rather evidently enlarged distally, with a “postfemoral” part demarcated by an oblique lateral sulcus; solenophore with lamina lateralis much smaller than lamina medialis, the former slightly helicoid, forming a tridentate and mesally curved tip; both terminal and subterminal denticles/spikes subequal, acute and narrow, middle denticle nearly as long or shorter; solenomere long and flagelliform.

#### Remarks.

The above redescription is meant to augment the fairly complete one by Jeekel (1980b) which was also based on syntypes.

### Orthomorpha fluminoris

Hoffman, 1977

http://species-id.net/wiki/Orthomorpha_fluminoris

Figs 55 56

Orthomorpha fluminoris Hoffman 1977: 701 (D).
Orthomorpha fluminoris – Golovatch 1998: 42 (D, M).

#### Holotype.

♂ (MHNG), Malaysia, environs of Kuala Lumpur, Batu Caves, 24.02.1975, leg. P. Strinati.

#### Redescription.

**Figure 55.**
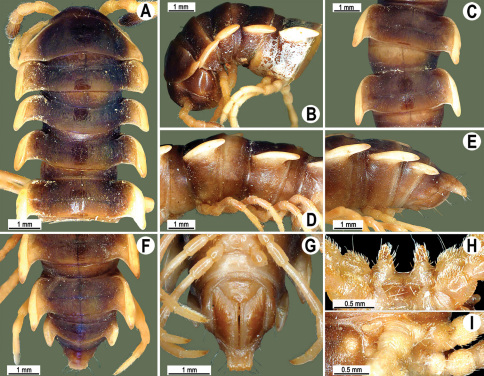
*Orthomorpha fluminoris* Hoffman, 1977, ♂ holotype. **A, B** anterior part of body, dorsal and lateral views, respectively **C, D** segments 10 and 11, dorsal and lateral views, respectively **E–G** posterior part of body, lateral, dorsal and ventral views, respectively **H, I** sternal cones between coxae 4, subcaudal and sublateral views, respectively.

Length 39 mm, width of midbody pro- and metazona 2.9 and 4.4 mm, respectively. Coloration of alcohol material upon long-term preservation dark castaneous brown with contrasting yellowish paraterga; venter, legs yellownish and tip of epiproct light red-brown, antennae also light red-brown, but antenomere 6 slightly infuscate, brownish, antenomere 7 dark brown, and tip of antennae pallid (Fig. 55).

Clypeolabral region densely setose, vertex sparsely so, epicranial suture distinct. Antennae rather short, clavate (antennomere 6 broadest), extending behind body segment 2 dorsally. Head in width < collum < segment 2 < 3 < 4 < 5–16; thereafter body gently and gradually tapering. Collum with three transverse rows of setae, 4+4 anterior, 2+2 intermediate, and 3+3 posterior; caudal corner of paraterga nearly pointed, slightly declined ventrally and continuing collum’s convexity (Fig. 55A & B); paraterga acutangular (ca 50°), slightly extending behind tergal margin, posterior edge slightly concave (Fig. 55A & B). Tegument smooth and poorly shining, prozona very finely shagreened, metazona leathery, rugulose, below paraterga microgranular. Metaterga 2–19 with two transverse rows of setae: 2+2 in anterior (pre-sulcus) row and 3+3 in posterior (postsulcus) one, all mostly abraded, but still traceable as insertion points. Tergal setae simple, moderately long, about 1/4 metatergal length. Axial line traceable. Paraterga very well developed (Fig. 55A-G), all lying below dorsum (at about 1/4 body height), slightly upturned to subhorizontal, in lateral view moderately strongly enlarged on pore-bearing segments, thinner on poreless ones; shoulders always present, broadly rounded and narrowly bordered, fused to callus; anterior edge of paraterga 2 straight, following paraterga rounded, caudal corner of all paraterga rounded, extending invariably and increasingly beyond rear tergal margin. Calluses delimited by a sulcus both dorsally and ventrally, rather broad. Posterior edge of paraterga evidently concave, especially strongly so on segments 16–19. Ozopores evident, lateral, lying in an ovoid groove at about 1/4 in front of caudal corner. Transverse sulcus complete on metaterga 5–18, incomplete on segments 4 and 19, very narrow, shallow, not reaching bases of paraterga, very finely ribbed at bottom (Fig. 55A, C & F). Stricture between pro- and metazona narrow and shallow, faintly beaded at bottom down to base of paraterga (Fig. 55A-F). Pleurosternal carinae complete crests with a sharp caudal tooth on segments 2–4, a low and interrupted swelling supplied with a small tooth caudally on segments 5–7, retained only as small denticles on segments 8–14, thereafter missing (Fig. 55B, D & E). Epiproct (Fig. 55E-G) conical, flattened dorsoventrally, with two small apical papillae directed ventrocaudally, subtruncate at tip; pre-apical papillae small, lying close to tip. Hypoproct (Fig. 55G) nearly semi-circular, caudal tip very broadly rounded, setiferous knobs at caudal edge very small and well-separated.

Sterna sparsely setose, without modifications; cross-impressions shallow; a pair of strongly separated, anteroventrally directed, narrowly rounded, setose cones between ♂ coxae 4 (Fig. 55H & I). A paramedian pair of small tubercles in front of gonopod aperture. Legs long and slender, midbody ones ca 1.3–1.5 times as long as body height, prefemora without modifications, ♂ tarsal brushes present until on legs of segment 17.

**Figure 56.**
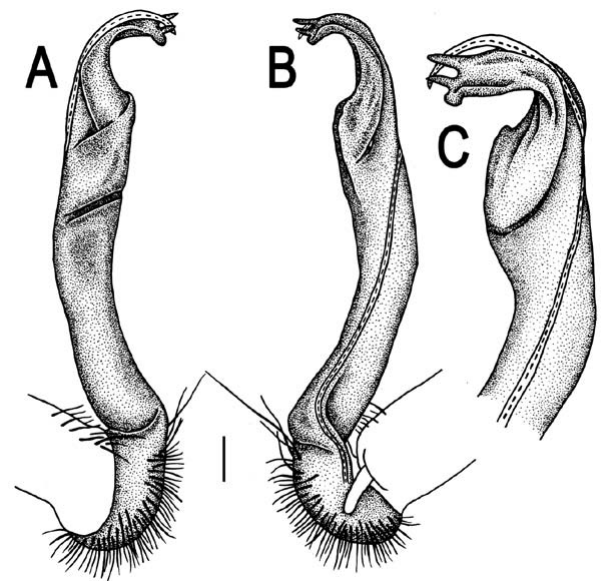
*Orthomorpha fluminoris* Hoffman, 1977, ♂ holotype. **A–C** right gonopod, lateral, mesal and subcaudal views, respectively. Scale bar: 0.2 mm.

Gonopods (Fig. 56) a little more complex. Coxa long and slender, with several strong setae distodorsally. Prefemur densely setose, about half the length of femorite + “postfemoral” part. Femorite slender, slightly curved and nearly not enlarged distad, with a small, but conspicuous ventral knob near base of lamina lateralis, with a “postfemoral” part demarcated by a distinct, oblique, lateral sulcus; solenophore with a tridentate tip, middle denticle shorter than terminal tooth, but longer than a small subterminal lobule; solenomere long, flagelliform, a short tip exposed.

#### Remarks.

The original description is fairly complete, also illustrated in due detail (Hoffman 1973). The above redescription, also based on the holotype, is only meant to bring it to our standard and to present additional illustrations.

### Orthomorpha murphyi

Hoffman, 1973

http://species-id.net/wiki/Orthomorpha_murphyi

**Figure 57.**
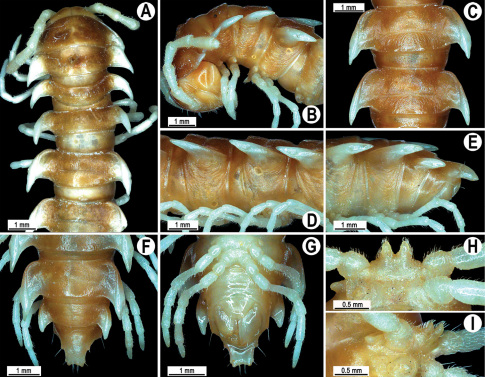
*Orthomorpha murphyi* Hoffman, 1973, ♂ holotype. **A, B** anterior part of body, dorsal and lateral views, respectively **C, D** segments 10 and 11, dorsal and lateral views, respectively **E–F** posterior part of body, lateral, dorsal and ventral views, respectively **H, I** sternal cones between coxae 4, subcaudal and sublateral views, respectively.

Figs 57 [Fig F58] [Fig F59] [Fig F60] 61

Orthomorpha murphyi Hoffman 1973: 636 (D).
Orthomorpha murphyi – Hoffman 1977: 700 (M, D); Golovatch 1998: 42 (M, D).

#### Holotype.

♂(NHML), Singapore, Mile 8, Old Upper Thomson Road, 30.12.1962, leg. Kok Oi Yee.

#### Non-types.

1 ♂, 5 ♀ (CUMZ), Singapore, Central Region, Nature Reserve, Bukit Timah, 1°35'18"N, 104°18'0"E, 25.12.2009, leg. C. Sutcharit.

#### Descriptive notes.

Length 34–35 (♂) or 3.3–3.6 mm (♀), width of midbody pro- and metazona 2.5–2.6 and 3.9–4.4 mm (♂), 3.4–3.6, and 4.8–4.9 mm (♀), respectively.

**Figure 58. F58:**
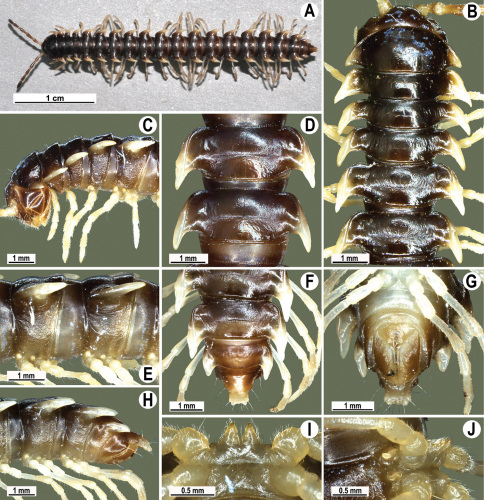
*Orthomorpha murphyi* Hoffman, 1973, ♂. **A** habitus, live coloration **B, C** anterior part of body, dorsal and lateral views, respectively **D, E** segments 10 and 11, dorsal and lateral views, respectively **F, G, H** posterior part of body, dorsal, ventral and lateral views, respectively **I, J** sternal cones between coxae 4, subcaudal and sublateral views, respectively.

Coloration of live animals (Fig. 58A) uniformly blackish brown with only slightly lighter paraterga, venter and legs; coloration of alcohol material upon long-term preservation faded to a light brown (holotype) with contrasting pallid paraterga, venter, legs and antennae (Fig. 57); coloration of rather freshly alcohol-preserved material blackish brown with likewise strongly faded paraterga, venter and legs (Fig. 58B-H).

**Figure 59. F59:**
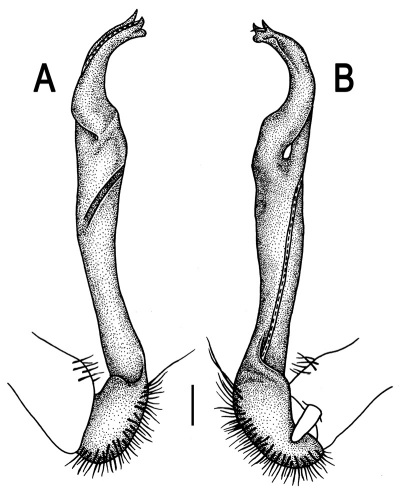
*Orthomorpha murphyi* Hoffman, 1973, ♂ holotype. **A, B** right gonopod, lateral and mesal views, respectively.Scale bar: 0.2 mm.

**Figure 60. F60:**
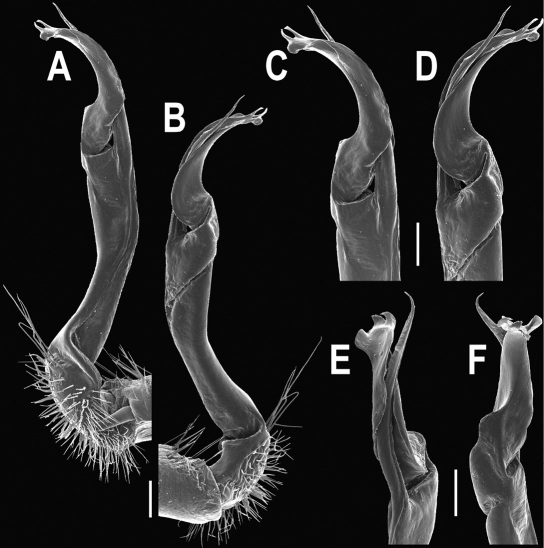
*Orthomorpha murphyi* Hoffman, 1973, ♂. **A, B** right gonopod, mesal and lateral views, respectively **C-F** distal part of right gonopod, mesal, lateral, subcaudal and suboral views, respectively. Scale bar: 0.2 mm.

**Figure 61.**
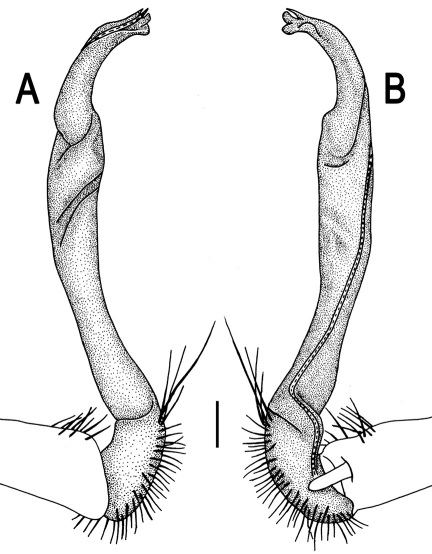
*Orthomorpha murphyi* Hoffman, 1973, ♂. **A, B** right gonopod, lateral and mesal views, respectively. Scale bar: 0.2 mm.

#### Remarks.

The original description of this species was so complete and detailed (Hoffman 1973) that no redescription seems to be necessary. Instead, we only provide new illustrations (Figs 59–61) showing the coloration, structural details, and gonopod conformation of *Orthomorpha murphyi*, based both on the holotype and new samples.

### Orthomorpha melischi

Golovatch, 1997

http://species-id.net/wiki/Orthomorpha_melischi

Figs 62 63

Orthomorpha melischi Golovatch 1997b: 77 (D).
Orthomorpha melischi – Golovatch 1998: 42 (D, M).

#### Holotype.

♂(NHMW-3509), Indonesia, Sumatra, Lampung Prov., Way Kambas, forest road, ca 10 m, 26.12.1993, leg. R. Melisch.

#### Remarks.

**Figure 62.**
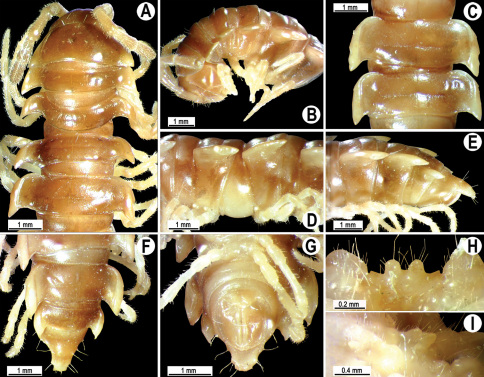
*Orthomorpha melischi* Golovatch, 1997, ♂ holotype. **A, B** anterior part of body, dorsal and lateral views, respectively **C, D** segments 10 and 11, dorsal and lateral views, respectively **E–G** posterior part of body, lateral, dorsal and ventral views, respectively **H, I** sternal cones between coxae 4, subcaudal and sublateral views, respectively.

**Figure 63.**
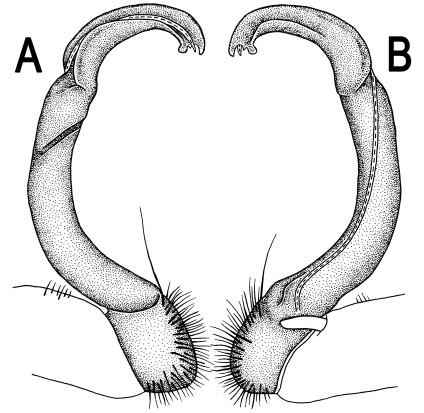
*Orthomorpha melischi* Golovatch, 1997, ♂ holotype. **A, B** right gonopod, lateral and mesal views, respectively.

The original description of this species was so complete and detailed (Golovatch 1997b) that no redescription seems to be necessary. Instead, we only provide new illustrations (Figs 62 & 63) showing the coloration, structural details and gonopod conformation of *Orthomorpha melischi*, based on the holotype.

### Orthomorpha pterygota

Golovatch, 1998

http://species-id.net/wiki/Orthomorpha_pterygota

**Figure 64.**
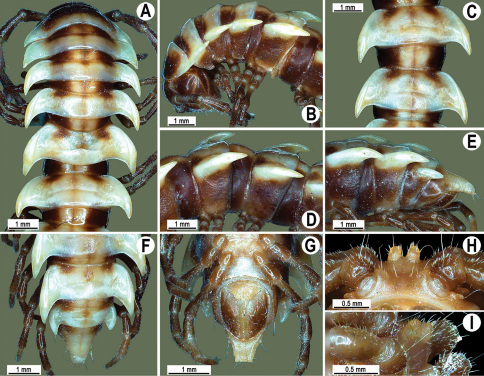
*Orthomorpha pterygota* Golovatch, 1998, ♂ holotype. **A, B** anterior part of body, dorsal and lateral views, respectively **C, D** segments 10 and 11, dorsal and lateral views, respectively **E–G** posterior part of body, lateral, dorsal and ventral views, respectively **H, I** sternal cones between coxae 4, subcaudal and sublateral views, respectively.

Figs 64 [Fig F65] [Fig F66] [Fig F67] 68

Orthomorpha pterygota Golovatch 1998: 41 (D).
Orthomorpha pterygota – Enghoff 2005: 98 (M, R); Decker 2010: 31 (R).

#### Holotype.

♂(ZMUC), Thailand, Krabi Prov., road between Krabi and Phuket, 10 km north of Krabi, 8°09'N, 98°50'E, lowland tropical rainforest, < 200 m, 13.10.1991, leg. M. Andersen, O. Martin & N. Scharff.

#### Other material examined.

1 ♂ (CUMZ), Thailand, Phuket Prov., Mueang Distr., Kathu Waterfall, ca 10 m, 7°55'56"N, 98°19'23"E, forest, 25.12.2009, leg. N. Likhitrakarn.2 ♂ (CUMZ), same Distr., Panwa Cape, 8°20'11"N, 98°41'36"E, 05.11.2007, leg. C. Sutcharit.

#### Descriptive notes.

Length 31–35 (♂), width of midbody pro- and metazona 2.2–3.0 and 4.0–4.6 mm (♂), respectively (vs 34–37 mm in length, 2.2–3.4 and 4.0–4.7 mm in width on midbody pro- and metazona, respectively, both in ♂ and ♀, as given in the original description (Golovatch 1998)).

**Figure 65. F65:**
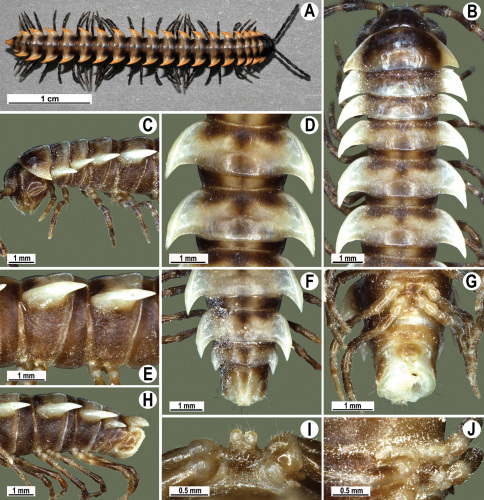
*Orthomorpha pterygota* Golovatch, 1998, ♂ from Kathu Waterfall (**A**), ♂ from Panwa Cape (**B-J**). **A** habitus, live coloration **B, C** anterior part of body, dorsal and lateral views, respectively **D, E** segments 10 and 11, dorsal and lateral views, respectively **F, G, H** posterior part of body, dorsal, ventral and lateral views, respectively **I, J** sternal cones between coxae 4, subcaudal and sublateral views, respectively.

Coloration of live animals (Fig. 65A) dark brown with contrasting orange paraterga and orange-brown hind parts of metaterga; coloration of alcohol material upon long-term preservation faded to basically light brown (holotype) to dark castaneous brown with contrasting pallid to light greyish paraterga, most of mid-dorsal parts of metaterga and prozona, as well as epiproct (Figs 64 & 65B-H).

**Figure 66. F66:**
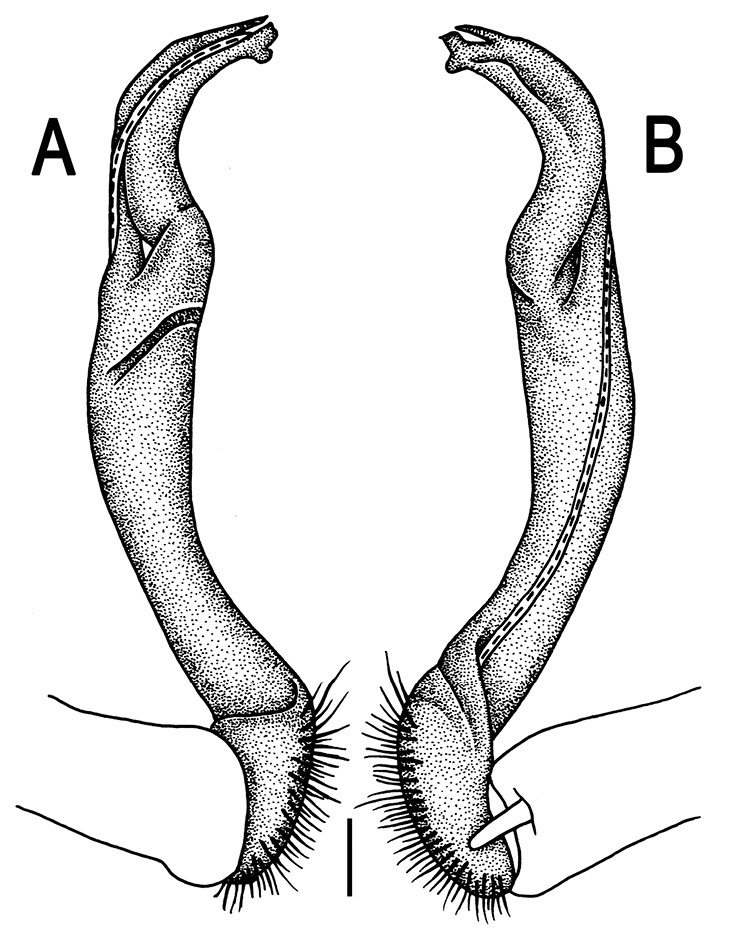
*Orthomorpha pterygota* Golovatch, 1998, ♂ holotype. **A, B** right gonopod, lateral and mesal views, respectively.Scale bar: 0.2 mm.

**Figure 67. F67:**
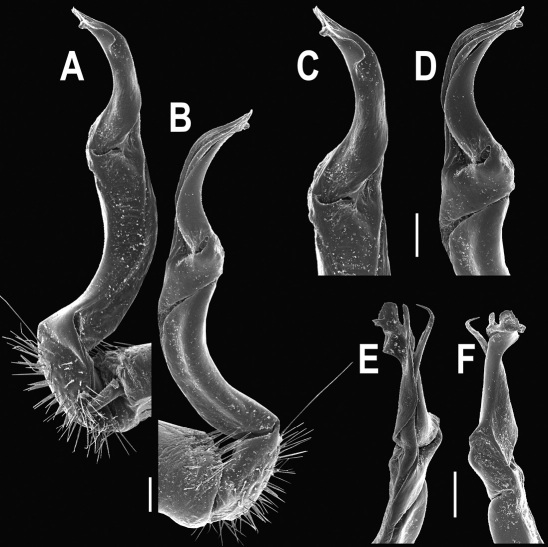
*Orthomorpha pterygota* Golovatch, 1998, ♂ from Panwa Cape. **A, B** right gonopod, mesal and lateral views, respectively **C–F** distal part of right gonopod, mesal, lateral, subcaudal and suboral views, respectively. Scale bar: 0.2 mm.

**Figure 68.**
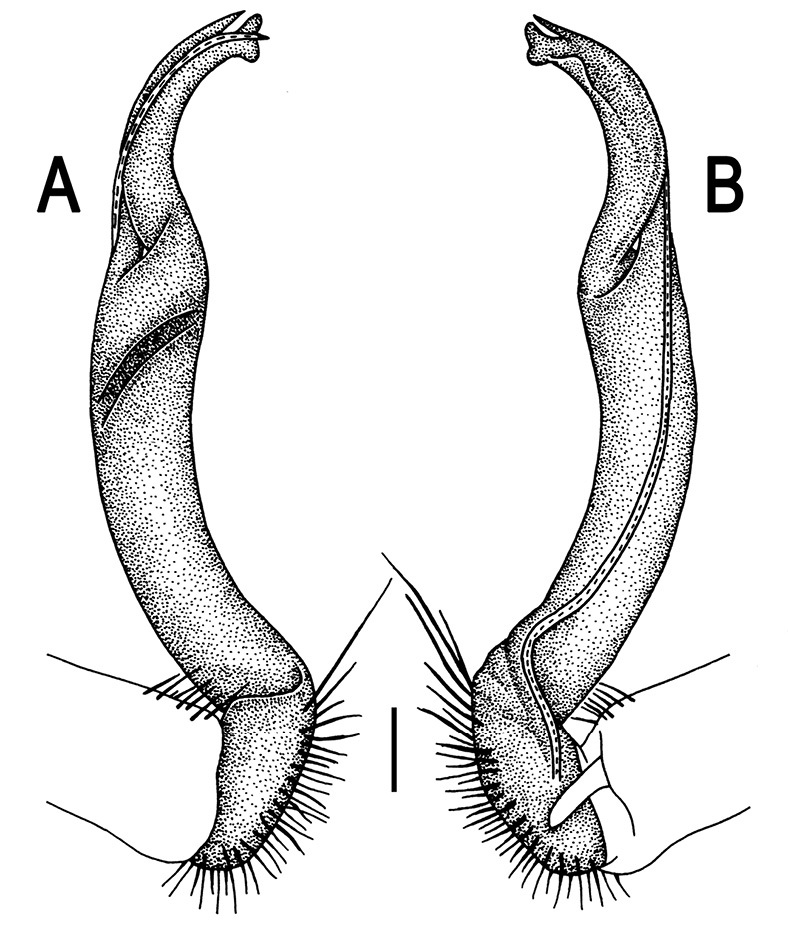
*Orthomorpha pterygota* Golovatch, 1998, ♂ from Panwa Cape. **A, B** right gonopod, lateral and mesal views, respectively. Scale bar: 0.2 mm.

#### Remarks.

The original description of this species was so complete and detailed (Golovatch 1998) that no redescription seems to be necessary. Instead, we only provide new illustrations (Figs 64–68) showing the coloration, structural details and gonopod conformation of *Orthomorpha pterygota*, based both on the holotype and new samples.

This species occurs, so far as known, only on Phuket Island and in the adjacent Krabi Province, southern Thailand.

### Orthomorpha horologiformis

Golovatch, 1998

http://species-id.net/wiki/Orthomorpha_horologiformis

**Figure 69.**
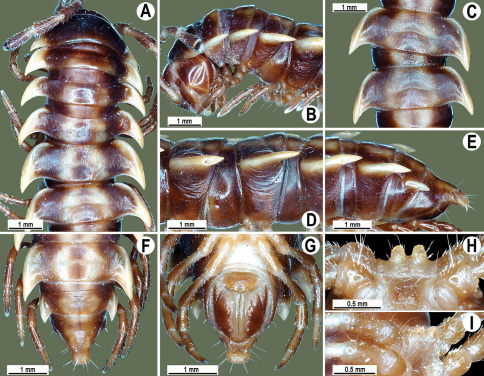
*Orthomorpha holorogiformis* Golovatch, 1998, ♂ holotype. **A, B** anterior part of body, dorsal and lateral views, respectively **C, D** segments 10 and 11, dorsal and lateral views, respectively **E–G** posterior part of body, lateral, dorsal and ventral views, respectively **H, I** sternal cones between coxae 4, subcaudal and sublateral views, respectively.

Figs 69 [Fig F70] [Fig F71] [Fig F72] 73

Orthomorpha horologiformis Golovatch 1998: 39 (D).
Orthomorpha horologiformis – Enghoff 2005: 97 (M, R); Decker 2010: 31 (R).

#### Holotype.

♂(ZMUC), Thailand, Trang Prov., Hat Chao Mai National Park, 7°19'N, 99°27'E, gallery tropical forest near coast, < 20 m, 26.10.1991, leg. M. Andersen, O. Martin & N. Scharff.

#### Other material examined.

1 ♂ (CUMZ), Thailand, Nakhon Si Thammarat Prov., Nopphitam Distr., Krungshing Waterfall, 9°12'43"N, 100°07'29"E, 27.10.2009, leg. R. Chanabun.1 ♀ (CUMZ), Phang Nga Prov., Mueang Phang Nga Distr., near Phung Chang Cave, 8°26'34"N, 98°30'59"E, 10.12.2009, leg. C. Sutcharit. 2 ♂, 2 ♀ (ZMUC), 2 ♂, 2 ♀ (ZMUM), 6 ♂, 4 ♀, 4 juv. (CUMZ), Krabi Prov., Khlong Thom Distr., Samorakot, 7°55'22"N, 99°15'35"E, 15.01.2009, leg. C. Sutcharit, N. Likhitrakarn & R. Chanabun. 1 ♀, 1 juv. (CUMZ), same Prov., Mueang Krabi Distr., Bantupprik School, ca 40 m, 8°10'50"N, 98°52'49"E, 16.01.2009, leg. R. Chanabun. 1 ♂ (CUMZ), Trang Prov., Mueang Trang Distr., 03.08.2010, leg. N. Likhitrakarn. 2 ♂, 4 ♀, 1 juv. (CUMZ), same Prov., Na Yong Distr., Khao Chao Priest Residence, 7°33'57"N, 99°46'27"E, 14.01.2009, leg. C. Sutcharit, N. Likhitrakarn & R. Chanabun. 2 ♂, 1 ♀ (CUMZ), Phatthalung Prov., Srinagarindra Distr., Sumano Cave, 7°59'08"N, 100°27'26"E, 15.10.2006, leg. C. Sutcharit. 3 ♂, 4 ♀ (CUMZ), same Prov., Khao Chaison Distr., Tham Nam Yen Hot Spring, ca 20 m, 7°27'02"N, 100°07'52"E, 01.01.2009, leg. C. Sutcharit, N. Likhitrakarn & R. Chanabun. 1 ♂ (CUMZ), Satun Prov., Mueang Satun Distr., Tarutao National Park, Ao Talo Wow, 7°02'30"N, 100°08'20"E, 8.04.2008, leg. R. Chanabun. 1 ♂ (CUMZ), same Distr., Tarutao National Park, Ao Chak Wow, 6°42'36"N, 99°38'29"E, 0.04.2008, leg. P. Pimvichai.

#### Descriptive notes.

Length 27–36 (♂) or 30–38 mm (♀), width of midbody pro- and metazona 2.1–3.5 and 3.6–4.5 mm (♂), 2.7–3.5 and 4.0–5.1 mm (♀), respectively.

**Figure 70. F70:**
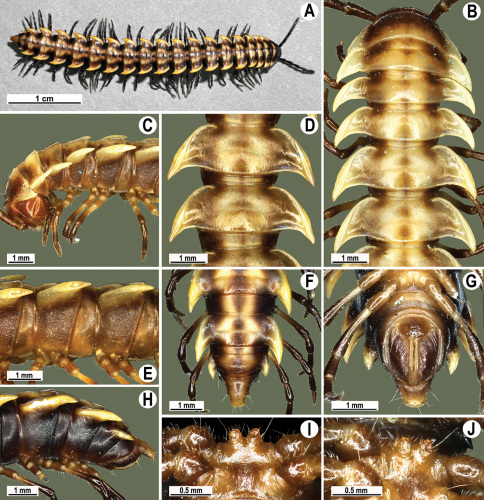
*Orthomorpha holorogiformis* Golovatch, 1998, ♂ from Phung Chang Cave (**A**), ♂ from Khao Chao Priest Residence (**B–J**). **A** habitus, live coloration **B, C** anterior part of body, dorsal and lateral views, respectively **D, E** segments 10 and 11, dorsal and lateral views, respectively **F, G, H** posterior part of body, dorsal, ventral and lateral views, respectively **I, J** sternal cones between coxae 4, subcaudal and sublateral views, respectively.

Coloration of live animals (Fig. 70A) brown to dark castaneous with light yellow-brown paraterga and most of central parts of metaterga and prozona; coloration of alcohol material upon long-term preservation faded to brown (holotype) with contrasting pallid to light yellow paraterga, middle parts of metaterga and prozona, and venter (Figs 69 & 70B-H).

**Figure 71. F71:**
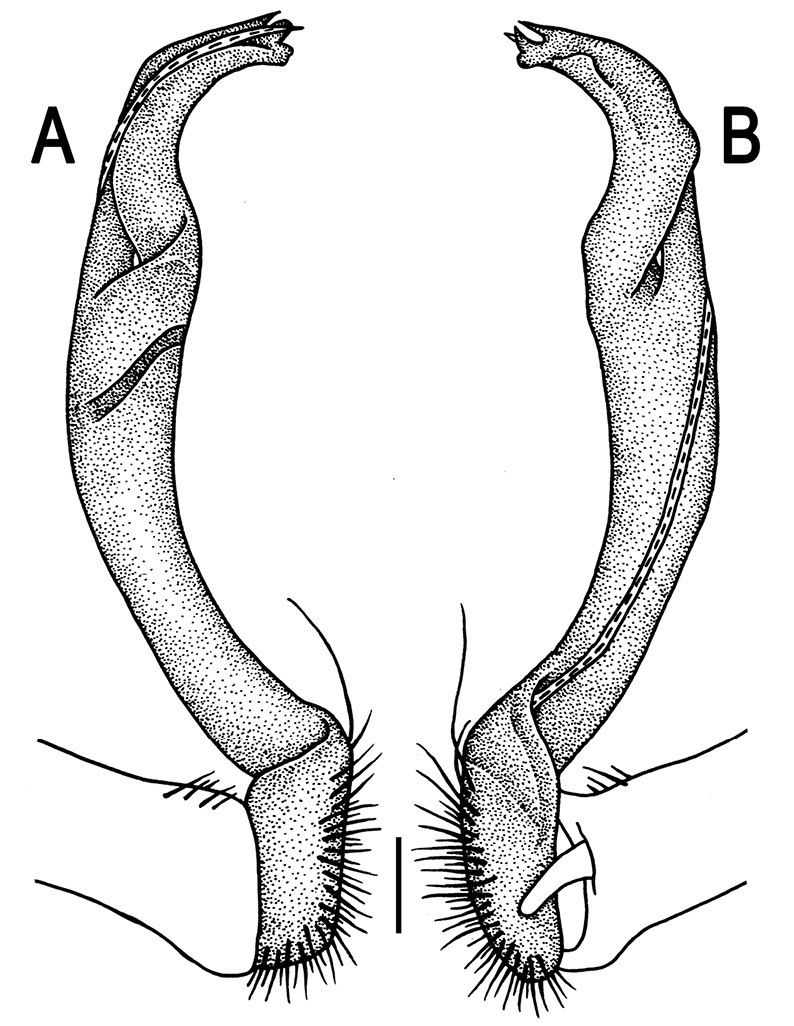
*Orthomorpha holorogiformis* Golovatch, 1998, ♂ holotype. **A, B** right gonopod, lateral and mesal views, respectively. Scale bar: 0.2 mm.

**Figure 72. F72:**
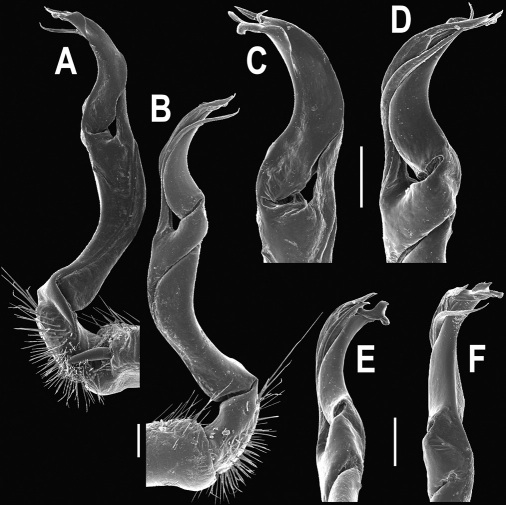
*Orthomorpha holorogiformis* Golovatch, 1998, ♂ from Samorakot. **A, B** right gonopod, mesal and lateral views, respectively **C-F** distal part of right gonopod, mesal, lateral, subcaudal and suboral views, respectively. Scale bar: 0.2 mm.

**Figure 73.**
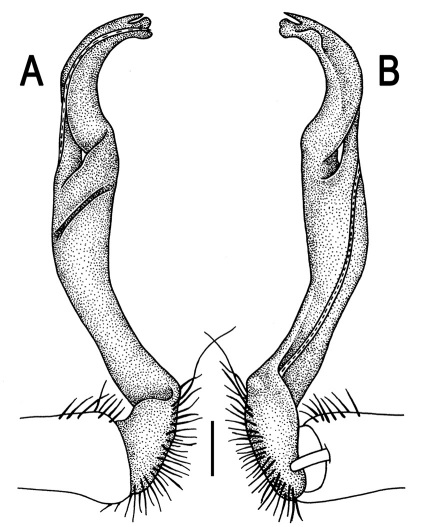
*Orthomorpha holorogiformis* Golovatch, 1998, ♂ from Khao Chao Priest Residence. **A, B** right gonopod, lateral and mesal views, respectively. Scale bar: 0.2 mm.

#### Remarks.

The original description of this species was so complete and detailed (Golovatch 1998) that no redescription seems to be necessary. Instead, we only provide new illustrations (Figs 69–73) showing the coloration, structural details and gonopod conformation of *Orthomorpha horologiformis*, based both on the holotype and new samples.

**Map 3.**
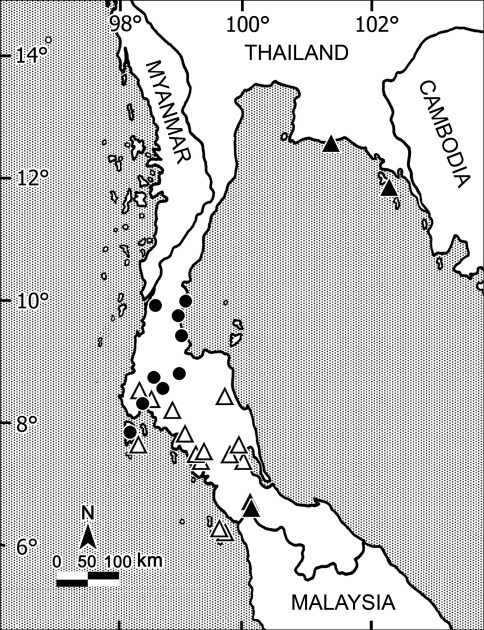
Distributions of three widespread species of *Orthomorpha*. Open triangle: *Orthomorpha holorogiformis* Golovatch, 1998; Filled circle: *Orthomorpha subkarschi* Golovatch, 1998; Filled triangle: *Orthomorpha thalebanica* Golovatch, 1998.

This species appears to be quite widespread in the southern parts of Thailand (Map 3).

### Orthomorpha subkarschi

Golovatch, 1998

http://species-id.net/wiki/Orthomorpha_subkarschi

**Figure 74.**
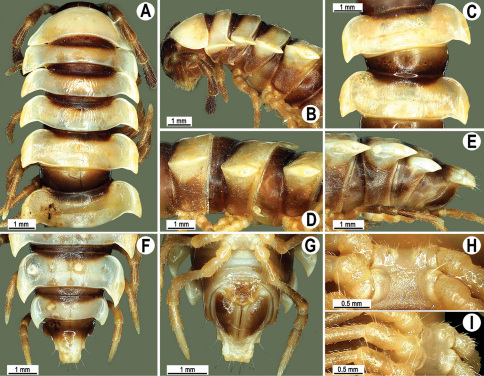
*Orthomorpha subkarschi* Golovatch, 1998, ♂ holotype. **A, B** anterior part of body, dorsal and lateral views, respectively **C, D** segments 8 and 9, dorsal and lateral views, respectively **E-F** posterior part of body, lateral, dorsal and ventral views, respectively **H, I** sternal cones between coxae 4, subcaudal and sublateral views, respectively.

Figs 74 [Fig F75] [Fig F76] [Fig F77] 78

Orthomorpha subkarschi Golovatch 1998: 39 (D).
Orthomorpha subkarschi – Enghoff 2005: 97 (M, R); Decker 2010: 31 (R).

#### Holotype.

♂ (ZMUC), Thailand, Phuket Prov., Mueang Phuket Distr., Ton Sai Waterfall, 8°01'N, 98°25'E, forest, < 200 m, 12.10.1991, leg. M. Andersen, O. Martin & N. Scharff.

#### Other material examined.

2 ♂, 1 ♀ (CUMZ), Thailand, Chumphon Prov., Lang Suan Distr., Khao Kriab Temple, ca 100 m, 9°49'03"N, 99°02'17"E, 05.06.2009, leg. R. Chanabun & N. Likhitrakarn. 4 ♂, 1 ♀, 1 juv. (CUMZ), Ranong Prov., Mueang Ranong Distr., Ngao Waterfall, 9°85'03"N, 98°62'78"E, 24.09.2006, leg. R. Chanabun. 1 ♀ (CUMZ), Surat Thani Prov., Tha Chana Distr., Tam Yai Temple, ca 10 m, 9°32'52"N, 99°10'49"E, 13.12.2009, leg. N. Likhitrakarn. 2 ♂, 1 ♀ (ZMUC), 2 ♂, 1 ♀ (ZMUM), 19 ♂, 8 ♀, (CUMZ), same Distr., Khiri Rom Temple, ca 10 m, 9°01'42"N, 98°59'05"E, 12.12.2009, leg. P. Pimvichai & N. Likhitrakarn. 1 ♀ (CUMZ), same Prov., Phanom Distr., Khao Phanom National Park, 26.10.2007, leg. C. Sutcharit. 1 ♂ (CUMZ), same Distr., at Padang Cave, 26.09.2009, leg. C. Sutcharit. 1 ♂(CUMZ), Phang Nga Prov., Thap Put Distr., Tham Kop, ca 70 m, 8°31'55 ˝ N, 98°34'37"E, 16.01.2009, leg. C. Sutcharit. 1 ♂ (CUMZ), same Distr., Tao Tong Waterfall, ca 20 m, 8°29'01 ˝ N, 98°35'05"E, 16.01.2009, leg. C. Sutcharit.

#### Descriptive notes.

Length 33–42 (♂) or 33–51 mm (♀), width of midbody pro- and metazona 2.6–3.6 and 4.0–6.0 mm (♂), 2.9–4.5 and 4.7–6.7 mm (♀), respectively.

**Figure 75. F75:**
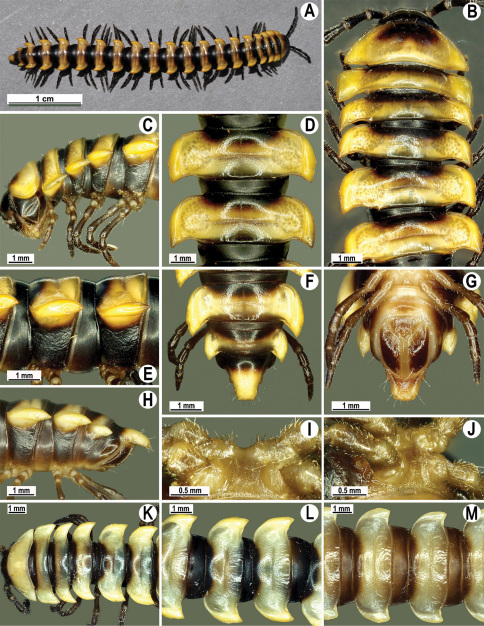
*Orthomorpha subkarschi* Golovatch, 1998, ♂ from Khiri Rom Temple (**A–J**), ♂ from Padang Cave (**K, L**), ♂ from Tham Yai Temple (**M**). **A** habitus, live coloration **B, C, K** anterior part of body, dorsal, lateral and dorsal views, respectively **D, E, L, M** segments 10 and 11, dorsal lateral, dorsal and dorsal views, respectively **F, G, H** posterior part of body, dorsal, ventral and lateral views, respectively **I, J** sternal cones between coxae 4, subcaudal and sublateral views, respectively.

Coloration of live animals (Fig. 75A) blackish-brown with contrasting yellow paraterga and epiproct, most of metaterga being light grey-brown; coloration of alcohol material upon long-term preservation blackish-brown to faded dark castaneous brown with contrasting yellow to pallid paraterga, epiproct and hind halves of metaterga (Figs 74, 75B-H & K-M); pattern mostly light creamy-yellowish, including paraterga’s dorsal and ventral parts, with contrasting dark brown to blackish cingulations of prozona and of anterior parts of metaterga. Collum either completely light or also bearing a dark, oblong, transverse, more or less large spot in front 1/3.

Calluses of several midbody and/or posterior metaterga (16–19) can be less strongly convex laterally (Fig. 75L & M).

**Figure 76. F76:**
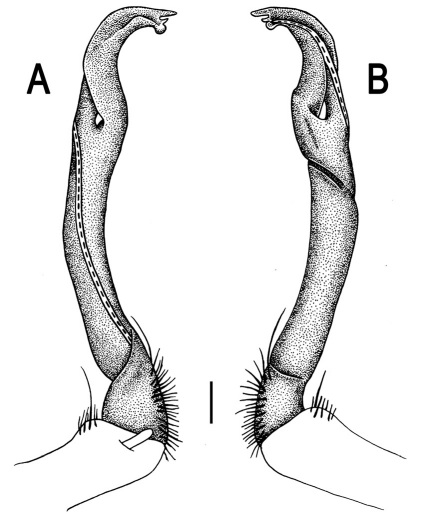
*Orthomorpha subkarschi* Golovatch, 1998, ♂ holotype. **A, B** left gonopod, mesal and lateral views, respectively.Scale bar: 0.2 mm.

**Figure 77. F77:**
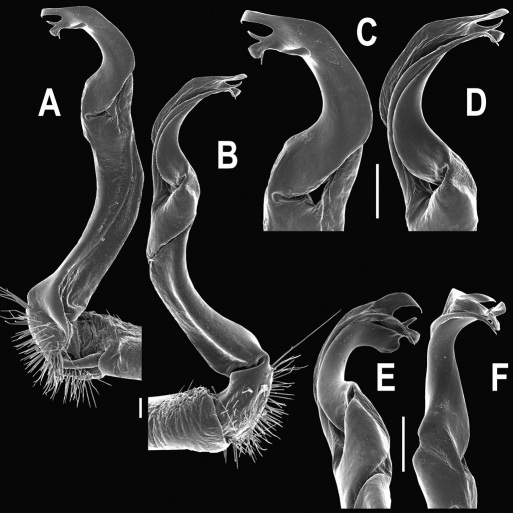
*Orthomorpha subkarschi* Golovatch, 1998, ♂ from Khiri Rom Temple. **A, B** right gonopod, mesal and lateral views, respectively **C-F** distal part of right gonopod, mesal, lateral, subcaudal and suboral views, respectively. Scale bar: 0.2 mm.

**Figure 78.**
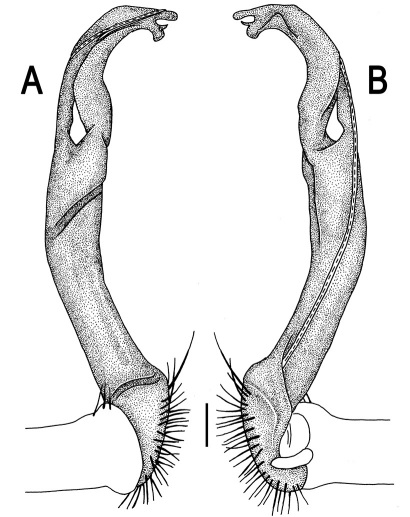
*Orthomorpha subkarschi* Golovatch, 1998, ♂ from Khiri Rom Temple. **A, B** right gonopod, lateral and mesal views, respectively. Scale bar: 0.2 mm.

Tip of solenophore narrowly rounded to sharply dentiform, rather depending on aspect (Figs 76–78).

#### Remarks.

The original description of this species was complete and detailed enough (Golovatch 1998) to require no complete redescription. Instead, we provide new illustrations (Figs 74–78) that show the coloration, structural details and gonopod conformation of *Orthomorpha subkarschi*, based both on the holotype and new samples. *Orthomorpha subkarschi* appears to be quite widespread in southern Thailand (Map 3).

### Orthomorpha banglangensis

Golovatch, 1998

http://species-id.net/wiki/Orthomorpha_banglangensis

Figs 79 80

Orthomorpha banglangensis Golovatch 1998: 38 (D).
Orthomorpha banglangensis – Enghoff 2005: 97 (M, R).

#### Holotype.

♂(ZMUC), Thailand, Yala Prov., Ban Lang National Park, 6°04'N, 101°11'E, lowland tropical rainforest, < 400 m, 20.10.1991, leg. M. Andersen, O. Martin & N. Scharff.

#### Descriptive notes.

**Figure 79.**
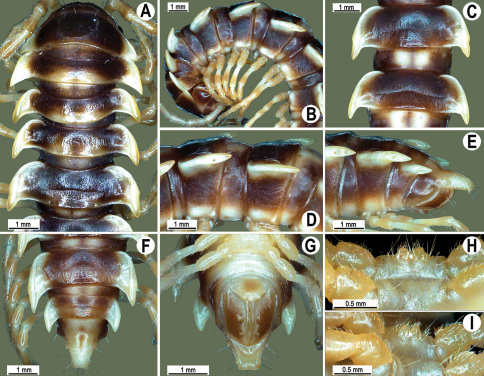
*Orthomorpha banglangensis* Golovatch, 1998, ♂ holotype. **A, B** anterior part of body, dorsal and lateral views, respectively **C, D** segments 10 and 11, dorsal and lateral views, respectively **E–G** posterior part of body, lateral, dorsal and ventral views, respectively **H, I** sternal cones between coxae 4, subcaudal and sublateral views, respectively.

Coloration of alcohol material upon long-term preservation probably faded to dark castanceous brown with contrasting pallid paraterga, middle part of prozona, epiproct, venter and pleurosternal carinae (Fig. 79).

**Figure 80.**
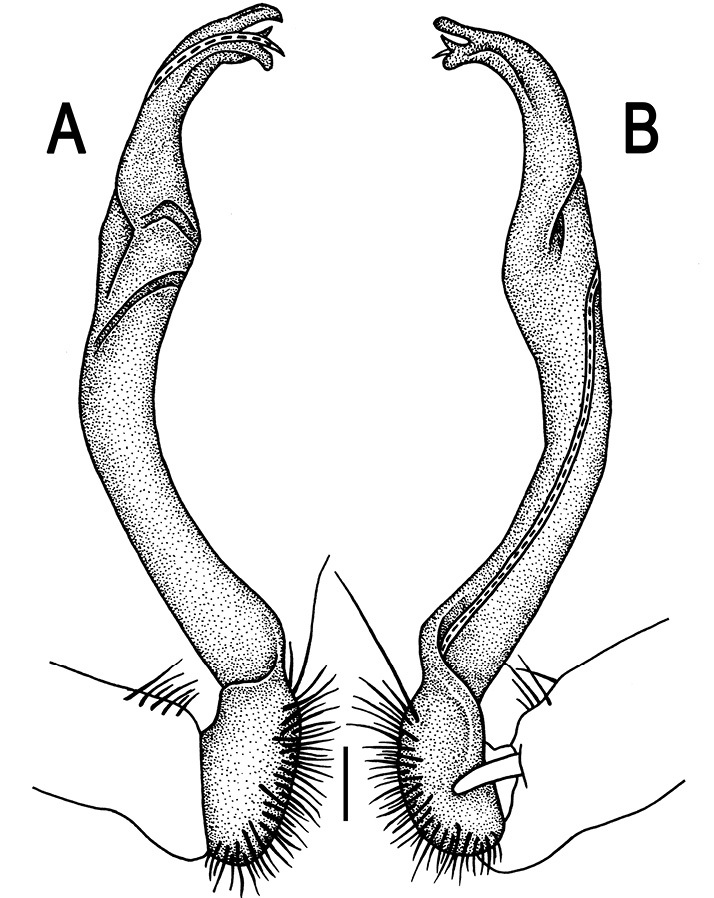
*Orthomorpha banglangensis* Golovatch, 1998, ♂ holotype. **A, B** right gonopod, lateral and mesal views, respectively.Scale bar: 0.2 mm.

#### Remarks.

The original description of this species was so complete and detailed (Golovatch 1998) that no redescription seems to be necessary. Instead, we only provide new illustrations (Figs 79 & 80) showing the coloration, structural details and gonopod conformation of *Orthomorpha banglangensis*, based on the holotype.

### Orthomorpha thalebanica

Golovatch, 1998

http://species-id.net/wiki/Orthomorpha_thalebanica

**Figure 81.**
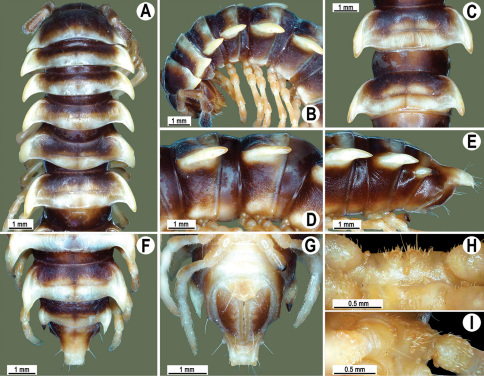
*Orthomorpha thalebanica* Golovatch, 1998, ♂ holotype. **A, B** anterior part of body, dorsal and lateral views, respectively **C, D** segments 10 and 11, dorsal and lateral views, respectively **E–G** posterior part of body, lateral, dorsal and ventral views, respectively **H, I** sternal cones between coxae 4, subcaudal and sublateral views, respectively.

Figs 81 [Fig F82] [Fig F83] [Fig F84] 85

Orthomorpha thalebanica Golovatch 1998: 37 (D).
Orthomorpha thalebanica – Enghoff 2005: 98 (M, R); Decker 2010: 32 (R).

#### Holotype.

♂(ZMUC), Thailand, Satun Prov., Thale Ban National Park, 6°42'N, 100°10'E, lowland tropical, < 400 m, 20.10.1991, leg. M. Andersen, O. Martin & N. Scharff.

#### Other material examined.

1 ♀ (CUMZ), Thailand, Satun Prov., Khuan Don Distr., Thale Ban National Park, ca 110 m, 6°42'41"N, 100°14'53"E, 17.05.2010, leg. N. Likhitrakarn.1 ♂ (ZMUC), 1 ♂ (ZMUM), 2 ♂ (CUMZ), same locality, 04.08.2010, leg. P. Poolprasert. 1 ♀ (CUMZ), same Distr., at Nara Cave, ca 40 m, 6°47'30"N, 100°05'09"E, forest, 13.01.2009, leg. N. Likhitrakarn.

#### Descriptive notes.

Length 36–42 (♂) or 39–46 mm (♀), width of midbody pro- and metazona 2.8–3.6 and 4.9–5.6 mm (♂), 3.6–4.1 and 5.3–6.0 mm (♀), respectively.

**Figure 82. F82:**
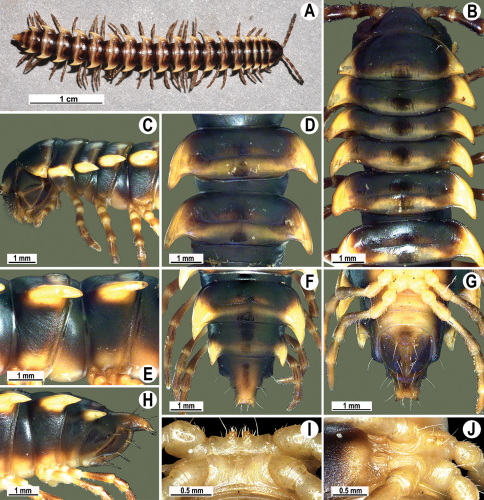
*Orthomorpha thalebanica* Golovatch, 1998, ♂. **A** habitus, live coloration **B, C** anterior part of body, dorsal and lateral views, respectively **D, E** segments 10 and 11, dorsal and lateral views, respectively **F, G, H** posterior part of body, dorsal, ventral and lateral views, respectively **I, J** sternal cones between coxae 4, subcaudal and sublateral views, respectively.

Coloration of live animals (Fig. 82A) nearly same as in specimens kept in alcohol for a long time, except for paraterga being slightly more infuscate in live individuals, in all cases dark castaneous brown with contrasting light yellow-brown to pallid paraterga, tip of epiproct, venter and pleurosternal carinae; rear 1/2–1/4 of metaterga and mid-dorsal regions of prozona slightly infuscate, yellow-brown to nearly pallid (Figs 81 & 82).

**Figure 83. F83:**
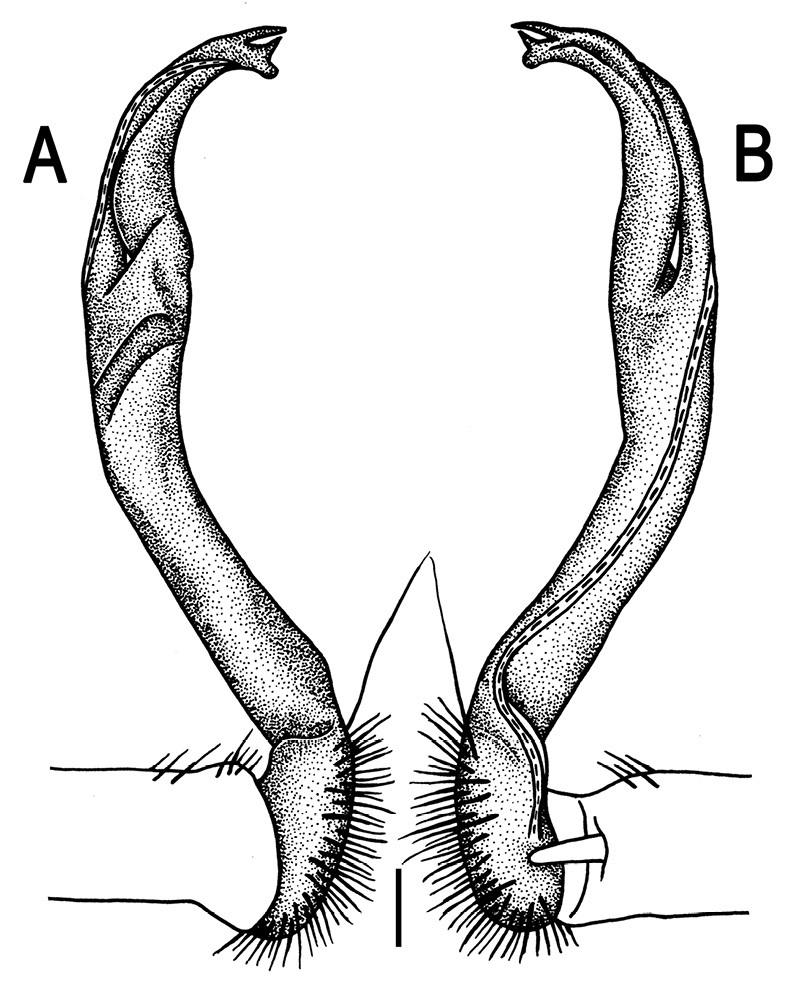
*Orthomorpha thalebanica* Golovatch, 1998, ♂ holotype. **A, B** right gonopod, lateral and mesal views, respectively.Scale bar: 0.2 mm.

**Figure 84. F84:**
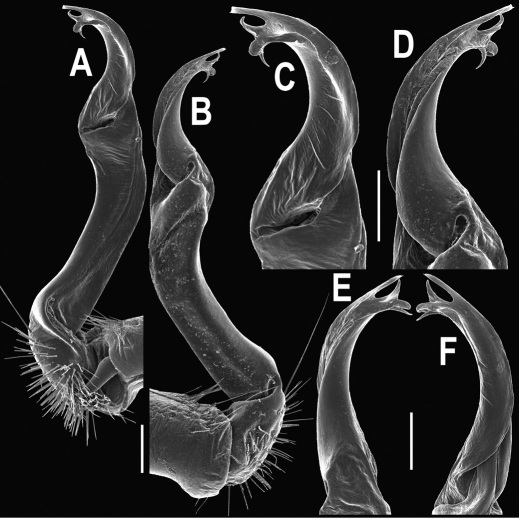
*Orthomorpha thalebanica* Golovatch, 1998, ♂. **A, B** right gonopod, mesal and lateral views, respectively **C-F** distal part of right gonopod, mesal, lateral, subcaudal and suboral views, respectively. Scale bar: 0.2 mm.

**Figure 85.**
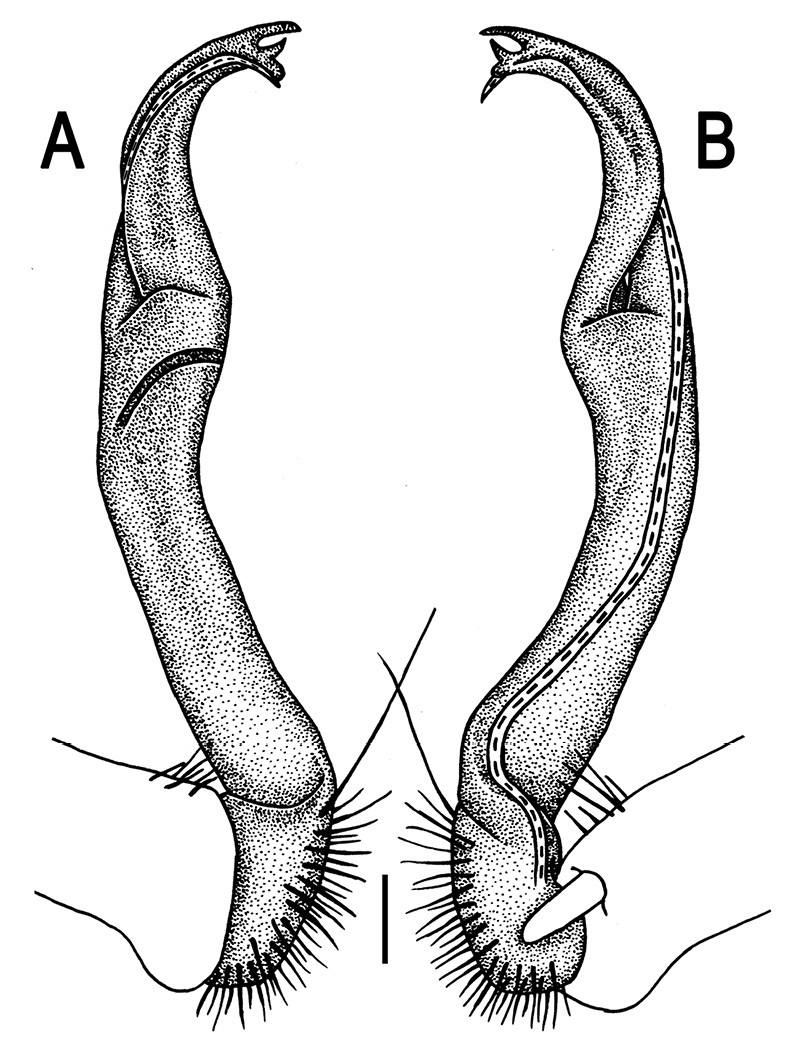
*Orthomorpha thalebanica* Golovatch, 1998, ♂. **A, B** right gonopod, lateral and mesal views, respectively**.** Scale bar: 0.2 mm.

#### Remarks.

The original description of this species was so complete and detailed (Golovatch 1998) that no redescription seems to be necessary. Instead, we only provide new illustrations (Figs 81–85) showing the coloration, structural details and gonopod conformation of *Orthomorpha thalebanica*, based both on the holotype and new samples. This species appears to be rather widespread in southern Thailand (Map 3).

### Orthomorpha lauta

Golovatch, 1998

http://species-id.net/wiki/Orthomorpha_lauta

**Figure 86.**
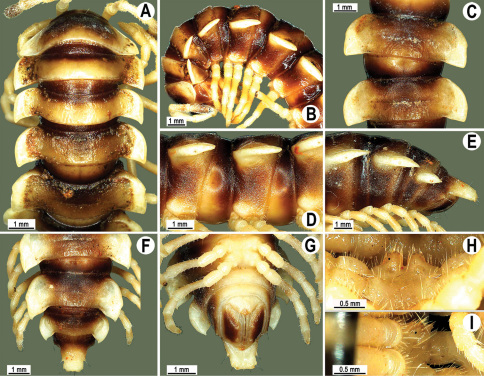
*Orthomorpha lauta* Golovatch, 1998, ♂ holotype. **A, B** anterior part of body, dorsal and lateral views, respectively **C, D** segments 10 and 11, dorsal and lateral views, respectively **E–G** posterior part of body, lateral, dorsal and ventral views, respectively **H, I** sternal cones between coxae 4, subcaudal and sublateral views, respectively.

Figs 86 [Fig F87] [Fig F88] [Fig F89] 90

Orthomorpha lauta Golovatch 1998: 37 (D).
Orthomorpha lauta – Enghoff 2005: 97 (M, R).

#### Holotype.

♂(ZMUC), Thailand, Krabi Prov., road between Krabi and Phuket, 10 km north of Krabi, 8°09'N, 98°50'E, lowland tropical rainforest, < 200 m, 13.10.1991, leg. M. Andersen, O. Martin & N. Scharff.

#### Other material examined.

2 ♂, 2 ♀ (ZMUC), 2 ♂, 2 ♀ (ZMUM), 15 ♂, 16 ♀ (CUMZ), Thailand, Surat Thani Prov., Kanchanadit Distr., Tham Khao Phanomwang, 9°09'24"N, 100°0'25"E, 10.10.2008, leg. S. Panha, N. Likhitrakarn & R. Chanabun. 1 ♂ (CUMZ), Surat Thani Prov., Ko Samui Distr., Na Mueang Waterfall, 27.01.2006, leg. C. Sutcharit. 2 ♂, 2 ♀ (CUMZ), same locality, 06.06.2009, leg. N. Likhitrakarn. 1 ♂ (CUMZ), same Distr., Hinlard Waterfall, 27.10.2006, leg. C. Sutcharit. 1 ♂ (CUMZ), Krabi Prov., Ao Luek Distr., Tham Sra Yuan Thong, 8°36'31"N, 99°14'58"E, 09.10.2006, leg. C. Sutcharit. 2 ♂, 2 ♀ (CUMZ), same Prov., Mueang Krabi Distr., Tham Suae Temple, 8°10'69"N, 98°92'22"E, 07.10.2006, leg. C. Sutcharit. 2 ♂, 4 ♀, 3 juv. (CUMZ), same locality, 15.01.2009, leg. N. Likhitrakarn. 1 ♂, 2 ♀ (CUMZ), same Distr., Forest Reseach Station, 8°08'28"N, 98°51'11"E, 16.01.2009, leg. R. Chanabun. 3 ♂, 2 ♀ (CUMZ), same Prov., Khlong Thom Distr., Samorakot, 7°55'22 ˝ N, 99°15'35"E, 15.01.2009, leg. N. Likhitrakarn. 1 ♂, 1 ♀(CUMZ), Trang Prov., Na Yong Distr., Khao Chang Botanical Garden, ca 50 m, 7°32'45"N, 99°46'27"E, 14.01.2009, leg. N. Likhitrakarn. 2 ♂ (CUMZ), same Distr., Tham Khao Chang Hai, ca 40 m, 7°35'25"N, 99°40'07"E, 13.01.2009, leg. N. Likhitrakarn. 1 ♂, 2 ♀ (CUMZ), same Distr., Chao Priests Residence, ca 60 m, 7°33'57"N, 99°46'27"E, 28.01.2006, leg. C. Sutcharit. 4 ♂, 3 ♀ (CUMZ), same locality, 14.01.2009, leg. C. Sutcharit & N. Likhitrakarn. 4 ♂ (CUMZ), Phatthalung Prov., Khuan Khaum Distr., Tham Wang Thong Temple, ca 10 m, 7°40'53"N, 100°0'56"E, 11.01.2009, leg. C. Sutcharit & N. Likhitrakarn. 1 ♂, 2 juv. (CUMZ), same Prov., Mueang Phatthalung Distr., Tham Malai Thep Nimit Priest Residence, ca 20 m, 7°38'08"N, 100°05'04"E, 11.01.2009, leg. C. Sutcharit & N. Likhitrakarn. 3 ♂, 3 ♀ (CUMZ), same Distr., Tham Kuhasawan, ca 20 m, 7°37'12"N, 100°04'51"E, 12.01.2009, leg. C. Sutcharit & N. Likhitrakarn. 1 ♂, 1 ♀ (CUMZ), Satun Prov., Khuan Don Distr., Thale Ban National Park, ca 60 m, 6°42'41"N, 100°10'09"E, 13.10.2009, leg. C. Sutcharit & N. Likhitrakarn. 3 ♂, 1 ♀, 1 juv. (CUMZ), Songkhla Prov., Rattaphum Distr., Tham Sri Kesorn, ca 50 m, 7°04'38"N, 100°09'56"E, 12.01.2009, leg. N. Likhitrakarn. 1 ♀ (CUMZ), same Prov., Hat Yai Distr., Tonngachang Waterfall, ca 110 m, 6°56'54"N, 100°14'08"E, 12.01.2009, leg. C. Sutcharit & N. Likhitrakarn. 2 ♂, (CUMZ), Malaysia, Perlis, Wang Kelian, 7°08'07"N, 100°19'24"E, 26.03.2009, leg. C. Sutcharit. 1 ♂ (CUMZ), Penang, Balik Pulau, 5°38'13"N, 100°21'38"E, 25.03.2009, leg. C. Sutcharit. 2 ♂, 1 ♀ (CUMZ), Kelantan, Bukit Landak, 5°26'N, 102°27'E, 11.12.2007, leg. C. Sutcharit.

#### Descriptive notes.

Length 35–49 (♂) or 35–52 mm (♀), width of midbody pro- and metazona 3.1–4.1 and 5.0–6.5 mm (♂), 3.4–4.8 and 5.0–7.1 mm (♀), respectively.

**Figure 87. F87:**
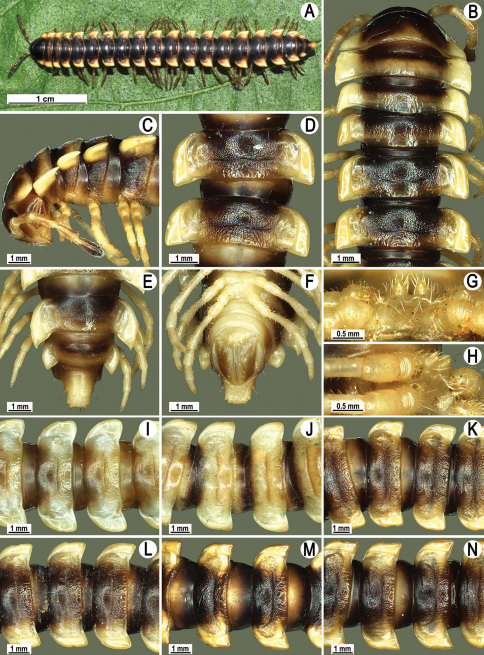
*Orthomorpha lauta* Golovatch, 1998, ♂ from Na Mueang Waterfall (**A, L**), ♂ from Tham Khao Panomwang (**B–H, K, M, N**), ♂ from Tham Sri Kesorn (**I**), ♂ from Bkit Pulau (**J**). **A** habitus, live coloration **B, C** anterior part of body, dorsal and lateral views, respectively **D, I-N** segments 10 and 11, dorsal views **E, F** posterior part of body, dorsal and ventral views, respectively **G, H** sternal cones between coxae 4, subcaudal and sublateral views, respectively.

Coloration of live animals (Fig. 87A) dark castaneous brown with contrasting light yellowish paraterga and tip of epiproct; coloration upon long-term preservation in alcohol sometimes slightly faded, ranging from light brown or medium red-brown with more or less contrasting pallid to yellowish-brown paratergal calluses and epiproct, up to blackish-brown with contrasting light brown to grey-yellow calluses and epiproct (Fig. 87A-F & I-N); front halves of prozona (especially dorsally), caudal edges of metaterga and legs, sometimes also venter, flavous, light yellow-brown to brownish (Figs 86 & 87).

Collum with caudal corner of paraterga ranging from obtusangular (described as being about 100˚ in holotype, Fig. 86A & B), via subrectangular (Fig. 87C), to evidently acutangular. Calluses of paraterga mostly broad and extending behind tergal margin, increasingly well so on segements 7(8)-19, but sometimes slightly narrower and not surpassing it, forming instead a subrectangular turn mesally (Figs 86A-G, 87A-F & I-N). Caudal corner of paraterga remaining from acute and pointed thoughout (♂ from Tham Khao Phanomwang Temple, Fig. 87N) to mostly narrowly rounded (♂ from Tham Sri Kesorn, Fig. 87I), with all intergradations in-between (Figs 86A, C & F, 87A, B, D, E & J-M).

Metatergal sulcus visible on segments 5–18, more rarely incomplete also on segment 19 (holotype). Pleurosternal carinae expressed as complete high crests with a sharp caudal tooth on segment 2, thereafter increasingly well divided into a front bulge and a caudal tooth, the latter sharp or rounded, on segments 2–7; both front bulge and caudal tooth increasingly strongly reduced either until segment 10 (11), with a small sharp tooth until segment 16 (17) (♂), or expressed as complete high crests with a sharp caudal tooth on segments 2–4, thereafter increasingly well divided into a front bulge and a caudal tooth, sharp or rounded, on segments 5–10, a small sharp tooth until segment 16 (17) (♀). Midbody legs ca 1.1–1.3 (♂) or 0.9–1.2 times (♀) as long as body height.

Base of lamina lateralis with a small, but conspicuous ventral knob (like in *Orthomorpha fluminoris*). Tip of gonopod with terminal lobule longest, bearing microdenticulations or a more or less evident fringe at external edge (Fig. 89C & D). Middle spiniform prong sometimes slightly smaller than usual (Fig. 90).

**Figure 88. F88:**
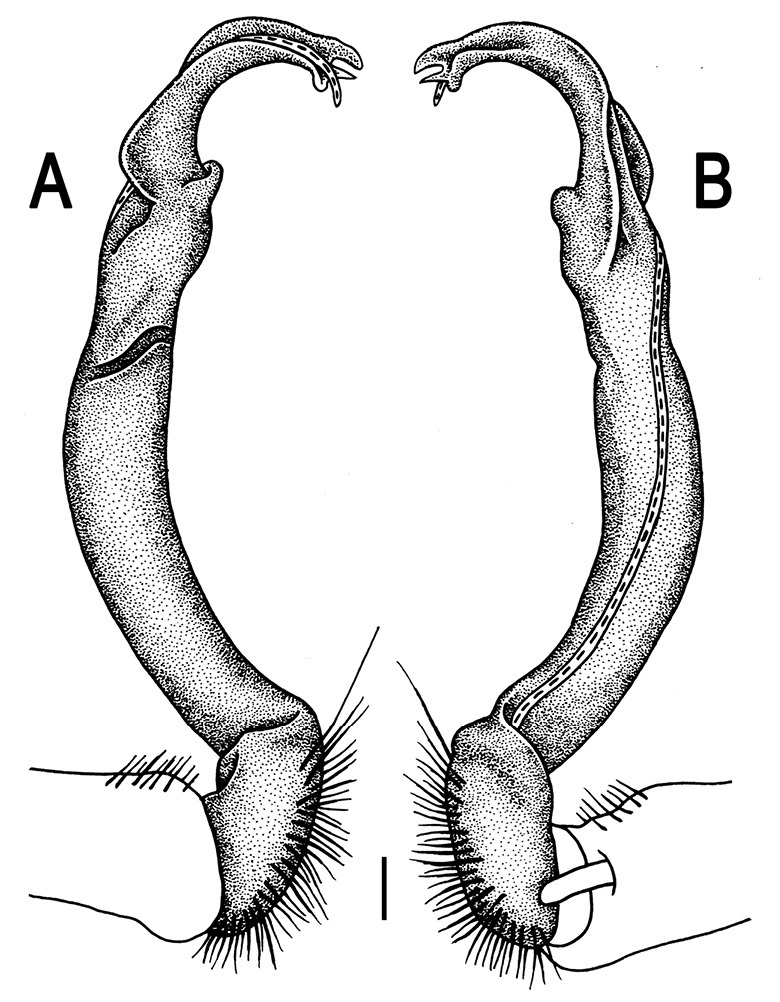
*Orthomorpha lauta* Golovatch, 1998, ♂ holotype. **A, B** right gonopod, lateral and mesal views, respectively.Scale bar: 0.2 mm.

**Figure 89. F89:**
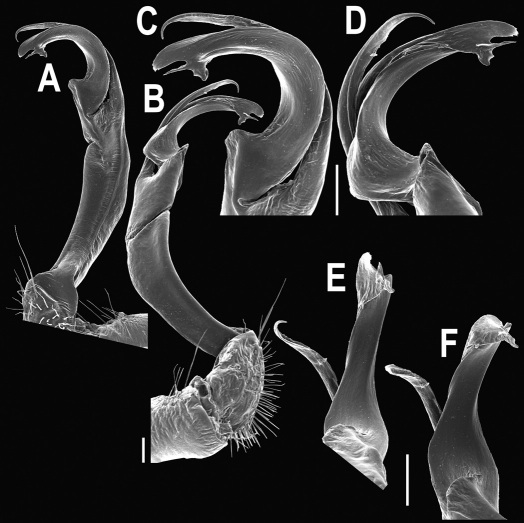
*Orthomorpha lauta* Golovatch, 1998, ♂ from Tham Khao Panomwang. **A, B** right gonopod, mesal and lateral views, respectively **C-F** distal part of right gonopod, mesal, lateral, suboral and suboral views, respectively. Scale bar: 0.2 mm.

**Figure 90.**
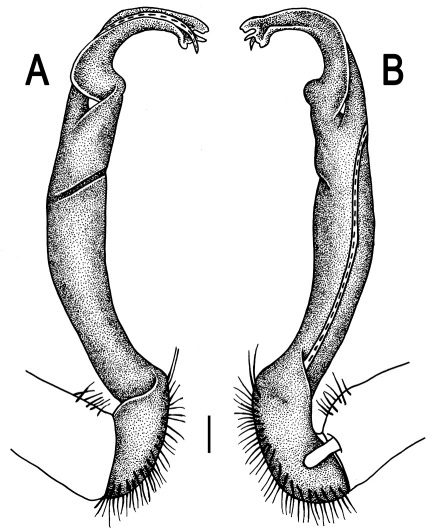
*Orthomorpha lauta* Golovatch, 1998, ♂ from Tham Khao Panomwang. **A, B** right gonopod, lateral and mesal views, respectively. Scale bar: 0.2 mm.

#### Remark.

**Map 4.**
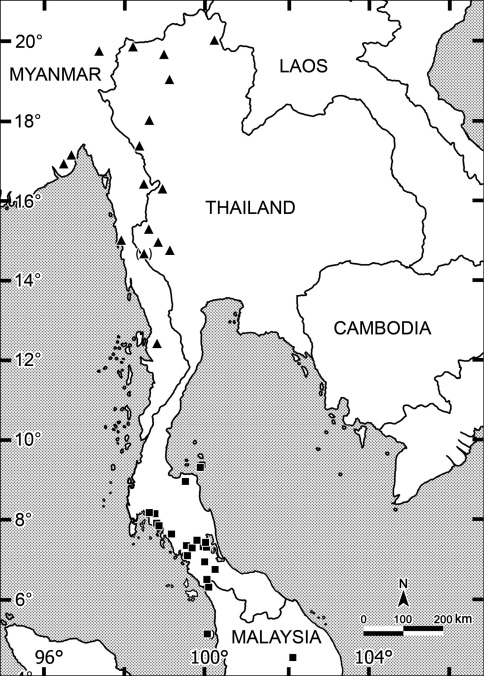
Distribution of two widespread species of *Orthomorpha*. Filled square: *Orthomorpha lauta* Golovatch, 1998; Filled triangle: *Orthomorpha insularis* Pocock, 1895.

This species appears to be widespread in southern Thailand (Map 4).

### Orthomorpha insularis

Pocock, 1895

http://species-id.net/wiki/Orthomorpha_insularis

**Figure 91.**
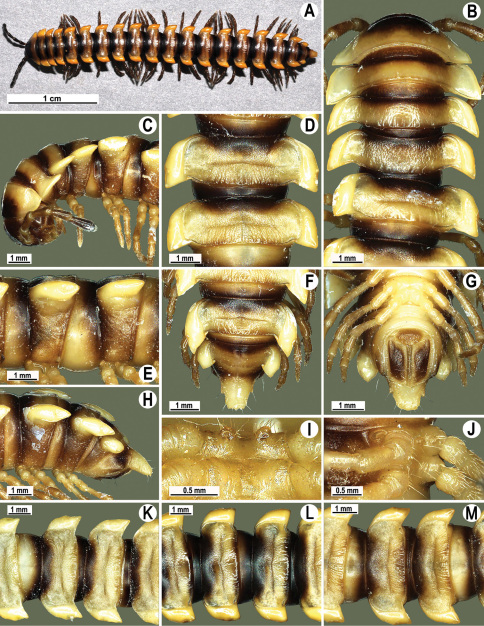
*Orthomorpha insularis* Pocock, 1895, ♂ from Doi Suthep National Park (**A–J, M**), ♂ from Phu Song National Park (**K**), ♂ from Palong Temple (**L**). **A** habitus, live coloration **B, C** anterior part of body, dorsal and lateral views, respectively **D, E, K-M** segments 10 and 11, dorsal, lateral, dorsal, dorsal and dorsal views, respectively **F, G, H** posterior part of body, dorsal, ventral and lateral views, respectively **I, J** sternal cones between coxae 4, subcaudal and sublateral views, respectively.

Figs 91 [Fig F92] [Fig F93] [Fig F94] [Fig F95] 96

Orthomorpha insularis Pocock 1895: 817 (D).
Orthomorpha clivicola Pocock 1895: 819 (D).
Orthomorpha palonensis Pocock 1895: 820 (D).
Orthomorpha monticola Pocock 1895: 820 (D).
Orthomorpha gestri Pocock 1895: 820 (D).
Orthomorpha gestri – Attems 1936: 205 (M, R); 1937: 94 (M); Jeekel 1963: 265 (M); 1964: 361 (M); 1968: 56 (M).
Orthomorpha Gestri – Attems 1898: 328 (D); 1914: 238 (M).
Orthomorpha monticola – Attems 1898: 328 (D); 1914: 238 (M); 1936: 205 (M, R); 1937: 94 (M); Jeekel 1963: 265 (M); 1964: 361 (M, D); 1968: 56 (M).
Orthomorpha palonensis – Attems 1898: 328 (D); 1914: 238 (M); 1936: 205 (M, R); 1937: 94 (M); Jeekel 1963: 265 (M); 1964: 361 (M); 1968: 56 (M).
Orthomorpha clivicola – Attems 1898: 328 (D); 1914: 238 (M); 1936: 204 (M, R); 1937: 93 (M); Jeekel 1963: 265 (M); 1964: 361 (M); 1968: 56 (M).
Orthomorpha insularis – Attems 1898: 328 (D); 1914: 238 (M); Jeekel 1963: 265 (M); 1964: 361 (M, D); 1968: 56 (M); 1970: 293 (D); Hoffman 1977: 700 (M); Golovatch 1998: 42 (D).
Orthomorpha karschi insularis Attems 1936: 199 (D).
Orthomorpha karschii insularis – Attems 1937: 71 (D).
Orthomorpha karschii intercedens Attems 1937: 71 (D).
Orthomorpha intercedens – Jeekel 1963: 265 (M); 1964: 361 (M); 1968: 56 (M); 1970: 293 (D); Enghoff 2005: 97 (M, R); Decker 2010: 31 (R).

#### Material examined.

2 ♀ (CUMZ), Thailand, Mae Hong Son Prov., Mueang Mae Hong Son Distr., Tham Woa Temple, 19°53'33"N, 98°08'40"E, 19.07.2008, leg. R. Chanabun. 2 ♂, 1 ♀ (CUMZ), Chiang Mai Prov., Mueang Chiang Mai Distr., Doi Suthep National Park, ca 1290 m, 18°48'09"N, 98°54'12"E, 23.04.2009, leg. N. Likhitrakarn. 2 ♂ (CUMZ), same locality, 28.11.2009, leg. N. Likhitrakarn. 1 ♀ (ZMUC), 1 ♀ (ZMUM), 2 ♂, 1 ♀, 2 juv. (CUMZ), same Prov., Chiang Dao Distr., Palong Temple, ca 470 m, 19°24'13"N, 98°55'16"E, 28.09.2010, leg. N. Likhitrakarn. 1 ♂ (CUMZ), Phayao Prov., Phu Sang Distr., Phu Sang National Park, 20°06'02"N, 100°37'56"E, 24.10.2008, leg. C. Sutcharit. 1 ♂, 1 ♀ (CUMZ), Tak Prov., Mae Sot Distr., Doi Mu Soe, 17°18'70"N, 99°33'50"E, 5.10.2008, leg. C. Sutcharit. 1 ♂, same Prov., Tha Song Yang Distr., at Mae Usu Cave, ca 140 m, 17°18'16"N, 98°09'21"E, 30.05.2009, leg. N. Likhitrakarn. 1 ♂ (CUMZ), same Prov., Umphang Distr., near Umphang City, ca 490 m 16°02'20"N, 98°52'0"E, 06.07.2009, leg. N. Likhitrakarn. 2 ♂, 1 ♀ (CUMZ), Kanchanaburi Prov., Thong Pha Phum Distr., Krienkravia Waterfall, ca 260 m, 14°58'55"N, 98°37'53"E, 10.07.2009, leg. C. Sutcharit. 2 ♀ (CUMZ), same locality, 08.05.2010, leg. N. Likhitrakarn. 1 ♀ (CUMZ), same locality, 19.12.2010, leg. N. Likhitrakarn. 1 ♂ (ZMUC), 1 ♂ (ZMUM), 3 ♂, 2 ♀ (CUMZ), same Distr., Thong Pha Phum, 15.08.2007, leg. S. Panha. 1 ♀, 11 juv. (CUMZ), same Prov., Sai Yok Distr., Daowadueng Cave National Park, ca 210 m, 14°28'12"N, 98°49'58"E, 11.07.2009, leg. C. Sutcharit.

#### Descriptive notes.

Length 33–41.5 mm (♂) to 34–51 mm (♀), width of midbody pro- and metazona 3.1–4.0 and 4.9–5.7 mm (♂), 3.2–4.5 and 5.2–7.0 mm (♀), respectively.

**Figure 92. F92:**
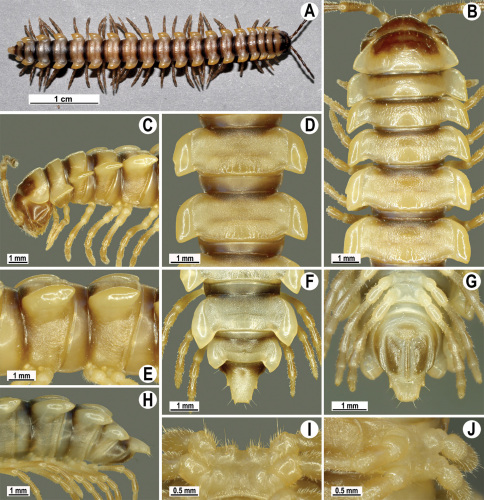
*Orthomorpha insularis* Pocock, 1895, ♂ from Krienkravia Waterfall. **A** habitus, live coloration **B, C** anterior part of body, dorsal and lateral views, respectively **D, E** segments 10 and 11, dorsal and lateral views, respectively **F, G, H** posterior part of body, dorsal, ventral and lateral views, respectively **I, J** sternal cones between coxae 4, subcaudal and sublateral views, respectively.

Coloration of live animals (Figs 91A & 92A) blackish brown with rather poorly contrasting light orange-brown to light brown paraterga and epiproct, rear halves to nearly entire metaterga sometimes a little more infuscate, brownish; coloration upon long-term preservation in alcohol basically same, but paraterga, epiproct, most of metatergal surface, as well as frontodorsal parts of prozona contrasting pallid, yellowish or light grey-brown (Figs 91B-H, K-M & 92B-H). A light centrocaudal spot to a broad caudal band on the collum seems to be characteristic of this species, despite its size variation from small and vague to large and clear in alcohol-preserved material (Figs 91B & C, 92B & C).

Antennae rather long, clavate (antennomere 6 broadest), extending behind body segment 3 (♂) or 2 (♀) dorsally.

Collum with caudal corner of paraterga ranging from obtusangular (Fig. 92A & B), via subrectangular (Fig. 91B & C), to evidently acutangular. Calluses of paraterga always very broad and their caudal corners mostly extending only to hind tergal margin, but sometimes, even within a single population, calluses slightly narrower, not reaching rear tergal margin, forming instead a subrectangular turn mesally (Figs 91B-E & 92B-E), extending increasingly beyond rear margin in hind body portion to only several caudal segments, with caudal tip remaining from acute and pointed thoughout (♂ from Phu Sang National Park, Fig. 91K) to mostly narrowly rounded (♂ from Paplong Temple, Fig. 91L & M). Metatergal sulcus visible on segments 5–18 (♂, ♀). Pleurosternal carinae expressed as complete high crests with a sharp caudal tooth on segments 2–4, thereafter increasingly well divided into a front bulge and a caudal tooth on segments 5–7, both increasingly strongly reduced in size until segment 15 or 16 (♂, ♀).

Midbody legs ca 1.1–1.4 (♂) to 0.9–1.3 times (♀) as long as body height.

**Figure 93. F93:**
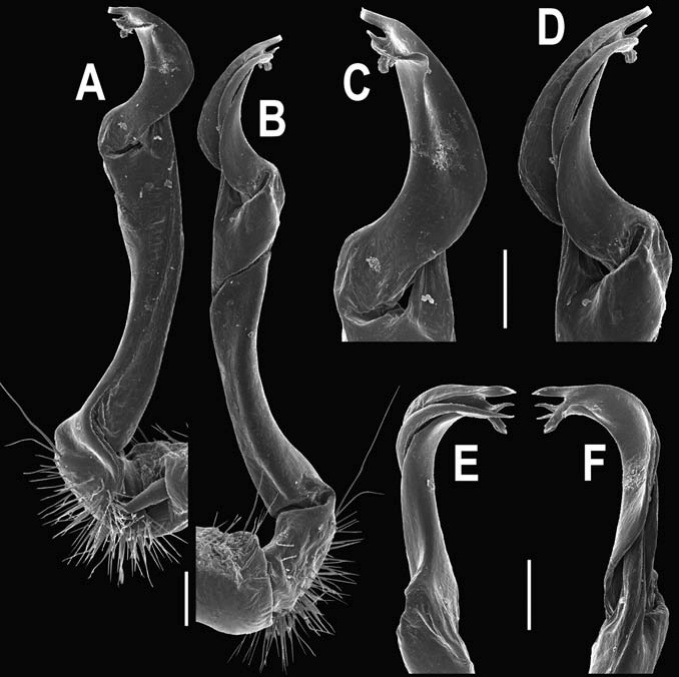
*Orthomorpha insularis* Pocock, 1895, ♂ from Doi Suthep National Park. **A, B** right gonopod, mesal and lateral views, respectively **C-F** distal part of right gonopod, mesal, lateral, suboral and subcaudal views, respectively. Scale bar: 0.2 mm.

**Figure 94. F94:**
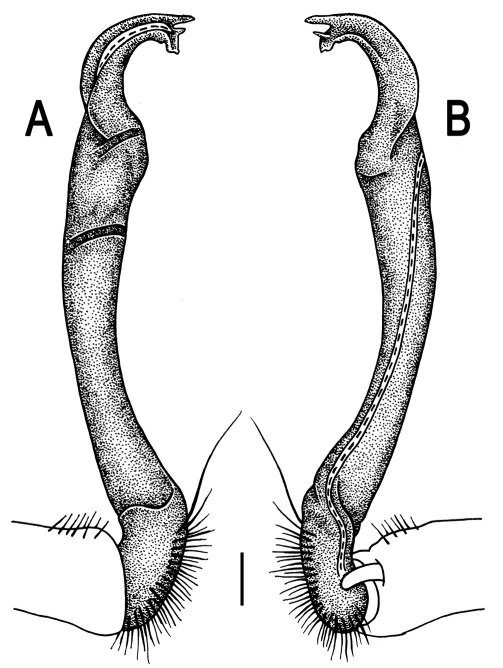
*Orthomorpha insularis* Pocock, 1895, ♂ from Doi Suthep National Park. **A, B** right gonopod, lateral and mesal views, respectively.Scale bar: 0.2 mm.

**Figure 95. F95:**
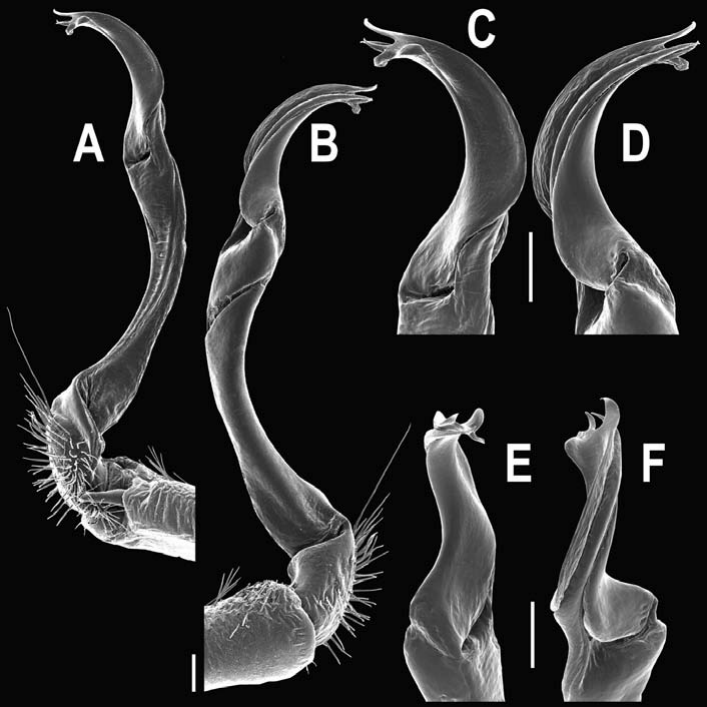
*Orthomorpha insularis* Pocock, 1895, ♂ from Thong Pha Phum. **A, B** right gonopod, mesal and lateral views, respectively **C-F** distal part of right gonopod, mesal, lateral, suboral and subcaudal views, respectively. Scale bar: 0.2 mm.

**Figure 96.**
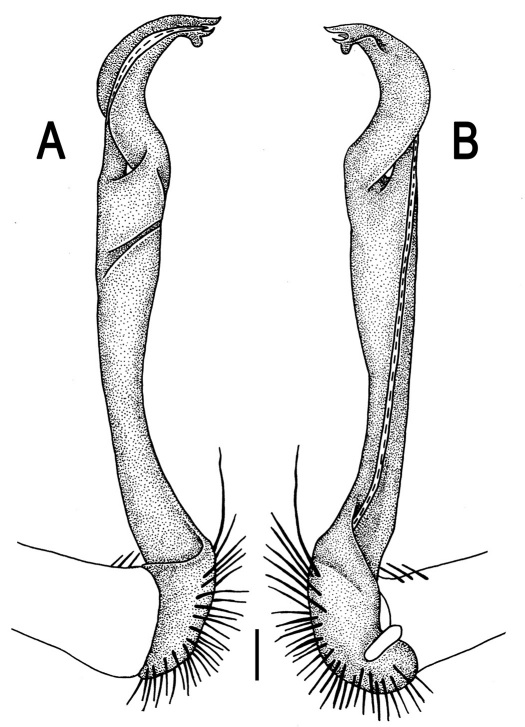
*Orthomorpha insularis* Pocock, 1895, ♂ from Krienkravia Waterfall. **A, B** right gonopod, lateral and mesal views, respectively.Scale bar: 0.2 mm.

Tip of gonopod trifid, with terminal lobule longest, middle spiniform prong sometimes being shorter than usual (Figs 93–96).

#### Remarks.

Jeekel (1970), when redescribing *Orthomorpha insularis* Pocock, 1895, from material from Myanmar, also emphasized clear variation in the width of the calluses and of the outlines of some paraterga. This has allowed him to synonymize several other nominate congeners, revised from type material from Myanmar, with *Orthomorpha insularis* (see catalogue section above). The above abundant material from Thailand confirms a profound variation range in size, coloration, shapes of the collum and following paraterga etc. This variation is deemed to be only individual, as our examination of larger series shows. No complete redescription is provided here, because that of Jeekel (1970) is detailed enough.

This species appears to be widespread over much of Thailand, as well as in the adjacent parts of Myanmar (Map 4). Enghoff (2005), Decker (2010) and Likhitrakarn et al. (2010a), in their faunistic accounts on Thai millipedes, mistakenly referred to *Orthomorpha intercedens*, which is long known to be only a junior synonym of *Orthomorpha insularis* (see Jeekel 1970).

### Orthomorpha crucifer

(Pocock, 1889)

Paradesmus crucifer Pocock 1889: 293 (D).
Orthomorpha crucifera – Attems 1898: 111 (D); 1914: 238 (M); 1936: 204 (M); 1937: 93 (M); Jeekel 1968: 53 (M); Golovatch 1998: 42 (D).

#### Remarks.

This species still remains dubious (Golovatch 1998), because it is based on a ♀ holotype (Pocock 1889).Although it could be incorporated in our key below, a redescription of ♂ topotypic material is necessary to confirm the species status of *Orthomorpha crucifer.* At present it is only known from King Island, Mergui Archipelago, Myanmar (Pocock 1889).

### Orthomorpha karschi

(Pocock, 1889)

Paradesmus Karschi Pocock 1889: 293 (D).
Orthomorpha karschii – Pocock 1895: 817 (D); Attems 1936: 197 (D); 1937: 70 (D); Jeekel 1963: 265 (M); 1964: 361 (M, D); Hoffman 1977: 700 (M).
Orthomorpha Karschii – Attems 1898: 327 (D).
Orthomorpha Karschi – Attems 1898: 333 (D); 1914: 192 (M, D).
Orthomorha karschi – Jeekel 1970: 290 (D); Jeekel 1968: 56 (M); Hoffman 1973: 363 (M); Golovatch 1998: 42 (D).

#### Remarks.

This species has been beautifully redescribed by Jeekel (1970) from non-type ♀ material from southern Tenasserim (= Tanintharyi), Myanmar, referred to by Pocock (1895). Based on the illustration of a gonopod by Attems (1937), *Orthomorpha karschi* appears to be very similar to *Orthomorpha insularis*, apparently differing only in a slightly shorter gonofemorite (Jeekel 1970). However, Pocock’s (1889) original drawing shows the femorite to be as slender as in *Orthomorpha insularis*. There is only a single micropreparation in the NHMW collection containing the gonopod depicted by Attems (1937).

This species seems to be endemic to southern Myanmar together with the adjacent Mergui Archipelago and Thailand. Attems (1937) referred to material from Siam (= Thailand), without giving a precise locality, but Enghoff (2005), in his catalogue of the Thai millipedes, must have overlooked that record.

### Orthomorpha conspicua

(Pocock, 1894)

Strongylosoma conspicuum Pocock 1894: 368 (D).
Orthomorpha conspicua – Attems 1898: 339 (M); 1907: 83 (M); Jeekel 1963: 265 (M); 1964: 360 (M, D); 1968: 45 (M); Hoffman 1973: 362 (M); 1977: 700 (M); Golovatch 1997b: 73 (D); 1998: 42 (D).
Strongylosoma conspicuum – Attems 1914: 235 (M); 1937: 280 (D).

#### Remarks.

This species has been adequately redescribed and illustrated from a ♂ syntype by Golovatch (1997b), thus requiring no further revision. *Orthomorpha conspicua* is only known from Bogor, Java, Indonesia (Pocock 1894).

### Orthomorpha weberi

(Pocock, 1894)

Strongylosoma weberi Pocock 1894: 367 (D).
Orthomorpha Weberi – Attems 1898: 339 (M); 1907: 83 (M); 1914: 192 (M, D).
Orthomorpha weberi – Attems 1930a: 127 (D); 1937: 69 (D); Jeekel 1963: 265 (M); 1964: 360 (M, D); 1968: 45 (M); Hoffman 1973: 362 (M); 1977: 700 (M); Golovatch 1997b: 75 (D); 1998: 42 (D).

#### Remarks.

This species has been adequately redescribed and illustrated from a ♂ syntype by Golovatch (1997b), thus requiring no further revision. *Orthomorpha weberi* is only known from Bogor, Java, Indonesia (Pocock 1894).

### Orthomorpha fuscocollaris

Pocock, 1895

Orthomorpha fusco-collaris Pocock 1895*:* 822 (D).
Orthomorpha fuscocollaris – Attems 1898: 339 (M); 1914: 194 (M); 1936: 205 (M); 1937: 94 (M); Jeekel 1964: 359 (M); 1963: 265 (M); 1968: 56 (M); 1970*:* 299 (D); Golovatch 1998: 42 (D).
Strongylosoma fuscocollaris – Sinclair 1901: 519 (R). [?Non]

#### Remarks.

This species has been adequately redescribed and illustrated from the ♀ lectotype by Jeekel (1970), thus making it possible to incorporate into our key below. To confirm its species status, however, revision of ♂ topotypic material is necessary. *Orthomorpha fuscocollaris* is only known from Malewoon (10°14' N, 98°37' E), Myanmar (Pocock 1895).

### Orthomorpha flaviventer

(Attems, 1898)

Prionopeltis flaviventer Attems 1898*:* 355 (D).
Prionopeltis flaviventer – Attems 1914*:* 203 (M).
Pratinus flaviventer – Attems 1937*:* 116 (D).
Orthomorpha flaviventer – Jeekel 1963: 265 (M); 1964: 360 (M, D); 1968: 45 (M); Hoffman 1973: 362 (M); 1977: 700 (M); Golovatch 1997b: 75 (D); 1998: 42 (D).

#### Remarks.

This species seems to have been described from a single ♂ (holotype) (Attems 1898), but is currently represented in the NHMW collection only by a micropreparation with a mounted gonopod. *Orthomorpha flaviventer* is only known from Preanger, Bandung, western Java, Indonesia (Attems 1898).

### Orthomorpha bipunctata

(Sinclair, 1901)

Strongylosoma bipunctatum Sinclair 1901: 519 (D).
Strongylosoma bipunctatum – Attems 1937: 280 (M).
Orthomorpha bipunctata – Attems 1914: 192 (M); 1930a: 128 (D); Jeekel 1964: 359 (M); 1968: 45 (M); Hoffman 1977: 700 (M, D); Golovatch 1998: 42 (D).

#### Remarks.

This species has been redescribed and illustrated from the ♂ lectotype coming from Gunong Inas, Perak State and two ♀ paralectotypes stemming from Kuala Aring, Kelantan State, Malaysia (Hoffman 1977). It still is known only from the type series taken from Malay Peninsula within Malaysia (Sinclair 1901).

### Orthomorpha francisca

Attems, 1930

Orthomorpha francisca Attems 1930b: 127 (D).
Orthomorpha francisca – Attems 1937: 69 (D); Jeekel 1963: 265 (M); 1964: 360 (M, D); 1968: 37 (M); Hoffman 1973: 362 (M); 1977: 700 (M); Golovatch 1997b: 75 (D); 1998: 42 (D).

#### Remark.

This species has been described from the ♂ holotype (Attems 1930b), still known only from the type locality: Narmada, western Lombok, Indonesia.

### Orthomorpha sericata

Jeekel, 1964

Orthomorpha sericata Jeekel 1964: 355 (D).
Orthomorpha sericata – Jeekel 1968: 56 (M); Golovatch 1998: 42 (D); Enghoff 2005: 98 (M, D); Likhitrakarn et al. 2010a: 38 (D).

#### Remark.

This species has recently been redescribed in due detail from the type series taken from Wat Son (Son Temple), near Bandon River, Kanchanadit District, Surat Thani Province, Thailand (Likhitrakarn et al. 2010a).

### Orthomorpha baliorum

Golovatch, 1995

Orthomorpha baliorum Golovatch 1995: 128 (D)
Orthomorpha baliorum – Golovatch 1997b: 79 (D); 1998: 42 (D).

#### Remark.

This species has been adequately described from Kebun Raya, Bali, Indonesia (Golovatch 1995), so it requires no redescription.

### Orthomorpha beroni

Golovatch, 1997

Orthomorpha beroni Golovatch 1997b: 76 (D).
Orthomorpha beroni – Golovatch 1998: 42 (D).

#### Remark.

This species has been adequately described from Pass Puncak, 34 km from Bogor, Java, Indonesia (Golovatch 1997b).

### Orthomorpha subsericata

Golovatch, 1998

Orthomorpha subsericata Golovatch 1998: 35 (D).
Orthomorpha subsericata – Enghoff 2005: 98 (M, D);Likhitrakarn et al. 2010a: 30 (D); Decker 2010: 32 (R).

#### Remark.

This species has recently been redescribed both from the type series and new material coming from the northwestern coast of the Gulf of Siam, Thailand (Likhitrakarn et al. 2010a).

### Orthomorpha alutaria

Likhitrakarn, Golovatch & Panha, 2010

Orthomorpha alutaria Likhitrakarn, Golovatch & Panha 2010a: 32 (D).

#### Remark.

This species has recently been described from several places in west-central Thailand (Likhitrakarn et al. 2010a).

### Orthomorpha asticta

Likhitrakarn, Golovatch & Panha, 2010

Orthomorpha asticta Likhitrakarn, Golovatch & Panha 2010a: 33 (D).

#### Remark.

This species has recently been described from several places in southern Thailand and the adjacent parts of Malaysia (Likhitrakarn et al. 2010a).

### Orthomorpha enghoffi

Likhitrakarn, Golovatch & Panha, 2010

Orthomorpha alutaria Likhitrakarn, Golovatch & Panha 2010a: 24 (D).

#### Remark.

This species has recently been described from several places in northern and central Thailand (Likhitrakarn et al. 2010a).

### Orthomorpha parasericata

Likhitrakarn, Golovatch & Panha, 2010

Orthomorpha parasericata Likhitrakarn, Golovatch & Panha 2010a: 35 (D).

#### Remark.

This species has recently been described from a single locality in the central part of Malay Peninsula within Thailand (Likhitrakarn et al. 2010a).

### Orthomorpha isarankurai sp. n.

urn:lsid:zoobank.org:act:CEAACB25-04F5-4AC1-9CC2-45A7EEBC0C09

http://species-id.net/wiki/Orthomorpha_isarankurai

**Figure 97.**
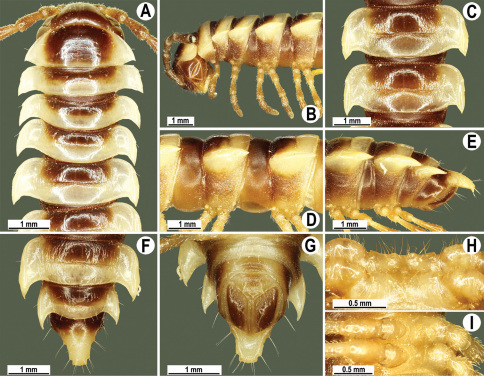
*Orthomorpha isarankurai* sp. n., ♂ holotype. **A, B** anterior part of body, dorsal and lateral views, respectively **C, D** segments 10 and 11, dorsal and lateral views, respectively **E–F** posterior part of body, lateral, dorsal and ventral views, respectively **H, I** sternal cones between coxae 4, subcaudal and sublateral views, respectively.

Figs 97 [Fig F98] 99

#### Holotype.

♂ (CUMZ), Thailand, Srakaeo Prov., Khao Chakan Distr., Khao Sam Sip, 13°40'36"N, 102°09'11"E, 03.09.2006, leg. S. Panha.

#### Paratypes.

2 ♂, 2 ♀ (ZMUC), 2 ♂, 2 ♀ (ZMUM), 4 ♂, 13 ♀, 2 juv. (CUMZ), same locality, together with holotype. 3 ♂, 1 ♀ (CUMZ), Chonburi Prov., Mueang Chon Buri Distr., Ang Hin Station, 13°20'21"N, 100°55'29"E, 01.11.1962, leg. K. Isarankura. 2 ♂, 1 ♀, 1 juv. (CUMZ), Chachengsao Prov., Phanom Sarakham Distr., Khao Hin Sorn, 04.09.2006, leg. C. Sutcharit. 1 ♂ (CUMZ), same Prov., Kaeng Hang Maeo Distr., Khao Sip Ha Chan National Park, 30.09.2009, leg. C. Sutcharit. 1 ♂ (CUMZ), Chanthaburi Prov., Mueang Chanthaburi Distr., Krathing Waterfall, ca 30 m, 16.09.2009, leg. N. Likhitrakarn.

#### Name.

Honours to Dr. Kumpol Isarankura, Professor of the Department of Biology of Chulalongkorn University, who collected some of the type specimens.

#### Diagnosis.

Differs in a peculiar colour pattern, coupled with narrow calluses of paraterga, and very broadly separated cones between ♂ coxae 4 (see also Key below).

#### Description.

Length mostly 29–34 (♂) or 33–36.5 mm (♀), width of midbody pro- and metazona 2.45–2.75 and 3.8–4.35 mm (♂), 2.9–3.35 and 4.35–4.85 mm (♀), respectively. One ♂ paratype (from Krathing Waterfall), 39.5 mm long, 3.6 and 5.5 mm wide on midbody pro- and metazona, respectively.

Coloration of alcohol material after long-term preservation castaneous brown with a pattern of contrasting whitish to yellow paraterga and epiproct, and mostly greyish-white posterior halves of postcollum metaterga; head and antennomeres 6 and 7 brown to dark brown; venter and a few basal podomeres light brown to yellow-brown, legs growing infuscate (brown) distally; tip of antenna pallid (Fig. 97A-G).

Clypeolabral region densely setose, vertex sparsely so, epicranial suture distinct. Antennae moderately long (Fig. 97B), surpassing end of body segment 3 (♂) or 2 (♀) dorsally. Head in width < collum < segment 4 < 2 = 3 < 5–16 (♂, ♀); thereafter body gently and gradually tapering. Collum with three transverse rows of setae: 3+3 anterior, 2+2 intermediate, and 2+2 posterior; a very faint incision laterally in posterior 1/3; caudal corner of paraterga pointed, beak-shaped, slightly declined ventrad, extended posteriad, but not surpassing rear tergal margin (Fig. 97A & B). Tegument smooth and shining, prozona very finely shagreened, metaterga smooth and delicately rugulose, leathery; surface below paraterga finely microgranulate. Postcollum metaterga with an anterior transverse row of 2+2, often abraded setae traceable at least as insertion points, a posterior row of 4+4 setae visible only on segment 19. Tergal setae long, slender, about 1/3 metatergal length. Axial line faint, barely traceable on metaterga (♂). Paraterga very strongly developed (Fig. 97A-G), especially well so in ♂, usually slightly upturned to subhorizontal, all lying high (at about 1/3 midbody height), albeit below dorsum; caudal corner almost completely to fully pointed, bent posteriad, especially strongly so on segments 16–18, either clearly (♂) or only very slightly extending beyond rear tergal margin (♀); paraterga very thin in lateral view, like blunt blades, a little thicker only on pore-bearing segments. Calluses delimited by a sulcus only dorsally, thin, especially so on poreless segments. Paraterga 2 broad, anterior edge straight, lateral edge with 2–3 small, but evident incisions in anterior 1/3; posterior edge slightly concave (Fig. 97B & C). Anterior edges of paraterga 3 and 4 evidently convex, of paraterga 5–18 nearly straight and slightly bordered. Lateral edge of paraterga 5–18 with a slight, but more evident incision/tooth in anterior 1/3 and a slightly smaller incision in posterior 1/3 only on pore-bearing segments. Posterior edge of paraterga evidently concave, usually bare, surface without traces of a lobule, on poreless paraterga more concave (Fig. 97A-D). Ozopores evident, lateral, lying in an ovoid groove at about 1/4 in front of caudal corner. Transverse sulcus usually very distinct (Fig. 97A, C & F), slightly incomplete on segments 4, 18 and 19 (not reaching bases of paraterga), complete on metaterga 5–17, deep, usually reaching bases of paraterga, at most faintly beaded at bottom, a little better developed in ♀. Stricture between pro- and metazona narrow, evidently beaded at bottom down to base of paraterga (Fig. 97A-F). Pleurosternal carinae complete crests with a sharp caudal tooth on segments 2–4, a small, caudal, mostly sharp tooth on segments 5–7 (Fig. 97B, D & E), a very faint tubercle on segments 8–12 (♂) or 5–9 (♀). Epiproct (Fig. 97E-G) conical, flattened dorsoventrally, with two evident apical papillae, less being especially clear in ♂; tip subtruncate; pre-apical papillae evident, lying close to tip. Hypoproct (Fig. 97G) roundly subtriangular, setiferous knobs at caudal edge well-separated and evident.

Sterna delicately and sparsely setose, without modifications, but with a pair of very small, blunt, fully separated cones between ♂ coxae 4 (Fig. 97I & J). A pair of conspicuous rounded tubercles flanking anterior edge of gonopod aperture. Legs moderately long and slender, slightly incrassate in ♂, midbody ones ca 1.4–1.5 (♂) or 1.2–1.3 times (♀) as long as body height, prefemora without modifications, ♂ tarsal brushes present until legs of segment 10.

**Figure 98. F98:**
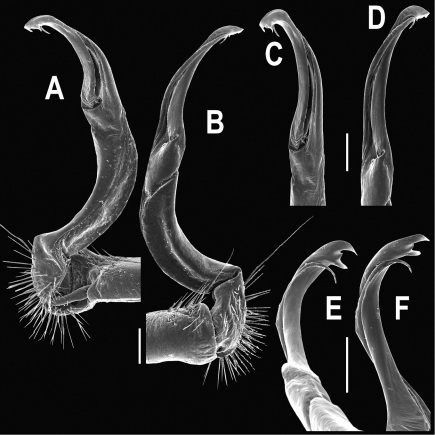
*Orthomorpha isarankurai* sp. n., ♂ holotype. **A, B** right gonopod, mesal and lateral views, respectively **C-F** distal part of right gonopod, mesal, lateral, suboral and subcaudal views, respectively. Scale bar: 0.2 mm.

**Figure 99.**
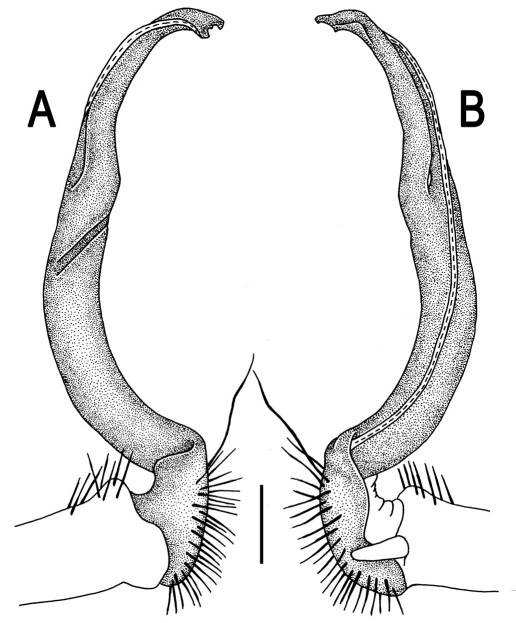
*Orthomorpha isarankurai* sp. n., ♂ holotype. **A, B** right gonopod, lateral and mesal views, respectively.Scale bar: 0.2 mm.

Gonopods (Figs 98 & 99) simple. Coxa long and slender, with several strong setae distodorsally. Prefemur densely setose, about half the length of femorite + “postfemoral” part. Femorite slender, curved and not enlarged distad, with a “postfemoral” part demarcated by an oblique lateral sulcus. Solenophore with a tridentate tip, middle denticle being especially small; solenomere long, flagelliform, a short tip exposed.

#### Remark.

This new species is rather widespread in eastern Thailand (Map 2).

### Orthomorpha elevata sp. n.

urn:lsid:zoobank.org:act:DDACEC9B-1E7D-483E-9B4D-F65E619D2E76

http://species-id.net/wiki/Orthomorpha_elevata

**Figure 100.**
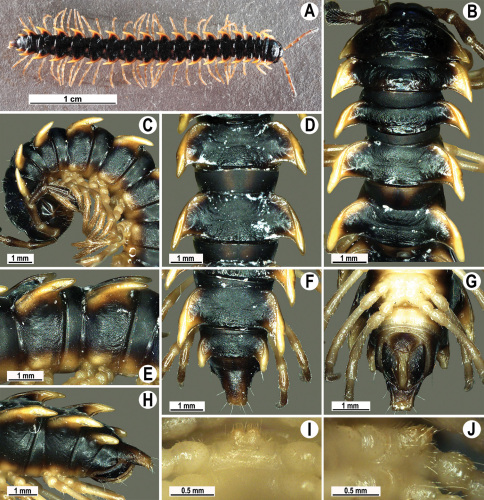
*Orthomorpha elevata* sp. n., ♂ holotype. **A** habitus, live coloration **B, C** anterior part of body, dorsal and lateral views, respectively **D, E** segments 10 and 11, dorsal and lateral views, respectively **F, G, H** posterior part of body, dorsal, ventral and lateral views, respectively **I, J** sternal cones between coxae 4, subcaudal and sublateral views, respectively.

Figs 100 [Fig F101] 102

#### Holotype.

♂ (CUMZ), Malaysia, Perak State, Bondig, Air Banum, ca 500 m, 5°38'13"N, 101°42'41"E, 27.05.2011, leg. S. Panha.

#### Paratypes.

2 ♀ (CUMZ), same data, together with holotype. 1 ♀ (CUMZ), Malaysia, Johor State, Sungai Bantang, ca 100 m, 2°19'50"N, 103°09'45"E, 21.05.2011, leg. R. Chanabun. 1 ♀ (CUMZ), same State, Sungai Bantang, ca 100 m, 2°19'50"N, 103°09'45"E, 21.05.2011, leg. R. Chanabun. 2 ♀ (CUMZ), Kelantan State, near Gua Pulai, ca 120 m, 4°74'36"N, 101°56'32"E, 26.05.2011, leg. R. Chanabun. 2 ♀ (CUMZ), Kelantan State, near Gua Matu Madu, ca. 130 m, 4°50'13"N, 101°56'56"E, 26.05.2011, leg. R. Chanabun.

#### Name.

To emphasize the mostly strongly elevated paraterga.

#### Diagnosis.

Differs by mostly strongly elevated paraterga, coupled with two small sternal cones between ♂ coxae 4 (see also Key below).

#### Description.

Length 34 (holotype) or 31–38 mm (♀), width of midbody pro- and metazona 2.8 and 4.3 mm (holotype), 2.9–3.5 and 4.3–5.0 mm (♀), respectively.

Coloration of live material (Fig. 100A) blackish to blackish-brown with contrasting pinkish paraterga legs, and tip of epiproct; coloration of alcohol material after long-term preservation blackish-brown with contrasting light yellowish calluses; two paramedian spots divided by a broad, blackish, axial stripe on prozona; lateral parts of paraterga near calluses, tip of epiproct, legs and venter light yellow-brown, legs slightly infuscate distally; antennae brown, distal part of antennomere 5, distal 2/3 of antennomere 6 and entire antennomere 7 blackish-brown (Fig. 100B-H).

Clypeolabral region densely setose, vertex bare, epicranial suture distinct. Antennae short, slightly clavate (antennomere 6 broadest), extending behind body segment 3 (♂) or 2 (♀) dorsally. Head in width < collum < segments 3 and 4 < 2 < 5–17 (♂, ♀); thereafter body gently and gradually tapering. Collum with three transverse rows of medium-sized setae, pattern untraceable; paraterga slightly declivous, subtriangular, with a faint lateral incision near midway, evidently discontinuing dorsum’s convexity, caudal corner pointed, about level to rear tergal margin (Fig. 100B & C). Tegument rather poorly shining, metaterga, prozona and most of metazona below paraterga finely shagreened; metaterga dull, leathery, roughly shagreened, their caudal margin evidently ribbed, with 3+3 or 4+4 short wrinkles; surface below paraterga rugulose, on segments 2–4(5) microgranulate. Postcollum metaterga with fully abraded setae, setation pattern untraceable even as insertion points. Axial line visible on collum and both on following pro- and metazona. Paraterga very strongly developed (Fig. 100A-H), especially well so in ♂, set at about 1/4 body height, postcollum ones upturned in lateral view, moderately enlarged on pore-bearing segments, thinner on poreless ones; shoulders broadly rounded and narrowly bordered, fused to callus; paraterga 2 and 19 (♂) or 19 (♀) lying below dorsum, paraterga 3 (♂) or 2–4 and 18 (♀) level to dorsum, remaining postcollum paraterga clearly above dorsum; caudal corner of postcollum paraterga pointed or narrowly rounded, always extending beyond rear tergal margin, best developed on segments 17 and 18 (Fig. 100F & G). Calluses delimited by a sulcus both dorsally and ventrally, with an evident front denticle only on paraterga 2, lateral margin virtually unincised on following paraterga, except for a small sinuosity in front of ozopore on pore-bearing segments. Posterior edge of paraterga strongly concave, especially strongly so on segments 18 and 19. Ozopores evident, lateral, lying in an ovoid groove at about 1/4 in front of caudal corner. Transverse sulcus complete on metaterga 4–18, narrow, superficial, not reaching bases of paraterga. Stricture between pro- and metazona narrow, rather deep, beaded at bottom down to base of paraterga (Fig. 100B-F & H). Pleurosternal carinae complete crests with a caudal tooth on segments 2–4, thereafter increasingly strongly reduced and remaining visible only as a front bulge and a caudal tooth until segment 17 (♂) or 13 (♀). Epiproct (Fig. 100F-H) conical, flattened dorsoventrally, with two small teeth directed ventrocaudally, emarginate or subtruncate at tip; pre-apical papillae small denticles lying very close to tip. Hypoproct (Fig. 100G) semi-circular, caudal margin with two strong and well-separated setiferous knobs.

Sterna sparsely setose, shining, without modifications; cross-impressions shallow; a paramedian pair of rather small, but evident tubercles in front of gonopod aperture; a small central lobe with a paramedian pair of evident, setose, apical cones between ♂ coxae 4 (Fig. 100I & J). Legs long and slender, slightly incrassate in ♂, midbody ones ca 1.3–1.4 (♂) or 1.1–1.2 times (♀) as long as body height, prefemora without modifications; ♂ tarsal brushes visible on legs 1–3, thereafter gradually thinning out.

**Figure 101. F101:**
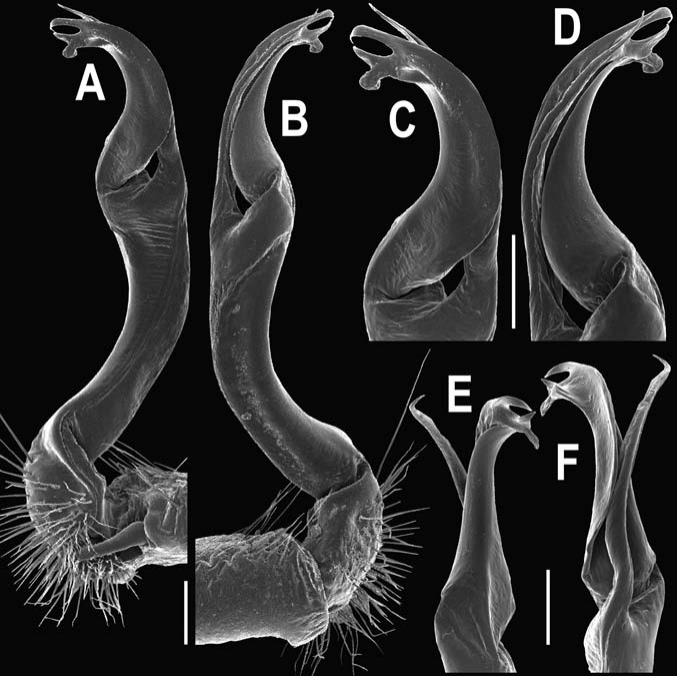
*Orthomorpha elevata* sp. n., ♂ holotype. **A, B** right gonopod, mesal and lateral views, respectively **C-F** distal part of right gonopod, mesal, lateral, suboral and subcaudal views, respectively. Scale bar: 0.2 mm.

**Figure 102.**
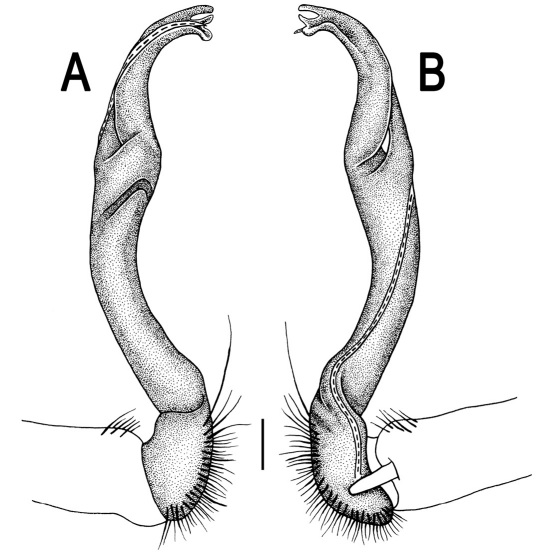
*Orthomorpha elevata* sp. n., ♂ holotype. **A, B** right gonopod, lateral and mesal views, respectively. Scale bar: 0.2 mm.

Gonopods (Figs 101 & 102) simple. Coxa long and slender, with several strong setae distodorsally. Prefemur densely setose, more than 2 times shorter than femorite + “postfemoral” part. Femorite slightly curved, slender, nearly not enlarged distad, with a “postfemoral” part demarcated by an oblique lateral sulcus. Solenophore tip distinctly trifid, terminal prong being sharp and longest, middle denticle nearly as long as subterminal lobule; solenomere long and flagelliform.

#### Remark.

This new species is rather widespread in northern Malaysia (Map 2).

### Orthomorpha subelevata sp. n.

urn:lsid:zoobank.org:act:75C015CB-D0D4-4745-8B4D-4FF1901F0892

http://species-id.net/wiki/Orthomorpha_subelevata

**Figure 103.**
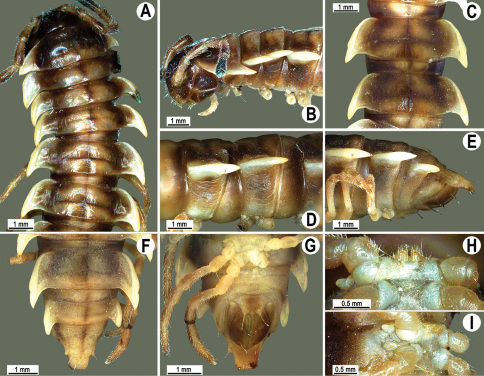
*Orthomorpha subelevata* sp. n., ♂ holotype. **A, B** anterior part of body, dorsal and lateral views, respectively **C, D** segments 10 and 11, dorsal and lateral views, respectively **D, E, F** posterior part of body, lateral, dorsal and ventral views, respectively **H, I** sternal cones between coxae 4, subcaudal and sublateral views, respectively.

Figs 103 [Fig F104] 105

#### Holotype.

♂ (CUMZ), Malaysia, Kedah State, Gunung Baling, 6°06'N, 101°29'E, 27.02.2008, leg. C. Sutcharit.

#### Paratypes.

2 ♀ (CUMZ), same data, together with holotype. 1 ♂ (CUMZ), Malaysia, Perlis State, Kaki Bukit, 7°04'32"N, 100°20'55"E, 28.03.2011, leg. R. Chanabun. 1 ♂, 1 ♀ (CUMZ), Penang State, Penang Dam, 5°41'08"N, 100°28'65"E, 26.03.2009, leg. C. Sutcharit. 1 ♂, 1 ♀, 1 juv. (CUMZ), Perak State, Gunung Genting, 4°35'58"N, 102°20'04"E, 30.03.2009, leg. C. Sutcharit.

#### Name.

To emphasize the strong similarity to *Orthomorpha elevata* sp. n.

#### Diagnosis.

Comes closest to *Orthomorpha elevata* sp. n., but differs in the paraterga lying below the dorsum, coupled with pleurosternal carinae present at least as a caudal denticle until segment 16 (♂) or 13 (♀) (see also Key below).

#### Description.

Length 40–42 (♂) or 41–45 mm (♀), width of midbody pro- and metazona 3.0–3.7 and 4.0–5.6 mm (♂), 3.0–4.2 and 4.9–6.1 mm (♀), respectively. Holotype 3.95 mm long, 3.9 and 4.0 mm wide on midbody pro- and metazona, respectively.

Coloration of alcohol material after long-term preservation brown to dark brown with contrasting whitish calluses, lateral parts of paraterga close to calluses and, sometimes, entire epiproct, as well as light yellow to yellow-brown legs and epiproct; two paramedian spots divided by a narrow, brown or grey-brown axial stripe on prozona and central parts of metaterga slightly lighter than background, light brown, except for an infuscate cross composed of transverse sulcus and axial line, the latter often extending from collum to tip of epiproct (Fig. 103A-G).

All other characters as in *Orthomorpha elevata* sp. n., except as follows.

Antennae barely reaching end of segment 3 (♂) or 2 (♀) dorsally. Ribs at caudal margin of metaterga often vague, sometimes represented by low bosses. Axial line always visible on collum, as well as on following prozona, metaterga and epiproct. Paraterga very strongly developed (Fig. 103A-G), but always lying below dorsum. Transverse sulcus incomplete on metatergum 4, complete, but not reaching bases of paraterga on metaterga 5–18. Hypoproct (Fig. 103G) subtrapeziform, caudal margin with two stronger and well-separated setiferous knobs.

Sterna densely setose. Legs long and slender, slightly incrassate in ♂, midbody ones ca 1.7–1.8 (♂) or 1.2–1.3 times (♀) as long as body height; ♂ tarsal brushes visible on legs 1–8, thereafter absent.

**Figure 104. F104:**
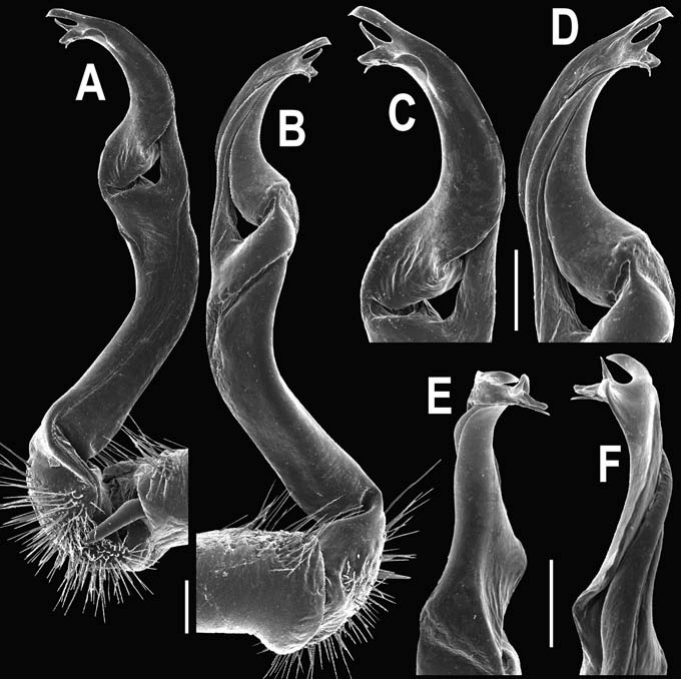
*Orthomorpha subelevata* sp. n., ♂ holotype. **A, B** right gonopod, mesal and lateral views, respectively **C–F** distal part of right gonopod, mesal, lateral, suboral and subcaudal views, respectively. Scale bar: 0.2 mm.

**Figure 105.**
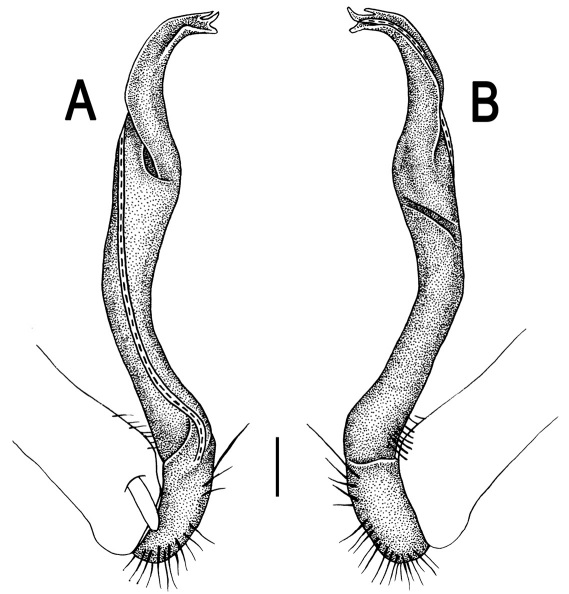
*Orthomorpha subelevata* sp. n., ♂ holotype. **A, B** left gonopod, mesal and lateral views, respectively.Scale bar: 0.2 mm.

Gonopods (Figs 104 & 105) virtually same as in *Orthomorpha elevata* sp. n.

#### Remark.

This new species is rather widespread in northern Malaysia (Map 2).

### Orthomorpha similanensis sp. n.

urn:lsid:zoobank.org:act:1D2A3461-01D5-4BF5-BDD7-A8DB221F9EE5

http://species-id.net/wiki/Orthomorpha_similanensis

**Figure 106.**
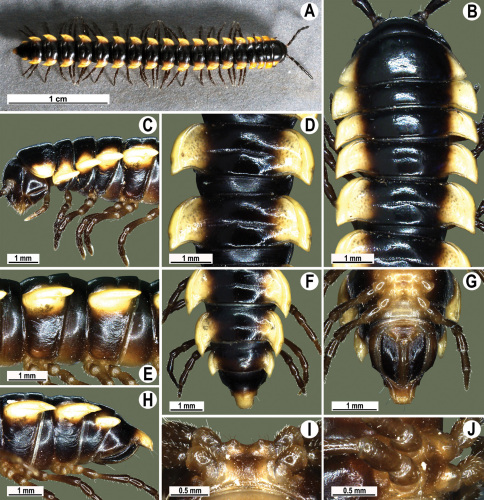
*Orthomorpha similanensis* sp. n., ♂ holotype. **A** habitus, live coloration **B, C** anterior part of body, dorsal and lateral views, respectively **D, E** segments 10 and 11, dorsal and lateral views, respectively **F, G, H** posterior part of body, dorsal, ventral and lateral views, respectively **I, J** sternal cones between coxae 4, subcaudal and sublateral views, respectively.

Figs 106 [Fig F107] 108

#### Holotype.

♂ (CUMZ), Thailand, Phang Nga Prov., Khura Buri Distr., Similan National Park, Ko Miang, Island 4, ca 40 m, 8°34'14"N, 97°38'14"E, 05.04.2010, leg. S. Panha, J. Sutcharit & N. Likhitrakarn.

#### Paratypes.

1 ♂, 1 ♀ (ZMUC), 1 ♂, 1 ♀ (ZMUM), 4 ♂, 3 ♀ (CUMZ), same locality, together with holotype.

#### Name.

To emphasize the type locality.

#### Diagnosis.

Differs by a strongly contrasting colour pattern, coupled with pleurosternal carinae represented by complete crests bulged anteriorly and with a sharp caudal tooth on segments 2–7, thereafter only a sharp caudal tooth on segments 8–16 (♂), or crests bulged anteriorly and with a sharp caudal tooth on segments 2–4, thereafter only a small sharp caudal tooth on segments 5–14 (♀) (see also Key below).

#### Description.

Length 26–30 (♂) or 31–33 mm (♀), width of midbody pro- and metazona 2.1–2.6 and 3.5–3.8 mm (♂), 2.9–3.4 and 4.0–4.3 mm (♀), respectively.

Coloration of live animals blackish, paraterga and epiproct contrastingly creamy yellow, legs and venter dark brown to blackish; coloration of alcohol material after long-term preservation dark brown to blackish, paraterga (marbled at base) and epiproct faded to pale yellow, legs and venter more pale brown (Fig. 106A-G).

Clypeolabral region densely setose, vertex sparsely so, epicranial suture distinct. Antennae moderately long (Fig. 106B), extending behind to body segment 3 (♂) or 2 (♀) dorsally. Head in width < collum < segment 3 = 4 < 2 = 5–16 (♂); thereafter body gently and gradually tapering. Collum with three transverse rows of setae: 3+3 anterior, 2+2 intermediate, and 2+2 posterior; lateral corner of paraterga narrowly rounded, slightly bordered and declined ventrally, not surpassing rear tergal margin (Fig. 106A & B). Tegument smooth and shining, prozona very finely shagreened, metazona leathery, delicately rugulose, below paraterga more evidently rugulose. Postcollum metaterga with an anterior transverse row of 2+2 setae. Tergal setae long, slender, about 1/3 metatergal length. Axial line very faint, barely traceable on metaterga (♂). Paraterga very strongly developed (Fig. 106A-H), especially well so in ♂, slightly upturned and lying below dorsum (at about 1/3 of midbody height); shoulders well-developed, slightly rounded and oblique laterally; caudal corner of paraterga 2 nearly pointed, thereafter increasingly narrowly pointed towards paraterga 17–19; paraterga bent posteriad, at least slightly extending beyond rear tergal margin, more evidently so on segments 2, 3 and 17–19. Calluses delimited by a sulcus only dorsally, rather narrow, a little wider on pore-bearing segments (Fig. 106B, D & F). Posterior edge of paraterga evidently concave, more strongly so on segments 16–19 (Fig. 106F-H). Ozopores evident, lateral, lying in an ovoid groove at about 1/4 in front of caudal corner. Transverse sulcus usually broad and shallow (Fig. 106B, D & F), superficial (especially so due to coarse texture around), slightly incomplete on segments 4 and 19, complete on metaterga 5–18, slightly not reaching bases of paraterga, a little better developed in ♀ (Fig. 106B, D & F). Stricture between pro- and metazona narrow, shallow, evidently beaded at bottom down to base of paraterga (Fig. 106B, D, E & F). Pleurosternal carinae complete crests bulged anteriorly and with a sharp caudal tooth on segments 2–7, thereafter only a sharp caudal tooth on segments 8–16 (♂), or crests bulged anteriorly and with a sharp caudal tooth on segments 2–4, thereafter only a small sharp caudal tooth on segments 5–14 (♀). Epiproct (Fig. 106F-H) conical, flattened dorsoventrally, with two evident apical papillae, subtruncate at tip; pre-apical papillae evident. Hypoproct (Fig. 106G) subtriangular, caudal margin pointed; setiferous knobs at caudal edge well-separated, rather small.

Sterna delicately and sparsely setose, without modifications, but with a pair of very small, blunt, fully separated cones between ♂ coxae 4 (Figs 106I & J). A pair of conspicuous rounded tubercles flanking anterior edge of gonopod aperture, both bent posterolaterad. Legs moderately long and slender, slightly incrassate in ♂, midbody ones ca 1.3–1.4 (♂) or 0.8–0.9 times (♀) as long as body height, prefemora without modifications, ♂ tarsal brushes present until legs of segment 13.

**Figure 107. F107:**
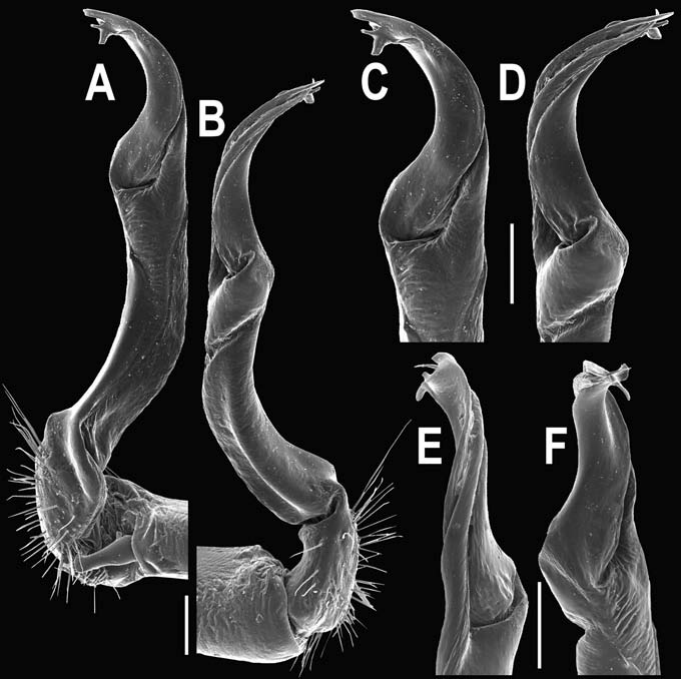
*Orthomorpha similanensis* sp. n., ♂ holotype. **A, B** right gonopod, mesal and lateral views, respectively **C–F** distal part of right gonopod, mesal, lateral, suboral and subcaudal views, respectively Scale bar: 0.2 mm.

**Figure 108.**
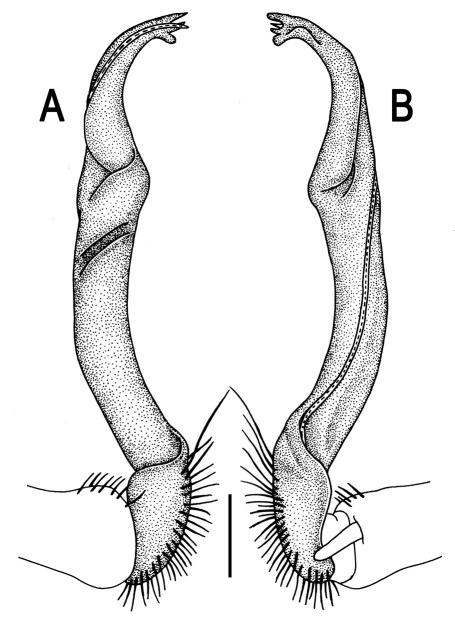
*Orthomorpha similanensis* sp. n., ♂ holotype. **A, B** right gonopod, lateral and mesal views, respectively.Scale bar: 0.2 mm**.**

Gonopods (Figs 107 & 108) simple. Coxa long and slender, with several strong setae distodorsally. Prefemur densely setose, about half the length of femorite + “postfemoral” part. Femorite slender, slightly curved and nearly not enlarged distad, with a “postfemoral” part demarcated by an oblique lateral sulcus. Solenophore with a tridentate tip, middle prong about as long as terminal denticle, subterminal lobule rounded, smallest; solenomere long, flagelliform, a short tip exposed.

#### Remark.

This new species is only known from one of the islets of Similan Archipelago, Thailand (Map 2).

### Orthomorpha spiniformis sp. n.

urn:lsid:zoobank.org:act:B6E4C181-A49C-4EA7-A5EB-0FA8B8B47C4C

http://species-id.net/wiki/Orthomorpha_spiniformis

**Figure 109.**
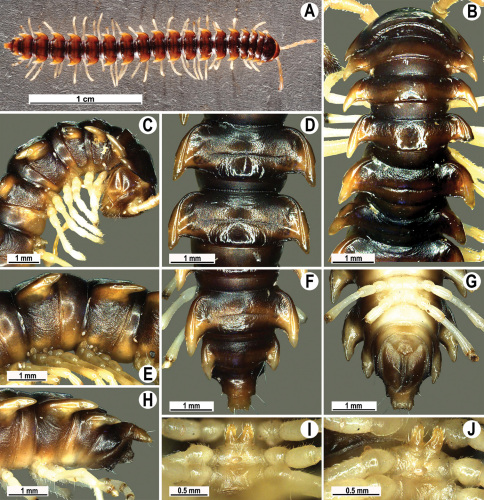
*Orthomorpha spiniformis* sp. n., ♂ holotype. **A** habitus, live coloration **B, C** anterior part of body, dorsal and lateral views, respectively **D, E** segments 10 and 11, dorsal and lateral views, respectively **F, G, H** posterior part of body, dorsal, ventral and lateral views, respectively **I, J** sternal cones between coxae 4, subcaudal and sublateral views, respectively.

Figs 109 [Fig F110] 111

#### Holotype.

♂ (CUMZ), Malaysia, Pahang State, Panang National Park, ca 80 m, 3°52'22"N, 102°19'20"E, 23.05.2011, leg. S. Panha.

#### Paratypes.

1 ♂, 1 ♀ (CUMZ), same State, Lake Kenyir, ca 170 m, 4°50'44"N, 102° 43'15"E, 25.05.2011, leg. S. Panha.

#### Name.

To emphasize the spiniform paraterga.

#### Diagnosis.

Differs in strongly spiniform paraterga, coupled with virtually smooth metaterga etc. (see also Key below).

#### Description.

Length 25–28 mm (♂), 29 mm (♀), width of midbody pro- and metazona 2.0–2.2 and 3.1–3.5 mm (♂), 2.4 and 3.5 mm (♀), respectively.

Coloration of live animals blackish-brown, calluses of paraterga (especially caudal parts) and distal half of epiproct contrasting red-brown; legs and venter yellow to light brown (Fig. 109A); coloration of alcohol material after long-term preservation faded to dark castaneous brown, paraterga contrasting pallid to very light brownish, antennomeres 1–4 very light brown, distal antennomeres brown (Fig. 109B-H).

Clypeolabral region densely setose, vertex bare, epicranial suture distinct. Antennae short, poorly clavate (Fig. 109A), extending behind (♂) or not reaching end of segment 2 (♀) dorsally. Head in width << collum < segment 3 = 4 < 2 < 5–17 (♂, ♀); thereafter body gently and gradually tapering. Collum semi-lunar, with an untraceable pattern of setation, surface rugulose, middle slightly flattened; paraterga narrowly subtriangular, subspiniform, devoid of lateral incisions; caudal corner nearly pointed, not extending behind tergal margin (Fig. 109B & C). Tegument rather poorly shining, prozona very finely shagreened, metaterga rugose to rugulose (especially well so near bases of paraterga), finely granulate, below paraterga microgranulate. Postcollum metaterga with two transverse rows of short, mostly abraded setae traceable only as insertion points or short wrinkles: 2+2 in front (pre-sulcus) row without wrinkles and 2+2 (or 3+3?) in caudal (postsulcus) row on wrinkles (Fig. 109B-F). Axial line rather clear, especially so on postsulcus halves of metaterga. Paraterga very strongly developed (Fig. 109A-H), especially well so in ♂, always spiniform and pointed, mostly subhorizontal to faintly upturned, level to or slightly above dorsum, only on segments 1–4 and 19 evidently below dorsum, set at about 1/4 midbody height; shoulders strongly and regularly rounded, well-developed, caudal corners of postcollum paraterga always extending beyond tergal margin. Calluses delimited by a sulcus both dorsally and ventrally, especially deeply so dorsally, rather narrow, with 3–4 small lateral incisions on callus 2 and one similarly unclear indentation on each following segment, these indentations lying at about front 1/3 on poreless segments and at about 1/2 on pore-bearing ones (Fig. 109B, D & F). Posterior edge of paraterga always very evidently concave, more strongly so on segments 16–19 (Fig. 109F). Ozopores evident, lateral, lying in a deep ovoid groove at about 1/3–1/4 paratergal length in front of caudal corner. Transverse sulcus present on metaterga 5–17, complete and reaching bases of paraterga, incomplete and faint on metatergum 18 (♂, ♀), beaded at bottom, rather deep (Fig. 109B, D & F). Stricture between pro- and metazona deep, narrow, evidently ribbed at bottom down to base of paraterga. Pleurosternal carinae poorly developed, especially so in ♀, as small, complete and roughly granulate crests with a distinct tooth both frontally and caudally only on segments 2–4, traceable as a small caudal denticle on segments 5–7 (♂) or 5 and 6 (♀) (Fig. 109C, E & H). Epiproct (Fig. 109F-H) conical, rather short, flattened dorsoventrally, with two very strong pre-apical papillae strongly removed from a deeply emarginate tip. Hypoproct (Fig. 109G) roundly subtrapeziform, setiferous knobs at caudal edge very small and well-separated.

Sterna shining, sparsely setose; a paramedian pair of evident, fully separated, anteroventrally directed, setose cones between ♂ coxae 4 (Fig. 109I & J), these being similar in size to those in front of gonopod aperture. Legs long and slender, almost not incrassate in ♂, midbody ones ca 1.6–1.7 (♂) or 1.1–1.2 times (♀) as long as body height, prefemora without modifications, ♂ tarsal brushes present only on ♂ legs 1–2, thereafter gradually thinning out.

**Figure 110. F110:**
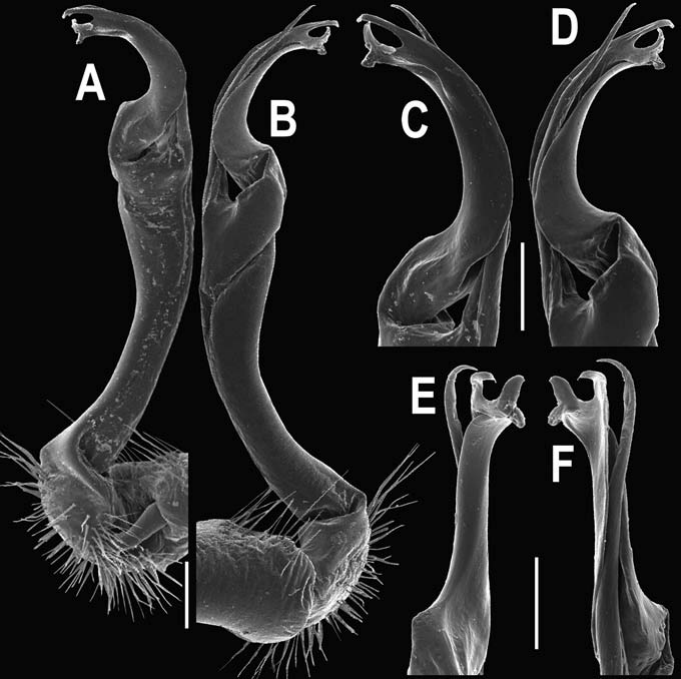
*Orthomorpha spiniformis* sp. n., ♂ paratype. **A, B** right gonopod, mesal and lateral views, respectively **C-F** distal part of right gonopod, mesal, lateral, suboral and subcaudal views, respectively. Scale bar: 0.2 mm.

**Figure 111.**
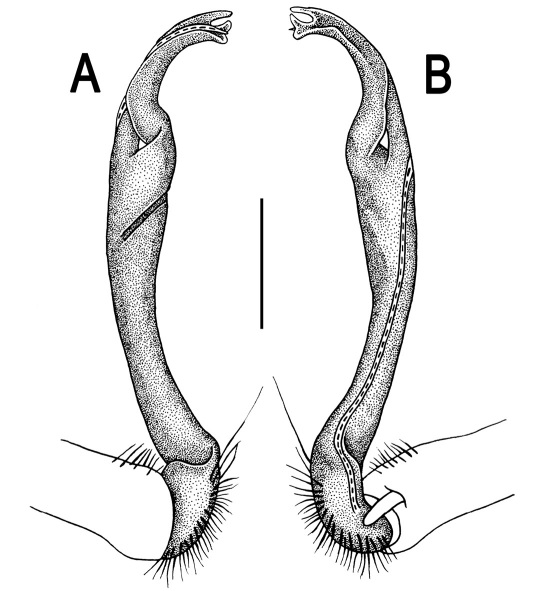
*Orthomorpha spiniformis* sp. n., ♂ holotype. **A, B** right gonopod, lateral and mesal views, respectively. Scale bar: 0.2 mm.

Gonopods (Figs 110 & 111) simple. Coxa long and slender, with several strong setae distodorsally. Prefemoral portion densely setose, about 3 times shorter than femorite + “postfemoral” part. Femorite slender, slightly curved, nearly not enlarged distad, with a “postfemoral” part demarcated by an oblique lateral sulcus. Solenophore tip clearly trifid, with a pronounced middle denticle; solenomere long and flagelliform.

#### Remarks.

This new species seems to be restricted to Pahang State, southern Malay Peninsula within Malaysia (Map 2).

##### Species with a shortened gonopod femorite

### Orthomorpha butteli

Carl, 1922

http://species-id.net/wiki/Orthomorpha_butteli

Figs 112 113

Orthomorpha butteli Carl 1922: 576 (D).
Orthomorpha consocius Chamberlin 1945: 11 (D), **syn. n.**
Pratinus butteli – Attems 1937: 121 (D).
Orthomorpha butteli – Jeekel 1963: 266 (M); 1964: 362 (M, D); 1968: 45 (M); Golovatch 1998: 42 (M).
Orthomorpha consocius – Jeekel 1963: 265 (M); 1964: 359 (M); 1968: 45 (M); Golovatch 1998: 42 (D).

#### Holotype.

♂ of *Orthomorpha consocius* (AMNH), Indonesia, Java Barat, Tjibodas, 07.1920, leg. Dammerman.

#### Redescription.

Length 25.5 mm, width of midbody pro- and metazona 2.2 and 3.2 mm, respectively.

**Figure 112.**
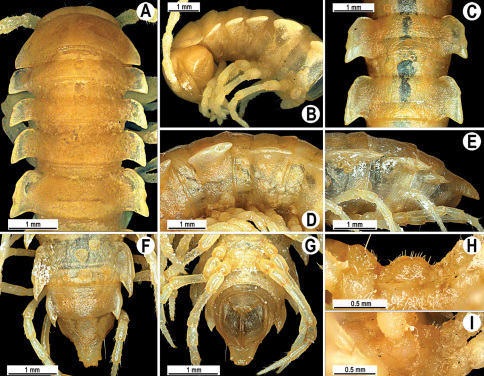
*Orthomorpha butteli* Carl, 1922, ♂ holotype of *Orthomorpha consocius* Chamberlin, 1945. **A, B** anterior part of body, dorsal and lateral views, respectively **C, D** segments 10 and 11, dorsal and lateral views, respectively **E-G** posterior part of body, lateral, dorsal and ventral views, respectively **H, I** sternal cones between coxae 4, subcaudal and sublateral views, respectively.

Coloration of alcohol material after long-term preservation yellowish-brown with contrasting pallid paraterga; venter, legs and antennae light yellowish (Fig. 112A-G).

Clypeolabral region densely setose, vertex sparsely so, epicranial suture distinct. Antennae moderately long, clavate (antennomere 6 broadest), extending behind body segment 3 dorsally (Fig. 112B). Head in width < collum < segment 3 = 4 < 2 < 5–16; thereafter body gently and gradually tapering. Collum semi-lunar, with three transverse rows of setae; 4+4 anterior, 1+1 intermediate and 3+3 posterior; caudal corner of paraterga subrectangular, very narrowly rounded, slightly declined ventrad, not surpassing rear tergal margin (Fig. 112A & B). Tegument dull, prozona very finely shagreened, metazona leathery, evidently rugulose, below paraterga microgranular and faintly rugulose. Postcollum metaterga with an anterior transverse row of 2+2 and a posterior of 3+3 insertion points. Axial line not traceable. Paraterga very well-developed (Fig. 112A-G), all lying below dorsum, set at about 1/3 body height, subhorizontal, in lateral view modestly enlarged on pore-bearing segments, thinner on poreless ones; shoulders always present, regularly rounded and narrowly bordered, fused to callus; caudal tips of all paraterga pointed, beak-like, extending increasingly beyond rear tergal margin, best developed and slightly curved mesad on segments 15–19. Calluses developed only dorsally. Paraterga 2 broad, front margin angulate and rounded, lateral margin with three small, but evident denticles, but only with two small incisions on following segments. Posterior edge of paraterga evidently concave, especially strongly so on segments 15–19. Ozopores evident, lateral, lying in an ovoid groove at about 1/3–1/4 in front of caudal corner. Transverse sulcus present on metaterga 5–18, broad, shallow, not reaching bases of paraterga (Fig. 112A, C & F). Stricture between pro- and metazona narrow and rather shallow, evidently beaded at bottom down to base of paraterga (Fig. 112A-F). Pleurosternal carinae like complete crests with a sharp caudal tooth on segments 2 and 3, as a low swelling on segment 4, thereafter missing (Fig. 112B & D). Epiproct (Fig. 112E-G) conical, flattened dorsoventrally, with two evident apical papillae directed ventrocaudally, slightly emarginate at tip; pre-apical papillae very evident, lying close to tip. Hypoproct (Fig. 112G) roundly subtriangular, setiferous knobs at caudal edge well-separated and small.

Sterna sparsely setose, without modifications; cross-impressions shallow; with an evident, paramedian, sparsely setose, directed anteroventrally, bulge between ♂ coxae 4, this bulge showing traces of axial division (Fig. 112H & I). Legs long and slender, midbody ones ca 1.3–1.4 times as long as body height, prefemora without modifications, ♂ tarsal brushes present only on legs 1–8.

**Figure 113.**
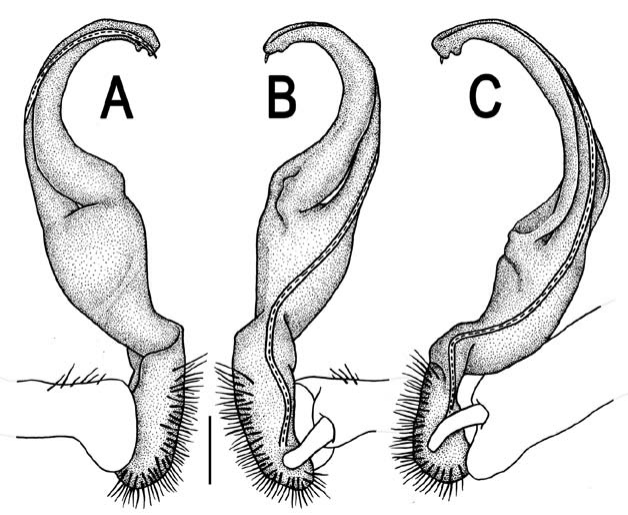
*Orthomorpha butteli* Carl, 1922, ♂ holotype of *Orthomorpha consocius* Chamberlin, 1945. **A, B, C** right gonopod, lateral, mesal and subcaudal views, respectively. Scale bar: 0.2 mm.

Gonopods (Fig. 113) simple. Coxa long and slender, with several strong setae distodorsally. Prefemur rather long, densely setose, about as long as femorite (+ “postfemoral” part). Femorite strongly enlarged, club-shaped, slightly curved, with only very faint traces of an oblique lateral impression. Solenophore with a somewhat folded base of lamina medialis; tip poorly bilobule, terminal lobule blunt, a little larger than inner one, each being supplied with a minute indentation near base; solenomere long, and flagelliform.

#### Remarks.

The original description of *Orthomorpha consocius* Chamberlin, 1945 being anecdotal (Chamberlin 1945), above is its redescription, based on the holotype coming from Tjibodas, Java, Indonesia. A comparison of the redescription with the original description of *Orthomorpha butteli* Carl, 1922, also from Tjibodas, leaves no doubt about their synonymy, because they coincide in virtually every detail, including gonopod structure. Hence the new synonymy advanced.

Concerning the status of *Orthomorpha butteli* Carl, 1922, because of its strikingly shortened gonopod femorite (Fig. 113), Golovatch (1998) preferred to exclude this species from *Orthomorpha*, even though (Jeekel (1963, 1968) had regarded it as a congener, albeit fairly disjunct. Now we are inclined to follow Jeekel, chiefly because all of the other characters in *Orthomorpha butteli*, including the very broad paraterga, are those of a typical *Orthomorpha*. So this time we include it into our key below.

##### Dubious species

### ? Orthomorpha herpusa

Attems, 1898

Fig. 114

Orthomorpha herpusa Attems 1898: 329 (D).
Orthomorpha herpusa – Attems 1907: 83 (M); 1914: 228 (M); 1937: 94 (M); Jeekel 1968: 42 (M).

#### Syntypes.

3 ♀ (NHMW-7985), Indonesia, eastern Java, “Tanger region” (= Tengger Mountains), 1893–1894, leg. D. Adensamer.

#### Redescription.

**Figure 114.**
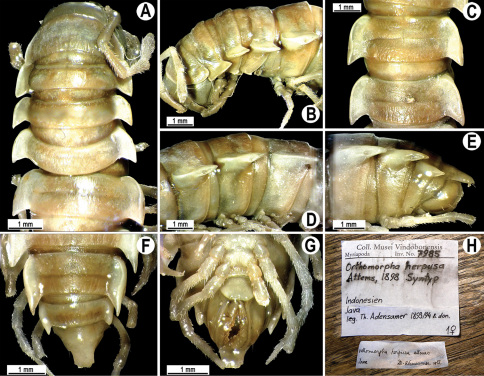
*Orthomorpha herpusa* Attems, 1898, ♀ syntype. **A, B** anterior part of body, dorsal and lateral views, respectively **C, D** segments 10 and 11, dorsal and lateral views, respectively **E-G** posterior part of body, lateral, dorsal and ventral views, respectively **H** samplelabels.

Length 26–30 mm, width of midbody pro- and metazona 3.2–3.5 and 3.8–4.4 mm, respectively (vs 30 mm in length and 3.2 mm in width, as given in the original description (Attems 1898)). Coloration of alcohol material upon long-term preservation light grey-brown with moderately contrasting yellowish paraterga (Fig. 114) (vs black-brown with moderately contrasting yellow-brown paraterga, as given in the original description (Attems 1898)).

Clypeolabral region densely setose, vertex sparsely so, epicranial suture distinct. Antennae rather long (antennomere 6 broadest), extending behind body segment 3 dorsally. Head in width < collum < segment 3 = 4 < 2 < 5–16; thereafter body gently and gradually tapering. Collum with three transverse rows of setae traceable only as insertion points: 3+3 anterior, 2+2 intermediate and 3+3 posterior; caudal corner of paraterga rounded, strongly declined ventrally and almost continuing collum’s convexity (Fig. 114A & B); paraterga subrectangular, not extending behind tergal margin, posterior edge nearly straight (Fig. 114A & B). Tegument smooth and poorly shining, prozona very finely shagreened, metazona leathery, slightly rugulose, below paraterga very faintly microgranular. Metaterga 2–19 with two transverse rows of setae: 2+2 in anterior (pre-sulcus) row and 3+3 in posterior (postsulcus) one, all abraded, but still traceable as insertion points. Axial line barely visible, starting from collum. Paraterga well developed (Fig. 114A–G), all lying below dorsum (at about 1/3 body height), mostly subhorizontal, in lateral view moderately strongly enlarged on pore-bearing segments, thinner on poreless ones; shoulders always present, broadly rounded and narrowly bordered, fused to callus; anterior edge of paraterga 2 straight, roundly angulate, following paraterga rounded, caudal corner of all paraterga extending increasingly beyond rear tergal margin, nearly pointed to pointed. Calluses delimited by a sulcus only dorsally, rather narrow, lateral edge mostly with one minute incision in front 1/3, only paraterga 2 with 2–3 similarly minute incisions. Posterior edge of paraterga evidently concave, especially strongly so on segments 14–19. Ozopores evident, lateral, lying in an ovoid groove at about 1/3 in front of caudal corner. Transverse sulcus complete on metaterga 5–18, incomplete on segments 4 and 19, very narrow, shallow, not reaching bases of paraterga, faintly beaded at bottom (Fig. 114A, C & F). Stricture between pro- and metazona narrow and shallow, faintly ribbed at bottom down to base of paraterga (Fig. 114A–F). Pleurosternal carinae complete crests with a sharp caudal tooth on segments 2–4, a small tooth caudally on segment 5, thereafter missing (Fig. 114B). Epiproct (Fig. 114E-G) conical, flattened dorsoventrally, with two small apical papillae directed more ventrad than caudally, slightly emarginate at tip; pre-apical papillae small, lying close to tip. Hypoproct (Fig. 114G) nearly semi-circular, caudal tip broadly rounded, setiferous knobs at caudal edge very small and moderately well separated.

Sterna sparsely setose, without modifications; cross-impressions rather deep. Legs moderately long and slender, midbody ones ca 1.1–1.2 times as long as body height.

#### Remarks.

This species has been described from a series of ♀ syntypes, still known only from the type locality: Tengger Mountains, eastern Java, Indonesia (Attems 1898).

In addition to *Orthomorpha herpusa*, as many as ten further species of *Orthomorpha* are currently known from Java: *Orthomorpha beaumontii*, *Orthomorpha hydrobiologica*, *Orthomorpha unicolor*, *Orthomorpha tenuipes*, *Orthomorpha zehntneri*, *Orthomorpha conspicua*, *Orthomorpha weberi*, *Orthomorpha flaviventer*, *Orthomorpha beroni* and *Orthomorpha coarctata*. Of them, only *Orthomorpha tenuipes* has also been described from Tengger Mountains, but it is evidently different from *Orthomorpha herpusa* in showing higher and laterally better incised paraterga with much broader calluses etc. (Fig. 49). Among the best candidates to match the description of *Orthomorpha herpusa* are perhaps *Orthomorpha beaumontii* (Figs 1 & 2), *Orthomorpha hydrobiologica* (Fig. 37), *Orthomorpha unicolor* (Fig. 39), and *Orthomorpha zehntneri* (Fig. 53), but still there are certain differences in ♀ somatic characters alone, often profound enough. So only a strict ♂ topotype is necessary to obtain and examine to ultimately establish the identity of *Orthomorpha herpusa*. Furthermore, this species might as well prove to belong to another genus of Orthomorphini, for instance *Nesorthomorpha* Jeekel, 1980, with eight species, all endemic to Java and often showing similarly strongly developed paraterga (Golovatch and Wytwer 2001).

### “Orthomorpha” crinita

Attems, 1900

Fig. 115

Orthomorpha crinita Attems 1900: 142 (D).
Orthomorpha (?) *crinita* – Mauriès 1980: 161 (M).
? Orthomorpha crinita – Golovatch & Korsós 1992: 29 (M); Golovatch & Gerlach 2010: 399 (D).

#### Syntypes.

2 ♀ (NHMW-7986), Seychelles, Mahé Island, primeval forest, 1895, leg. A. Brauer.

#### Descriptive notes.

**Figure 115.**
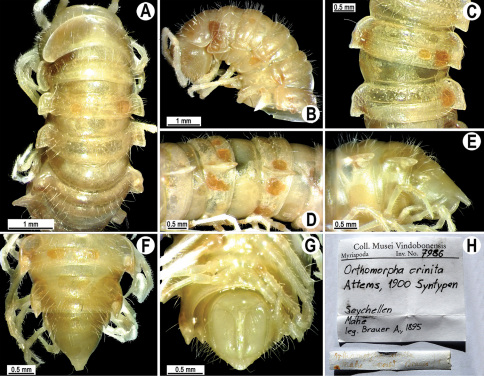
*Orthomorpha crinita* Attems, 1900, ♀ syntype. **A, B** anterior part of body, dorsal and lateral views, respectively **C, D** segments 10 and 11, dorsal and lateral views, respectively **E–G** posterior part of body, lateral, dorsal and ventral views, respectively **H** samplelabels.

Length 19–20 mm, width of midbody pro- and metazona 2.1–2.2 and 2.5–2.6 mm, respectively. Coloration of alcohol material upon long-term preservation uniformly light grey-brown (Fig. 115).

Metaterga densely and irregularly setose. All other somatic characters as in Fig. 115.

#### Remarks.

This species has been described from three ♀ syntypes, still known only from the type locality: Mahé Island, Seychelles (Golovatch & Gerlach 2010). Superficially, it differs readily from the only other paradoxosomatid endemic to the Seychelles, *Diglossosternoides curiosus* Golovatch and Korsós, 1992, tribe Eustrongylosomatini, in the remarkably densely setose collum and following metaterga (Golovatch and Korsós 1992).

Based both on the morphological characters (e.g. the narrow paraterga and densely setose metaterga) and distribution, there can be no doubt that *Orthomorpha crinita* has nothing to do with *Orthomorpha*.

### “Orthomorpha” variegata

Brölemann, 1896

Orthomorpha variegata Brölemann 1896: 333 (D).
Orthomorpha variegata – Brölemann 1904: 10 (D); Attems 1898: 339 (M); 1914: 238 (M); 1937: 94 (M); Jeekel 1968: 66 (M); Golovatch 1998: 42 (D); Enghoff et al. 2004: 39 (M); Enghoff 2005: 98 (M).

#### Remarks.

The paraterga in *“O.” variegata* seem to be too narrow to represent a species of *Orthomorpha*,as beautifully redescribed by Brölemann (1904) from the ♀ holotype. Golovatch (1998) included it into his key to *Orthomorpha* species for the sake of completeness. At present we are inclined to follow Jeekel (1968) and exclude this species from *Orthomorpha* altogether.

### “Orthomorpha” bisulcata

Pocock, 1895

Orthomorpha bisulcata Pocock 1895: 808 (D).
Orthomorpha bisulcata –Attems 1898: 338 (M); 1903: 64 (R); 1914: 237 (M); 1936: 204 (M); 1937: 93 (M); Jeekel 1968: 66 (M); Golovatch 1998: 42 (D).
“Orthomorpha” bisulcata – Jeekel 1965: 141 (D).

#### Remarks.

The paraterga in *Orthomorpha bisulcata* are definitely too narrow to represent a species of *Orthomorpha* (Pocock 1895; Jeekel 1965). Attems (1903) recorded it from Tjibodas, Java, Indonesia, apparently a misidentification. Both Attems (1937) and Jeekel (1968) referred to it as a species incertae sedis, because it was based only on ♀ material. It has been adequately redescribed by Jeekel (1965) from the ♀ lectotype coming from Rangoon, Myanmar. Golovatch (1998) included it into his key to *Orthomorpha* species for the sake of completeness. At present we are inclined to follow Jeekel (1968) and exclude this species from *Orthomorpha* altogether.

### “Orthomorpha” coxisternis

Pocock, 1895

Orthomorpha coxisternis Pocock 1895: 811 (D).
Orthomorpha coxisternis – Attems 1898: 339 (M); 1914: 238 (M); 1936: 204 (M); 1937: 93 (M); Jeekel 1968: 66 (M); Golovatch 1998: 42 (D).
“Orthomorpha” coxisternis – Jeekel 1965: 138 (D).

#### Remarks.

The paraterga in *Orthomorpha coxisternis* are definitely too narrow to represent a species of *Orthomorpha* (Pocock 1895; Jeekel 1965). Both Attems (1937) and Jeekel (1968) referred to it as a species incertae sedis, because it was based only on ♀ material. It has been adequately redescribed by Jeekel (1965) from the ♀ holotype coming from Bhamo, Myanmar. Even though Golovatch (1998) included it into his key to *Orthomorpha* species for the sake of completeness, at present we are inclined to follow Jeekel (1968) and exclude this species from *Orthomorpha* altogether.

### “Orthomorpha” oatesii

Pocock, 1895

Orthomorpha oatesii Pocock 1895: 821 (D).
Orthomorpha Oatesii – Attems 1898: 328 (D); 1914: 192 (M, D).
Orthomorpha oatesii – Attems 1936: 199 (M); 1937: 79 (D); Jeekel 1963: 265 (M). 1964: 359 (M); 1968: 56 (M); Golovatch 1998: 42 (D).

#### Remarks.

According to the original description (Pocock 1895), the gonopod of this species looks absolutely different from the *Orthomorpha* type, the solenomere showing a couple of denticles near the base, while the solenophore (= lamina lateralis) is produced dorsally before subtending the solenomere. Such a conformation resembles

that of a species of the mainly Papuan tribe Eustrongylosomatini, but the only member of this tribe known to occur west of Borneo is *Diglossosternoides curiosus*, from the Seychelles (see above). Both Attems (1937) and Jeekel (1968) referred to *Orthomorpha oatesii* as a species incertae sedis, but Golovatch (1998) included it into his key to *Orthomorpha* species for the sake of completeness. Revision of *Orthomorpha oatesii* is required before this species can be properly allocated. The holotype comes from an unspecified locality in southern Tenasserim (= Tanintharyi), Myanmar (Pocock 1895).

### Orthomorphoides gen. n.

urn:lsid:zoobank.org:act:5AC4D46F-DA56-43BD-9983-A88D60F627A1

http://species-id.net/wiki/Orthomorphoides

#### Diagnosis.

A genus of Orthomorphini with 20 segments. Body small-sized, adults ca 18–20 mm long, ca 2.2–2.8 mm wide on midbody metazona. Paraterga moderately well developed, metazonite to prozonite width ratio being ca 1.3. Adenostyles missing. Sternal lobe or cone(s) between ♂ coxae 4 present or absent.

Gonopod with a long subcylindrical coxite and a usual, cylindrical cannula. Telopodite long and slender, modestly curved. Prefemoral part densely setose, more than 2 times shorter than femorite. The latter without evidence of torsion, slightly enlarged distally, without sulcus demarcating a “postfemoral” part. Solenophore consisting of subequally modestly developed laminae lateralis, and medialis, both sheathing a similarly long, simple, flagelliform solenomere with a barely exposed tip; tip of solenophore never deeply split, poorly bi- or trifid, at least some of its apical prongs being short spines.

#### Type-species.

*Orthomorpha setosa* Attems, 1937, by present designation.

Other species included: *Orthomorphoides exaratus* (Attems, 1953).

### Orthomorphoides setosus

(Attems, 1937) comb. n.

http://species-id.net/wiki/Orthomorphoides_setosus

Figs 116 117

Orthomorpha setosa Attems 1937: 71 (D).
Orthomorpha setosa – Attems 1938: 207 (D).
“Orthomorpha” setosa – Jeekel 1963: 267 (M); 1968: 56 (M).

#### Syntypes.

1 ♂, 1 ♀ (NHMW-3517), Vietnam, Lamdong Prov., Dalat, 1500 m, 02.1933, leg. C. Dawydoff.

#### Non-type.

1 ♂ (NHMW-7987), Vietnam, Lamdong Prov., Dalat, Peak Lang Biang, 1200–2400 m, no date, leg. C. Dawydoff, det. C. Attems. This sample was erroneously labeled as a syntype, which cannot be such, because only material from Dalat was referred to in the available descriptions (Attems 1937, 1938).

#### Redescription.

Length ca 18 mm (♂) or 20 mm (♀), width of midbody pro- and metazona 2.0 and 2.5 mm (♂), 2.2 and 2.8 mm (♀), respectively.

**Figure 116.**
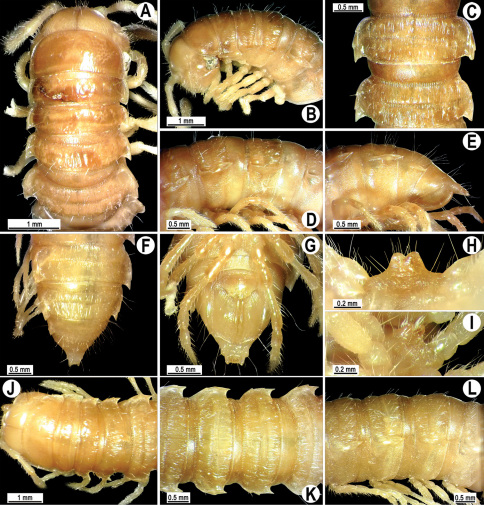
*Orthomorphoides setosus* (Attems, 1937), comb. n., ♂ syntype (**A–I**), ♀ syntype (**J–L**). **A, B, J** anterior part of body, dorsal, lateral and dorsal views, respectively **C, D, K, L** segments 10 and 11, dorsal, lateral, dorsal and lateral views, respectively **E-G** posterior part of body, lateral, dorsal and ventral views, respectively **H, I** sternal cones between coxae 4, subcaudal and sublateral views, respectively.

Coloration of alcohol material after long-term preservation faded to uniformly light brown (Fig. 116) (vs castaneous brown to light brown, as given in the available descriptions (Attems 1937, 1938)).

Clypeolabral region densely setose, vertex bare, epicranial suture distinct. Antennae short, poorly clavate (Fig. 116B), extending behind (♂) or not reaching end of segment 2 (♀) dorsally. Head in width < collum < segment 2 = 3 < 4 < 5–17 (♂, ♀); thereafter body gently and gradually tapering. Collum semi-lunar, with three transverse rows of setae traceable only as insertion points: 4(5)+4(5) anterior, 4+4 intermediate and 4+4 posterior; surface very slightly rugulose near caudal margin; paraterga subrectangular, with a small lateral incision at caudal 1/3; caudal corner nearly pointed, narrowly rounded, not extending behind tergal margin (Fig. 116A & B). Tegument shining, prozona very finely shagreened, metaterga rugose to rugulose (especially well so in caudal halves of metaterga), finely granulate, below paraterga microgranulate. Postcollum metaterga with 3–4 transverse, often irregular rows of medium-sized, sometimes abraded setae always traceable at least as insertion points, short wrinkles or knobs: 5–7+5–7 in front row on minute knobs and 5–6+5–6 in second row (both pre-sulcus), as well as 6–8+6–8 in both subcaudal and caudal rows (both postsulcus ones) on wrinkles or knobs (Fig. 116A-G & J-L). Axial line rather clear, especially so on metaterga, starting from collum. Paraterga moderately strongly developed (Fig. 116A-G & J-L), nearly always spiniform and pointed, mostly subhorizontal, set low at about 1/2 midbody height, lying well below dorsum; shoulders strongly and regularly rounded, well-developed, caudal corners of postcollum paraterga always slightly extending beyond tergal margin. Calluses delimited by a sulcus only dorsally, narrow, with four small lateral incisions on callus 2 and three similarly unclear indentations on each following segment (Fig. 116A-F & J-L). Posterior edge of paraterga always evidently concave (Fig. 116E-G & L). Ozopores evident, lateral, lying in a deep ovoid groove at about 1/3–1/4 paratergal length in front of caudal corner. Transverse sulcus present on metaterga 5–18, complete and reaching bases of paraterga, incomplete and faint on metaterga 4 and 19 (♂, ♀), beaded at bottom, rather deep and (Fig. 116A, C, F & J-L). Stricture between pro- and metazona rather shallow, narrow, evidently beaded at bottom down to base of paraterga. Pleurosternal carinae poorly developed, especially so in ♀, as small, complete and roughly granulate crests with a distinct tooth both frontally and caudally only on segments 2 and 3, traceable as a small caudal denticle on segment 4, thereafter missing (♂, ♀) (Fig. 116B). Epiproct (Fig. 116E-G) conical, rather short, flattened dorsoventrally, with evident apical papillae directed caudally and with two very strong pre-apical papillae not very strongly removed from an emarginate tip. Hypoproct (Fig. 116G) semi-circular, setiferous knobs at caudal edge very small and moderately well separated.

Sterna sparsely setose; a large central lobe with a paramedian pair of evident, subcontiguous, anteroventrally directed, setose cones between ♂ coxae 4 (Fig. 116I & J); a pair of small paramedian tubercles in front of gonopod aperture. Legs short and slender, almost not incrassate in ♂, midbody ones ca 0.9–1.1 (♂) or 0.7–0.9 times (♀) as long as body height, prefemora without modifications, ♂ tarsal brushes present until legs of ♂ segment 9.

**Figure 117.**
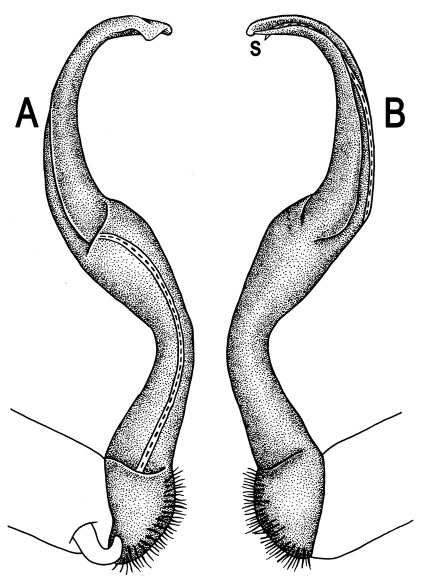
*Orthomorphoides setosus* (Attems, 1937), comb. n., ♂ syntype. **A, B** left gonopod, mesal and lateral views, respectively.

Gonopods (Fig. 117) simple. Coxa long and slender, without setae. Prefemoral part densely setose, more than 2 times shorter than femorite. The latter about as long as solenophore, slender, strongly curved, enlarged distad, without sulcus demarcating a postfemoral part. Solenophore tip clearly bifid, with a subterminal spine (**s**) and a terminal lobule; solenomere long and flagelliform, tip a little exposed.

#### Remarks.

Jeekel (1968) has long separated both *Orthomorpha setosa* Attems, 1937, from Vietnam, and *Pratinus exaratus* Attems, 1953, from Laos, as warranting a genus of their own. Now that we have been privileged to revise the type material of the former species, this issue is being formalized by proposing a new genus, *Orthomorphoides* gen. n., to incorporate both *Orthomorpha setosus* (Attems, 1937), comb. n., and *Orthomorpha exaratus* (Attems, 1953), comb. n.

*Orthomorpha exaratus* differs readily from *Orthomorpha setosus* as follows. Collum with paraterga spininform and extending behind tergal margin. Paraterga with only two lateral incisions. Three rows of setae on metaterga: one in front, the other two behind sulcus. Lobe between ♂ coxae 4 missing. Gonopod femorite slenderer. Type locality: Xieng Kuang Park, Vientiane, Laos (Attems 1953).

## Key to Orthomorpha species, based mainly on ♂ characters

**Table:** 

1	Gonopod tip like a single, very small, rounded lobule (Figs 5 & 6). Virtually no modifications between ♂ coxae 4 (Fig. 4H & I). Pantropical	*Orthomorpha coarctata*
–	Gonopod tip mainly bi- to trifid. Modifications between ♂ coxae 4 mostly present. Local in Southeast Asia	2
2	Gonopod femorite about as long as prefemoral portion (Fig. 113)	*Orthomorpha butteli*
–	Gonopod femorite ca 2–3 times longer than prefemoral portion (e.g. Figs 83, 94 & 99)	3
3	No distinct lateral sulcus on gonopod femorite demarcating a “postfemoral” region, at most a very faint one; solenophore tip neither acuminate nor branching. Thailand and northernmost Malaysia	4
–	A distinct lateral sulcus on gonopod femorite demarcating a “postfemoral” region; solenophore tip either acuminate or rather evidently branching (e.g. Figs 30, 33 & 46)	9
4	At least surface of metaterga leathery, dull	5
–	Dorsal surface shining	7
5	Only caudal parts of paraterga contrasting light. Paraterga less strongly developed, narrower, with only a small incision at best in front 1/3 extent. No tergal setae borne on evident knobs/tubercles	*Orthomorpha sericata*
–	Entire paraterga contrastingly light, much broader, largely with an evident, dentiform incision in front 1/3 extent. At least some tergal setae borne on evident knobs/tubercles	6
6	Surface of metaterga rough, microgranulate and rugulose. Metatergal setigerous tubercles higher, present also on collum. Paratergal incisions especially prominent	*Orthomorpha enghoffi*
–	Surface of metaterga nearly smooth, microgranulate. Metatergal setigerous tubercles evidently smaller, absent from collum. Paratergal incisions evident, but not so prominent	*Orthomorpha alutaria*
7	Adults > 30 mm long and > 4 mm wide. Collum broadly rounded laterally. Paraterga set high, at least some upturned above dorsum. Sternal cones between ♂coxae 4 isolated. Lateral sulcus on gonofemorite traceable, but very faint	*Orthomorpha parasericata*
–	Adults < 30 mm long and < 4 mm wide. Collum narrowly rounded to angulate laterally. Paraterga set high, but remaining below dorsum even in male. Sternal cones between ♂ coxae 4 fused basally into a single lamina. Lateral sulcus on gonofemorite absent	8
8	Bases of paraterga more broadly light. Legs shorter, midbody ones ca 1.2 (♂) or 1.0 times (♀) as long as body height. Tip of solenophore like an elongate and undulate lobe	*Orthomorpha subsericata*
–	Bases of paraterga more narrowly light. Legs longer, midbody ones ca 1.5 (♂) or 1.2–1.3 times (♀) as long as body height. Tip of solenophore tridentate	*Orthomorpha asticta*
9	Gonopod tip bifid (Figs 10, 12, 18 & 36)	10
–	Gonopod tip trifid, albeit middle prong can be very small (Figs 44, 46, 52 & 61)	18
10	Metaterga smooth and shining, at most faintly rugulose (Figs 11 & 25)	11
–	Metaterga at least with one rather evident, transverse row of tubercles near caudal margin (Figs 9, 13, 28 & 34)	13
11	Gonopod femorite slightly, but evidently twisted near base; tip rather deeply split, both terminal and subterminal prongs spiniform	*Orthomorpha paviei*
–	Gonopod femorite not twisted near base; tip shallowly bifid, both terminal and subterminal prongs dentiform (e.g. Figs 12, 26 & 27)	12
12	Length ca 19–23 mm, width of midbody pro- and metazona 1.8 and 2.7 mm (♂), 2.3–2.5 and 3.4–3.5 mm (♀), respectively. Coloration of paraterga and epiproct creamy orange (live material) to faded pinkish or pale yellow (alcohol-preserved material) (Fig. 11). Similan Islands, Thailand (Map 2)	*Orthomorpha picturata* sp. n.
–	Length 31–38 mm, width of midbody pro- and metazona 2.6–4.0 and 4.2–4.7 mm (♂), 3.2–3.8 and 4.8–5.3 mm (♀), respectively. Coloration of paraterga and epiproct creamy yellow (both live and alcohol-preserved material) (Fig. 25). Continental Thailand (Map 2)	*Orthomorpha communis* sp. n.
13	Metaterga with only a single row of very small tubercle near caudal margin (Figs 28, 31 & 34)	14
–	Metaterga with two rows of tubercles: small ones in front of, larger ones behind sulcus (near caudal margin) (Figs 9, 13 & 22)	16
14	Caudal corners of anterior and midbody paraterga not extended behind tergal margin (Fig. 34A-D). Metaterga mostly flavous (Fig. 34A-F). Sternal cones between ♂ coxae 4 widely separated (Fig. 34H & I)	*Orthomorpha suberecta* sp. n.
–	Caudal corners of all postcollum paraterga evidently produced behind tergal margin. Metaterga mostly infuscate. Sternal cones between ♂ coxae 4 subcontiguous	15
15	Pleurosternal carinae complete high crests with a sharp caudal tooth on segments 2–7 (♂). Transverse sulcus visible on metaterga 5–18, narrow, not reaching bases of paraterga, ribbed at bottom. Stricture between pro- and metazona narrow and rather shallow, faintly beaded at bottom down to base of paraterga (Fig. 34B-F & K)	*Orthomorpha latiterga* sp. n.
–	Pleurosternal carinae complete high crests with a sharp caudal tooth on segments 2–4(5) (♂, ♀). Transverse sulcus evident (Fig. 28B-F & H), thin, deep and only slightly incomplete on metaterga 2–4, complete, at most very faintly beaded at bottom, reaching bases of paraterga on metaterga 5–18 (Fig. 28B, D & F)	*Orthomorpha atypica* sp. n.
16	A single sternal cone between ♂ coxae 4 (Fig. 9H & I). Vietnam	*Orthomorpha arboricola*
–	Two sternal cones between ♂ coxae 4 (Figs 13H, I, 22H & I). Thailand	17
17	Postcollum paraterga with a very strong front indentation laterally (Fig. 22a-F & J-L)	*Orthomorpha subtuberculifera* sp. n.
–	Postcollum paraterga with only a very small front indentation laterally (Fig. 13B-F)	*Orthomorpha tuberculifera* sp. n.
18	Paraterga mostly level to or even above dorsum	19
–	All paraterga below dorsum	28
19	Colour pattern not strikingly contrasting, calluses only being inconspicuously paler than a dark remaining background (Figs 41 & 109)	20
–	Coloration of metaterga more or less strongly contrasting, with very pale calluses against a very dark remaining background (Figs 49, 51 & 106)	21
20	Metaterga with two transverse rows of evident tubercles (Fig. 41A-F). Vietnam	*Orthomorpha scabra*
–	Metaterga quite smooth, without transverse rows of evident tubercles (Fig. 109B-F). Northern Malaysia	*Orthomorpha spiniformis* sp. n.
21	Calluses on metaterga rather thin (Figs 51A-F & 100A-F)	22
–	Calluses on metaterga rather thick (Fig. 49A-F)	23
22	Sternal cone between ♂ coxae 4 single, large (Fig. 51H & I). Southern Vietnam	*Orthomorpha glandulosa*
–	Two small sternal cones between ♂ coxae 4 (Fig. 100I & J). Northern Malaysia	*Orthomorpha elevata* sp. n.
23	Metaterga virtually smooth, at most faintly rugulose, only seldom (*Orthomorpha weberi*) with minute knobs caudolaterally (Fig. 49A-F & J-L)	24
–	Metaterga with a caudal row of evident tubercles	26
24	♂ tarsal brushes entirely wanting. Epiproct with very distinct apical papillae. Lombok Island, Indonesia	*Orthomorpha francisca*
–	♂ tarsal brushes present. Epiproct with small apical papillae. Java Island, Indonesia	25
25	Tarsal brushes present until ♂ midbody legs	*Orthomorpha weberi*
–	Tarsal brushes present until ♂ legs 7	*Orthomorpha tenuipes*
26	Adult body particularly small, only ca 2.6 mm in width (♂). Bali Island, Indonesia	*Orthomorpha baliorum*
–	Adult body larger, at least 3.5 mm in width. Java Island, Indonesia	27
27	Paraterga relatively strongly produced caudally, paratergal calluses in dorsal view rather strongly sinuate near ozopores	*Orthomorpha conspicua*
–	Paraterga relatively slightly produced caudally, paratergal calluses in dorsal view rather poorly sinuate near ozopores	*Orthomorpha beroni*
28	A row of very small tubercles near caudal margin of some metaterga (Figs 39A, C, F, 43A, C, F, J-L)	29
–	Metaterga virtually smooth, at best only slightly rugulose near caudal margin (Figs 53A-F, 58B-F)	31
29	Small tubercles present only on metaterga 10–18(19)	*Orthomorpha flaviventer*
–	Small tubercles present on all postcollum metaterga (Figs 39A-F, 43A-F & J-L)	30
30	Paraterga more strongly produced caudally (Fig. 39A-G). Java Island, Indonesia	*Orthomorpha unicolor*
–	Paraterga less strongly produced caudally (Fig. 43A-G & J-L). Vietnam	*Orthomorpha rotundicollis*
31	A single sternal lobe between ♂ coxae 4^1^ (Fig. 53H & I)	32
–	Two sternal cones, sometimes very low ones, between ♂ coxae 4 (Figs 65I, J, 81H, I, 106I & J)	33
32	Sternal lobe between ♂ coxae 4 subquadrate. Perak State, southern Malaysia	*Orthomorpha bipunctata*
–	Sternal lobe between ♂ coxae 4 divided into two lobules only apically (Fig. 53H & I). Java Island, Indonesia	*Orthomorpha zehntneri*
33	Metaterga and collum usually more or less flavous, with lighter bands, spots or markings, at least in caudal parts (Figs 64A-F, 70A-F, 75A-G & K-M)	34
–	Metaterga and collum between paraterga usually completely dark (Figs 37A-F, J-L, 106A-F)	41
34	Pore-bearing midbody paraterga particularly thick in lateral view (Figs 75E & 91E)	35
–	Pore-bearing midbody paraterga considerably thinner in lateral view (Figs 65E, 69D & 86D)	36
35	Paraterga set mostly slightly higher (Figs 91E, H, 92E & H). Transverse sulcus present on metaterga 4–19. Tarsal brushes present until ♂ legs of segment 16	*Orthomorpha insularis*
–	Paraterga set mostly slightly lower (Figs 74B, D, E, 75C, E & H). Transverse sulcus present on metaterga 5–18(19). Tarsal brushes present until ♂ legs of segment 13	*Orthomorpha subkarschi*
36	Entire collum yellow, colour pattern like in Figs 70, 87 or 97, with at least a small patch remaining light near caudal margin	*Orthomorpha karschi*
–	Collum and subsequent tergites between paraterga at least partly infuscate, only postsulcus halves of metaterga often pallid to pale brown	37
37	No dark axial stripe on metaterga. Paraterga less prominent, their anterolateral margin relatively narrowly rounded (Figs 87 & 97)	38
–	A dark axial stripe or line on metaterga evident. Paraterga much more prominent, their anterolateral margin very broadly rounded (Figs 65, 70 & 81)	39
38	Colour pattern as in Fig. 97. Calluses on paraterga narrow. Sternal cones between ♂ coxae 4 very small and broadly separated (Fig. 97H & I). Central Thailand	*Orthomorpha isarankurai* sp. n.
–	Colour pattern as in Figs 86 & 87. Calluses on paraterga considerably broader. Sternal cones between ♂ coxae 4 much higher and poorly separated (Fig. 87G & H). Southern Thailand	*Orthomorpha lauta*
39	Sternal cones between ♂ coxae 4 smaller (Figs 81H, I, 82I & J). Caudal denticle of pleurosternal carinae traceable until ♂ segment 16 or 17	*Orthomorpha thalebanica*
–	Sternal cones between ♂ coxae 4 larger (Figs 64I & 70I). Caudal denticle of pleurosternal carinae traceable until ♂ segment 13 or 14	40
40	Colour pattern of live animals as in Fig. 70A. Bases of paraterga in alcohol material mostly infuscate, brown to dark brown (Figs 69 & 70)	*Orthomorpha horologiformis*
–	Colour pattern of live animals as in Fig. 65A. Bases of paraterga in alcohol material flavous, brown to dark brown (Figs 64 & 65)	*Orthomorpha pterygota*
41	Postcollum metaterga flavous behind transverse sulcus (Figs 79 & 103)	42
–	Postcollum metaterga dark on both pre-sulcus and postsulcus halves (Figs 37, 58 & 106)	45
42	Paraterga with anterolateral corner considerably more angulate, more narrowly rounded	*Orthomorpha fuscocollaris*
–	Paraterga with anterolateral corner considerably more broadly rounded (Figs 79 & 103)	43
43	Metaterga nearly completely flavous, only a thin axial line and transverse sulcus forming an infuscate cross. King Island, Mergui Archipelago, Myanmar	*Orthomorpha crucifer*
–	Metaterga mostly dark, including bases of paraterga (Figs 79 & 103). Southern mainland Thailand	44
44	Pleurosternal carinae present at least as a caudal denticle until segment 12 (♂). Tarsal brushes present until about ♂ midbody legs	*Orthomorpha banglangensis*
–	Pleurosternal carinae present at least as a caudal denticle until segment 16 (♂) or 13 (♀). Tarsal brushes present until about ♂ legs 8	*Orthomorpha subelevata* sp. n.
45	Caudal corner of midbody calluses only modestly produced behind tergal margin (Figs 37C, D, K, 106D & E). Sternal cones between ♂ coxae 4 very small, nearly indistinguishable, widely separated (Figs 37H, I, 106I & J)	46
–	Caudal corner of midbody calluses very strongly produced behind tergal margin (Figs 2A-F, 47C, D, 55C, D, 57C & D). Sternal cones between ♂ coxae 4 mostly evident, higher and less strongly separated (Figs 2H, I, 57H, I, 62H & I)	47
46	Pleurosternal carinae complete crests on segments 2–4 (♂, ♀) (Fig. 37B, D & E), each with an evident sharp denticle caudally, thereafter increasingly strongly reduced until segment 17 (♂) or 16 (♀). Tarsal brushes present on ♂ legs 1–7	*Orthomorpha hydrobiologica*
–	Pleurosternal carinae complete crests bulged anteriorly and with a sharp caudal tooth on segments 2–7, thereafter only a sharp caudal tooth on segments 8–16 (♂), or crests bulged anteriorly and with a sharp caudal tooth on segments 2–4, thereafter only a small sharp caudal tooth on segments 5–14 (♀). Tarsal brushes present until legs of ♂ segment 13	*Orthomorpha similanensis* sp. n.
47	Caudal corner of paraterga evidently rounded (Fig. 55A-F). Sternal cones between ♂ coxae 4 high and strongly separated (Fig. 55H & I). Environs of Kuala Lumpur, Malaysia	*Orthomorpha fluminoris*
–	Caudal corner of paraterga evidently pointed (Figs 2A, C, F, 47A-G, J-L & 57A-G). Sternal cones between ♂ coxae 4 usually much lower, if not, then both fused at base (Figs 47H, I, 57H, I, 62H & I)	48
48	Paraterga especially strongly produced behind tergal margin (Figs 57 & 58). Sternal cones fused at base (Figs 57H, I, 58I & J). Singapore	*Orthomorpha murphyi*
–	Paraterga much less strongly produced behind tergal margin (Figs 2A-G, 47A-G & 62A-G). Sternal cones not fused at base (Figs 2H, I, 47H, I, 62H & I)	49
49	Transverse metatergal sulcus present only on segments 5–18 (Fig. 2A, C & F). Pulau Karimunjawa and ?Java islands, Indonesia	*Orthomorpha beaumontii*
–	An incomplete transverse metatergal sulcus traceable also either on segments 3 and 4 or only on segment 4	50
50	Transverse metatergal sulcus traceable on segments 3 and 4, fully developed on segments 5–18 (Fig. 62A, C & F). Sternal cones between ♂ coxae 4 rather poorly separated (Fig. 62H & I). Sumatra Island, Indonesia	*Orthomorpha melischi*
–	Transverse metatergal sulcus incomplete only on segment 4, complete on segments 5–18 (Fig. 47A, C, F & J-L). Sternal cones between ♂ coxae 4 strongly separated (Fig. 47H & I). Cambodia	*Orthomorpha cambodjana*

**1** Unknown in *Orthomorpha fuscocollaris*, a species based only on ♀♀.

## Conclusion

The genus *Orthomorpha* as currently defined is the largest in the family Paradoxosomatidae in Southeast Asia, at present comprising 51 identifiable species. Some of them are still rather poorly distinguished from their superficially most similar congeners. In particular, such species pairs as *Orthomorpha lauta* vs *Orthomorpha insularis* or *Orthomorpha horologiformis* vs *Orthomorpha pterygota* appear to be quite variable morphologically, while their distributions show no considerable gaps (Map 4). So molecular analyses, for instance bar-coding, would be most desirable in order to shed additional light on the identities and affinities of such variable species.

There can be no doubt that further collecting efforts, especially in still very poorly explored regions as Laos, Myanmar and Cambodia, also some parts of Vietnam, will reveal many more species of *Orthomorpha*. Only Thailand and northern Malaysia can be said to be fairly well covered in this respect, particularly so after the present revision. At present we can no longer suggest any infrageneric classification better than one based on the structure of the gonopod tip, and we abandon a division into species groups altogether.

## Supplementary Material

XML Treatment for Orthomorpha

XML Treatment for Orthomorpha beaumontii

XML Treatment for Orthomorpha coarctata

XML Treatment for Orthomorpha arboricola

XML Treatment for Orthomorpha picturata

XML Treatment for Orthomorpha tuberculifera

XML Treatment for Orthomorpha tuberculifera

XML Treatment for Orthomorpha tuberculifera

XML Treatment for Orthomorpha subtuberculifera

XML Treatment for Orthomorpha communis

XML Treatment for Orthomorpha atypica

XML Treatment for Orthomorpha latiterga

XML Treatment for Orthomorpha suberecta

XML Treatment for Orthomorpha paviei

XML Treatment for Orthomorpha hydrobiologica

XML Treatment for Orthomorpha unicolor

XML Treatment for Orthomorpha scabra

XML Treatment for Orthomorpha rotundicollis

XML Treatment for Orthomorpha cambodjana

XML Treatment for Orthomorpha tenuipes

XML Treatment for Orthomorpha glandulosa

XML Treatment for Orthomorpha zehntneri

XML Treatment for Orthomorpha fluminoris

XML Treatment for Orthomorpha murphyi

XML Treatment for Orthomorpha melischi

XML Treatment for Orthomorpha pterygota

XML Treatment for Orthomorpha horologiformis

XML Treatment for Orthomorpha subkarschi

XML Treatment for Orthomorpha banglangensis

XML Treatment for Orthomorpha thalebanica

XML Treatment for Orthomorpha lauta

XML Treatment for Orthomorpha insularis

XML Treatment for Orthomorpha crucifer

XML Treatment for Orthomorpha karschi

XML Treatment for Orthomorpha conspicua

XML Treatment for Orthomorpha weberi

XML Treatment for Orthomorpha fuscocollaris

XML Treatment for Orthomorpha flaviventer

XML Treatment for Orthomorpha bipunctata

XML Treatment for Orthomorpha francisca

XML Treatment for Orthomorpha sericata

XML Treatment for Orthomorpha baliorum

XML Treatment for Orthomorpha beroni

XML Treatment for Orthomorpha subsericata

XML Treatment for Orthomorpha alutaria

XML Treatment for Orthomorpha asticta

XML Treatment for Orthomorpha enghoffi

XML Treatment for Orthomorpha parasericata

XML Treatment for Orthomorpha isarankurai

XML Treatment for Orthomorpha elevata

XML Treatment for Orthomorpha subelevata

XML Treatment for Orthomorpha similanensis

XML Treatment for Orthomorpha spiniformis

XML Treatment for Orthomorpha butteli

XML Treatment for Orthomorpha herpusa

XML Treatment for “Orthomorpha” crinita

XML Treatment for “Orthomorpha” variegata

XML Treatment for “Orthomorpha” bisulcata

XML Treatment for “Orthomorpha” coxisternis

XML Treatment for “Orthomorpha” oatesii

XML Treatment for Orthomorphoides

XML Treatment for Orthomorphoides setosus
